# Highly Efficient Site-Specific and Cassette Mutagenesis of Plasmids Harboring GC-Rich Sequences

**DOI:** 10.3390/cells14242016

**Published:** 2025-12-18

**Authors:** Paulina Varela-Castillo, Ethan Zhou, Arezousadat Razavi, Elham Ebrahimi, Xiang-Jiao Yang

**Affiliations:** 1 Rosalind and Morris Goodman Cancer Institute, McGill University, Montreal, QC H3A 1A3, Canada; 2 Department of Medicine, McGill University, Montreal, QC H3A 1A3, Canada; 3 Department of Biochemistry, McGill University, Montreal, QC H3A 1A3, Canada; 4 Department of Medicine, McGill University Health Center, Montreal, QC H3A 1A3, Canada

**Keywords:** G-quadruplex, multisite mutagenesis, ClinVar variant, neurodevelopmental disorder, cancer, SARS-CoV-2, genome editing, liquid–liquid phase separation

## Abstract

**Highlights:**

**What are the main findings?**

- This study reports that P3 and P3a site-directed mutagenesis methods are inefficient with plasmids possessing extremely GC-rich sequences, perhaps due to guanine (G)-quadruplex formation.
- To overcome this problem, we have developed P3b site-specific mutagenesis, which is highly efficient for plasmids with GC-rich regions, as demonstrated for the expression vectors of KAT2B, CDK13 and Cas9.

**What are the implications of the main findings?**

- Before embarking on site-directed mutagenesis experiments, it is recommended to analyze the GC-content distribution of template plasmids to select P3a or P3b mutagenesis.
- These two methods complement each other and provide a versatile approach for single-site, multisite or cassette mutagenesis, thereby offering two reliable tools for protein, RNA and plasmid engineering.

**Abstract:**

GC-rich sequences affect DNA replication, recombination and repair, as well as RNA transcription in vivo. Such sequences may also impede site-directed mutagenesis in vitro. P3a site-directed mutagenesis is a highly efficient method, but it has not been tested with plasmids possessing GC-rich sequences. Here we report that it is very efficient with a BRPF3 expression vector but unsuccessful with that for KAT2B. Because two GC-rich regions located within the synthetic CAG promoter and the KAT2B coding region may form guanine (G)-quadruplexes and hinder plasmid denaturation during PCR, we developed P3b site-specific mutagenesis, achieving an average efficiency of 97.5% in engineering ten KAT2B mutants. Importantly, deletion mutagenesis revealed that either of the two GC-rich regions is sufficient for rendering the plasmid incompatible with P3a mutagenesis. Consistent with this, only P3b mutagenesis worked efficiently with several widely used sgRNA/Cas9 expression vectors, which contain the CAG promoter, and with an expression vector for CDK13, which possesses an intrinsically disordered domain encoded by a GC-rich DNA fragment. Thus, this study highlights serious challenges posed by GC-rich sequences to site-directed mutagenesis and provides an effective remedy to address such challenges. The findings support that G-quadruplex formation is one mechanism whereby such sequences impede regular PCR-based mutagenesis methods.

## 1. Introduction

Since its initial report in 1978 [1], site-directed mutagenesis has been widely used for engineering gene mutations at specific sites in vitro. The QuickChange^™^ site-directed mutagenesis method has been used in many laboratories and is based on Pfu DNA polymerase-mediated PCR with a pair of complementary primers, followed by DpnI digestion to selectively degrade wild-type plasmids methylated at GATC sites recognized by DpnI [2,3,4]. An alternative approach is to utilize a pair of partially complementary primers with 3′-protruding ends [5,6,7]. This innovative approach addresses limitations such as primer–primer dimerization or oligomerization from self-annealing of complementary primers, the inability to use newly synthesized plasmid DNA strands as templates for further amplification, and the occurrence of unwanted mutations at the primer sites frequently observed with the QuickChange^™^ method [8,9]. We have recently optimized this innovative approach, referred to as P3 (primers with 3′-protruding ends) site-directed mutagenesis, and evaluated it with >100 mutations on a dozen expression vectors [8,9]. Compared to the QuickChange^™^ method, the success rate increases significantly and requires much less time for engineering various point mutations. The efficiency is ~50%, thus requiring further improvement [8,9]. As a result, we have recently developed P3a site-directed mutagenesis by replacing Pfu with SuperFi II and Q5 polymerases, which enhanced the efficiency to or close to 100% [10]. These two polymerases are superior to Pfu in terms of fidelity, processivity and synthesis speed. They are the best thermostable DNA polymerases now commercially available.

Notably, the P3 mutagenesis method encountered challenges with the expression plasmids for BRPF3 and KAT2B [8,9]. In particular, the method failed with the KAT2B plasmid [8,9], raising the important question whether P3a site-directed mutagenesis is effective towards the BRPF3 and KAT2B plasmids. We report here that while the P3a method was highly efficient with the BRPF3 expression plasmid, it was unsuccessful with the KAT2B expression plasmid. Sequence analysis of this plasmid revealed two extremely GC-rich regions: (i) the artificial CAG promoter possessing a GC-rich β-actin promoter; and (ii) a 0.27 kb GC-rich fragment encoding an intrinsically disordered domain of KAT2B rich in alanine, glycine and proline residues (Figures S1 and S2). Related to this, guanine-rich sequences can form G-quadruplexes [11] and stall DNA replication [12]. We thus hypothesized that these two GC-rich regions hinder efficient plasmid denaturation and impair P3a site-directed mutagenesis.

Overcoming this problem, we have now developed P3b site-specific mutagenesis by modifying the PCR parameters in two aspects: (1) incubating plasmids at 105 °C for 5 min and transferring them quickly to ice before proceeding to PCR-based mutagenesis; and (2) raising the denaturation temperature during PCR amplification. With SuperFi II but not Q5 DNA polymerase, this new method was highly efficient towards the KAT2B plasmid. To pinpoint whether the GC-rich regions are indeed the culprit, we carried out deletion analysis. The results indicate that either region is sufficient for rendering the vector incompatible with P3a mutagenesis. To investigate the applicability of the findings to other plasmids with GC-rich sequences, we analyzed widely used sgRNA/Cas9 expression plasmids that possess the CAG promoter. The results confirmed that P3b but not P3a mutagenesis is highly efficient for such plasmids. Notably, our results support that G-stretches but not C-rich sequences in the CAG promoter stall DNA synthesis in vitro, which agrees with a recent study showing that G-stretches form G-quadruplexes and impede DNA replication [11].

We have also evaluated a CDK13 expression plasmid because, similar to KAT2B, this kinase harbors an intrinsically disordered domain encoded by a GC-rich DNA fragment [13,14]. Serendipitously, from analysis of this plasmid, we found that a 39 bp GC-rich region at the 5′-region of an IRES sequence and the coding sequence of the puromycin-resistant marker pose challenges to P3a mutagenesis. However, P3b mutagenesis was highly efficient with plasmids containing such sequences. Therefore, our findings support that P3b mutagenesis is superior to the P3a method for plasmids containing GC-rich sequences. All current site-directed mutagenesis methods are limited to single-site mutations [5,6,8,9,10], so an important question is how to engineer multisite mutations rapidly. Related to this, we implemented a strategy to utilize P3a and P3b mutagenesis for achieving this goal. Thus, this study presents a highly efficient method for site-directed mutagenesis of plasmids that possess GC-rich sequences, such as the CAG promoter present in many mammalian expression vectors. In addition to traditional targets, this method will facilitate drug development stemming from recent advances in artificial intelligence (AI)-assisted protein structural prediction and de novo design [15,16,17,18,19].

## 2. Materials and Methods

### 2.1. Plasmids

The mammalian expression vector for HA-BRPF3 was described previously [20,21,22]. The plasmid for expressing FLAG-KAT6A in mammalian cells was kindly provided by Issay Kitabayashi [23]. The mammalian expression plasmids for untagged p300 and FLAG/HA-tagged mouse CBP were purchased from Addgene (Cat. 23252 and 32908, respectively, Addgene, Watertown, MA, USA). Modifications of these two plasmids for p300 and CBP have been described [8,10]. pCX-KAT2B, the expression plasmid for FLAG-tagged human KAT2B (also known as. PCAF), was previously described [24] and has been deposited into Addgene (Cat. 249547, Addgene, MA, USA). Compared to a related plasmid (Cat. 8941, Addgene, MA, USA), pCX-KAT2B possesses a recombinant CAG promoter, composed of the cytomegalovirus early enhancer element; the promoter, the first exon and the first intron of the chicken β-actin gene; and the splice acceptor of the rabbit β-globin gene [24,25]. The DNA sequence of the pCX promoter is based on that in pCX-cMyc (Cat. 19772, Addgene, MA, USA). The mammalian expression vector for FLAG-tagged CDK13 was obtained from Addgene (Cat. 135276, deposited by Ben Major and colleagues, Addgene, MA, USA). When this plasmid was isolated from bacterial cultures inoculated from a bacterial soft agar stab provided by Addgene, the yield was very low, perhaps due to its large size (12.7 kb) and/or the quality of the bacterial stock. The plasmid yield became normal when fresh colonies from retransformation into DH5α were inoculated for growing bacterial cultures and plasmid isolation. Mammalian expression vectors for untagged D614G and Omicron spike proteins of SARS-CoV-2 were purchased from SinoBiological (Cat. VG40589-UT and VG40835-UT, respectively; SinoBiological, Beijing, China).

### 2.2. GC-Content Calculation

The coding sequences of human BRD1, BRPF3 and KAT2B were copied from SnapGene (or directly from GenBank) and pasted into the calculation box provided by the VectorBuilder website (https://en.vectorbuilder.com/tool/gc-content-calculator.html (accessed on 18 May 2025)) or NovoPro website: (https://www.novoprolabs.com/tools/gc-content (accessed on 18 May 2025)). The GC-content was calculated with a window size set at 20. In addition, there is a GC-content display function within the SnapGene software package (version 8.0.2). Under the map view, the user clicks the ‘color options’ pulldown menu and selects the ‘GC-content’ option. Under the sequence view, this selection colors G or C in red (and A or T in light blue), which is very helpful for manual inspection of GC distribution across a plasmid sequence.

### 2.3. Heat or Alkali Denaturation of a KAT2B Expression Plasmid

For heat denaturation, 20 μL of the FLAG-KAT2B expression plasmid (0.1 μg/μL in 1× NEB digestion buffer #2, New England Biolabs, Ipswich, MA, USA) was prepared in a sterile 0.5 mL Eppendorf tube and incubated at a heating block set to 105 °C for 5 min. The tube was transferred onto ice for rapid cooling. The heat-denatured DNA was used immediately or stored at −20 °C for a few weeks. For alkali denaturation, 20 μL of the FLAG-KAT2B expression plasmid (0.1 μg/μL with autoclaved Nanopure water) was prepared in a sterile 0.5 mL Eppendorf tube, mixed with 2 μL NaOH (2 M) and incubated at 65 °C for 5 min as described [26]. The tube was transferred to a rack at room temperature and 2 μL HCl (2 M) was added for neutralization. After that, the tube was kept on ice for immediate use of the alkali-denatured DNA. Alternatively, the denatured DNA was stored for a few weeks at −20 °C. We also tested the addition of 10% DMSO in PCR reactions, but it did not improve the results. For the BRPF3 expression plasmid, this condition caused frequent deletions at the primer sites when the R15W mutant was engineered.

### 2.4. Isolation of Uracil-Containing Plasmids

Expression plasmids for BRPF3, KAT2B and the spike proteins of the D614G and Omicron variants of SARS-CoV-2 were transformed into CJ236 competent cells, prepared with an in-house protocol detailed previously [8]. The bacterial strain was originally purchased from Bio-Rad (Cat. 170-3114, Bio-Rad, Hercules, CA, USA). Single colonies were then used to inoculate 6 mL LB media containing ampicillin and chloramphenicol for subsequent minipreps using a Qiagen miniprep kit.

### 2.5. Mutagenic Primers

Primers were designed with the aid of the SnapGene software package (version 8.0). They were synthesized at Integrated DNA Technologies (IDT) as small-scale, standard and desalted DNA oligos, without further purification. They were prepared in 100 pmol/μL (i.e., 0.1 mM) stock solutions and 5–10 pmol/μL (i.e., 5–10 μM) working solutions in sterile Nanopure water; for storage, they were kept at −20 °C. Primers utilized to generate point mutants of BRPF3 and spike proteins of two SARS-CoV-2 variants have been described [8,10]. The primers to engineer two deletion mutants of BRPF3 are listed in Table S1. All mutagenesis primers for the spike proteins of SARS-CoV-2 variants have been described [8,10]. Sequencing primers were: V320-F, G566-R and Q965-F (Table S1).

3′-Overhang-containing primer pairs for engineering KAT2B point mutants via P3b mutagenesis are listed in Table S1. The primers to delete the coding sequence for the N-terminal 88 residues of KAT2B were dN88-F and dN88-R (Table S1). The primers to delete the coding sequence for the N-terminal 123 residues of KAT2B were: dN123-F and dN123-R (Table S1).

The sequencing primers for the CAG promoter were: CMV-F1 and KAT2B-R (Table S1). The primers to delete the 1.3 kb fragment encompassing the β-actin promoter and a chimeric intron on the KAT2B expression vector were: pCX-F, pCX-F1 and pCX-R (Table S1). Notably, when primer pCX-F was used with pCX-R, the mutagenesis efficiency was only 1 out of 18 colonies analyzed, i.e., 5.5%, with the remaining all wild-type. The colony number from the mutagenesis reaction was normal. This was the only instance where the efficiency was so low, so we inspected the primer sequences and found an error in the 5′-part of pCX-F, perhaps introduced due to an oversight when the primer was designed. The error was corrected in the new primer pCX-F1. When it was used with pCX-R, the mutagenesis efficiency was 100% (six out of six bacterial colonies analyzed). Other KAT2B mutagenic primers will be described elsewhere.

Primers for mutagenesis of the Cas9 (1.1) coding sequence are listed in Table S2. Other primers for mutating the genome-editing system has been described [10].

The primers for mutating the CDK13 expression plasmid are listed in Table S2. The sequencing primers for deletion of the 39 bp GC-rich upstream form the IRES sequence and the 0.6 kb puromycin-coding sequence were IRES-R and IRES-F (Table S2), respectively. Primers Ala162fs-F and Ala162fs-R did not yield any colonies when used for P3b mutagenesis, perhaps due to their possession of a stretch of Gs (Ala162fs-F) or Cs (in Ala162fs-R). To circumvent this problem, primers Ala162fs-F1 and Ala162fs-R1 were designed to disrupt the G- or C-stretches with some silent mutations. The two new primers functioned as expected in mediating site-specific mutagenesis. For the S229P mutation, neither Ala162fs-F nor Ala162fs-F1 could be used as effective sequencing primers, but S248N-R and V327M-R were good sequencing primers for detecting this mutation.

### 2.6. P3 Site-Directed Mutagenesis

P3 mutagenesis based on PfuUltra DNA polymerase (Cat. 600380, Agilent, Santa Clara, CA, USA) was carried out as described previously [8].

### 2.7. P3a Site-Directed Mutagenesis

PCR reactions were set up in 0.2 mL 8-strip thin-wall PCR tubes (Cat. DIATEC420-1378, Diamed, Mississauga, ON, Canada) as described [8,10]. Briefly, each reaction contained 0.15 μL of plasmid DNA (0.1 μg/μL), 0.5 μL forward/reverse primer mixture (5.0 pmol/μL or 5.0 μM for each primer), 4.35 μL autoclaved Nanopure water and 5 μL 2× Q5 hotstart (Cat. M0494S, New England Biolabs, Ipswich, MA, USA), Platinum SuperFi II (Cat. 12368010, Thermo Fisher Scientific, Waltham, MA, USA) or Q5U (Cat. M0597S, New England Biolabs, Ipswich, MA, USA) master mix in a total volume of 10 μL. The amplification was carried out in a Bio-Rad PCR T100 Thermal Cycler (Cat. 1861096, Bio-Rad, Hercules, CA, USA) using the following parameters: 96 °C for three minutes as the initial step to denature plasmid DNA, followed by 20–25 cycles to amplify the DNA. Each amplification cycle was composed of 93 °C for 15 s as the denaturation step, 52 °C for 20 s as the annealing step and 72 °C for 5–10 min as the extension step, where the extension time was calculated according to the plasmid size (20–30 s per kb). The amplification cycle number varied slightly from one plasmid to another, with the initial number set at 20, but the number was limited to the maximum of 25 to avoid or minimize unwanted mutations during PCR amplification. After amplification, an additional extension step of 72 °C for 7.5–10 min was added to the PCR program. Then the reaction was pre-programmed for short-term storage (such as a few hours) at 4 °C; for long-term storage, the reaction mixture was kept at −20 °C.

For DpnI digestion, 0.25 μL [20 units/μL] (Cat. R0176, New England Biolabs, MA, USA) was added to each reaction mixture, which was then transferred to a clean PCR tube and incubated at 37 °C in a Bio-Rad T100 Thermal Cycler (Cat. 1861096, Bio-Rad, CA, USA) for 90 min. The use of a clean PCR tube at this step was key to preventing contamination of the undigested plasmid from the inner walls of the previous PCR tube used for amplification. Transformation DH5α competent cells, plasmid isolation, sequencing and sequence analysis were performed as described previously [8,10].

### 2.8. P3b Site-Directed Mutagenesis

If needed, plasmids were denatured by heating at 105 °C for 5 min or incubation with 0.2 M NaOH as described above. For the latter, NaOH was neutralized with 0.2 M HCl. After denaturation, plasmids were used for PCR reactions as described above for P3a mutagenesis. However, the amplification was carried out in a PCR T100 Thermal Cycler (Cat. 1861096, Bio-Rad, CA, USA) using the following parameters: 98 °C for 2 min as the initial step to denature plasmid DNA, followed by 20–25 cycles to amplify the DNA. Each amplification cycle was composed of 98 °C for 10 s as the denaturation step, 52 °C for 20 s as the annealing step and 72 °C for 5–10 min as the extension step, where the extension time was calculated according to the plasmid size (20–30 s per kb). After amplification, an additional extension step of 72 °C for 5–10 min was added to the PCR program. The main difference in these PCR parameters from those in P3a mutagenesis is the denaturation temperature: it was 98 °C for the initial denaturation step and subsequent PCR cycles, whereas for P3a mutagenesis, the initial denaturation temperature was 96 °C and the subsequent denaturation temperature during the PCR cycles was 93 °C. Transformation of DH5α competent cells was performed as described previously [8,10].

### 2.9. Plasmid Sequencing and Analysis

Plasmid isolation, sequencing and sequence analysis were performed as described above [8,10]. Briefly, after isolation with a QIAprep^®^ Spin Miniprep Kit (Cat. 27104, Qiagen, Hilden, Germany), plasmids were sequenced at Genome Quebec technological service center. For sequencing reactions, plasmids were denatured at 96 °C, after which primer annealing was carried out at lower temperatures not exceeding 50 °C to allow hybridization of sequencing primers. Samples from the sequencing reactions were analyzed on a 96-capillary array DNA Sequencer 3730XL (Cat. A41046, Applied Biosystems, Foster City, CA, USA). The resulting sequences and chromatograms were transferred to SnapGene 8.0.1 for alignment analysis and manual inspection.

### 2.10. Statistics

Due to the qualitative nature of obtaining mutants in typical mutagenesis experiments, we focused on efficiency variations from mutation to mutation and from plasmid to plasmid. Standard deviations were computed via an online calculator: https://www.calculator.net/standard-deviation-calculator.html (accessed on 21 November and 5 December 2025).

## 3. Results

### 3.1. P3a Mutagenesis for Engineering Point and Deletion Mutants of BRPF3

Among over 10 expression plasmids that we have tested for P3 site-directed mutagenesis mediated by PfuUltra or Pfu-fly, the BRPF3 expression plasmid was significantly more challenging than the others [8]. For the rest of the plasmids tested, the overall mutagenesis efficiency was around 50% [8], thus still leaving some room for further improvement. It would be ideal to approach or reach the efficiency level of 100% as it would save labor and reagent costs. When PfuUltra and Pfu-fly were replaced with Q5 or SuperFi II polymerases, the resulting method, P3a site-directed mutagenesis, was much more efficient than P3 site-directed mutagenesis itself, reaching an efficiency at or near 100% [10]. Thus, an interesting question was whether this improved method is also highly efficient with the BRPF3 expression plasmid. To address this question, we initially used the new method to engineer the R15W, R51H and I52A mutants of BRPF3. It is noteworthy that for R15W, P3 site-directed mutagenesis reactions yielded no bacterial colonies when PfuUltra was used as the polymerase for PCR [8]. When Pfu-fly was used, a reasonable number of colonies was obtained [8]. However, unwanted deletions and/or insertions were frequently detected at the primer sites (Figure S3) [8], suggesting the following two possibilities: (1) unfaithful synthesis termination when a newly synthesized strand reaches the 5′-end of an annealed primer, or and (2) false priming at a wrong site.

**Figure 1:**
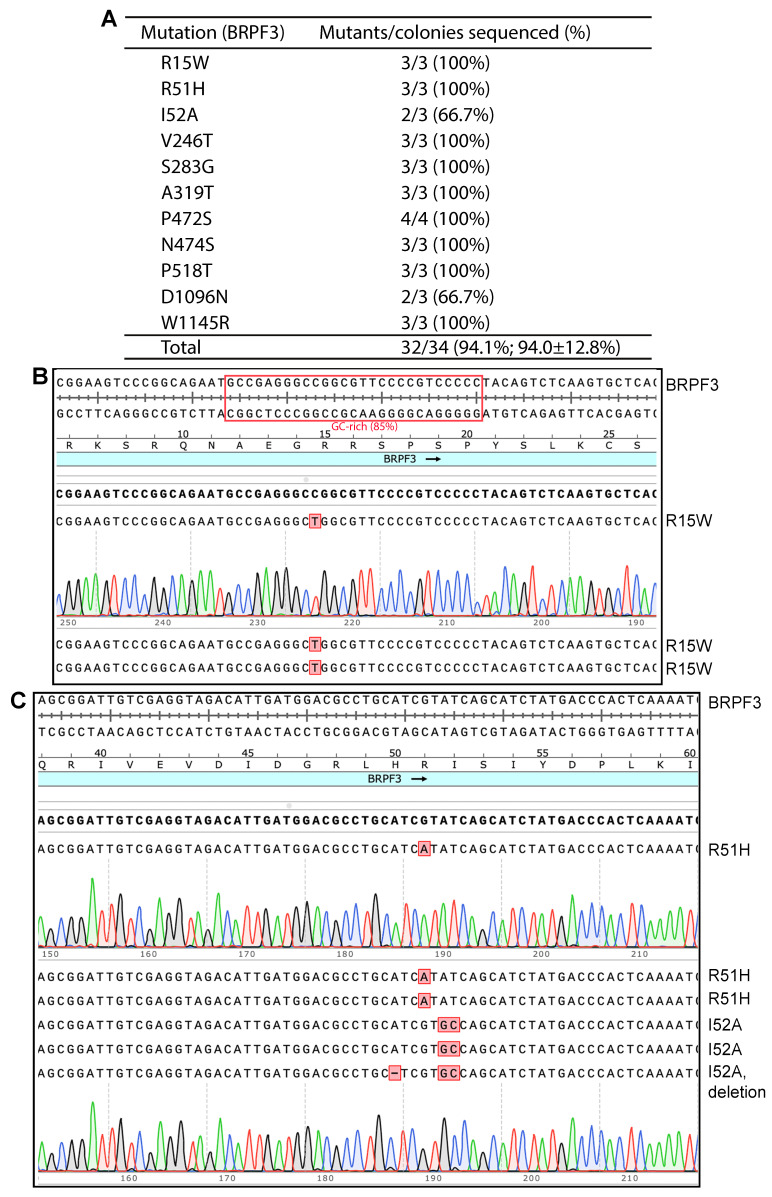
P3a mutagenesis with SuperFi II DNA polymerase to engineer BRPF3 mutants. (**A**) Efficiency of the mutagenesis method. SuperFi II DNA polymerase was used. Among 34 plasmids sequenced for generating 11 point mutants, only two were not the correct mutants. None corresponded to the wild-type. One I52A mutant contained an unexpected A deletion, potentially due to impurities in one of the two primers. A D1096N candidate could not be sequenced, likely due to an incorrect plasmid (such as the absence of the sequencing primer sequence), poor plasmid quality or sequencing issues. This candidate was counted as an incorrect mutant when the efficiency was calculated. (**B**) Analysis of three plasmids sequenced for engineering the R15W mutants. All three carried the correct point mutations, resulting in an ideal efficiency of 100%. A small GC-rich region is boxed in red. (**C**) Analysis of six plasmids sequenced for engineering the R51H and I52A mutants. Five harbored the correct mutations, yielding an average efficiency of 5/6 (83.3%).

As shown in Figure 1, when SuperFi II DNA polymerase was used, we were able to engineer the R15W, R51H and I52A mutants of BRPF3 at high efficiency: three colonies were sequenced per mutation, and eight out of the nine plasmids sequenced for all three mutations were correct. Importantly, for the R15W mutant, unwanted deletions or insertions observed with plasmids from the Pfu-fly condition [8] were not detected with this new method. Encouraged by these results, we engineered eight more point mutants. As shown in Figure 1A, we obtained all of them easily. For all 11 mutants, we sequenced plasmids from 34 bacterial colonies, with 32 of them containing the correct mutations. Among the remaining two, one I52A mutant candidate possessed an unexpected one-nucleotide deletion at the primer sites, perhaps due to impurity in the primers. The other was a D1096N candidate that failed to be sequenced, perhaps due to a wrong plasmid (such as the lack of primer sequence), poor plasmid quality or some technical issues related to Sanger sequencing. Nonetheless, this candidate was counted as an incorrect mutant. Thus, the overall efficiency was 32/34 (94.1%). These results also indicate that the quality of the DNA polymerase used for PCR is critical to achieve high mutagenesis efficiency. Amazingly, no wild-type colonies were found, suggesting that the presence of wild-type plasmid molecules is already minimal in the DpnI-digested mixtures. This aspect is crucial for comprehending the outcomes derived from the endeavors aimed at optimizing the conditions of mutagenesis, as delineated in the subsequent sections.

In addition to SuperFi II polymerase, Q5 DNA polymerase can be used for P3a mutagenesis [10]. Thus, we also investigated how efficient this enzyme is for generating BRPF3 mutants. In this case, we tested 4 BRPF3 mutants: R15W, R51H, I52A and K1075E. E1075K is an unexpected substitution present on the expression vector that we used, so K1075E was designed to repair the E1075K substitution. As shown in Figures S4C,D and S5, we sequenced 2–3 plasmids per mutation. Among the 12 plasmids that were sequenced, 10 carried the correct mutations. Among the remaining two, one was wild-type and the other carried an unexpected one-nucleotide deletion. Thus, the mutagenesis efficiency was 10/12 (83.3%, Figure S4B). This is within the range observed with the other 10 expression plasmids when P3a mutagenesis was used [10]. These results indicate that Q5 DNA polymerase can efficiently produce mutants from the BRPF3 expression plasmid. However, the efficiency may be slightly lower than SuperFi II polymerase (Figure 1). This minor difference aligns with the previous observations [10].

In addition to point mutants, P3a mutagenesis can be used for cassette mutagenesis to engineer deletion, insertion or fragment replacement [10]. To determine whether such BRPF3 mutants can be generated, we attempted to remove the 195 bp coding sequence for the N-terminal 65 residues of BRPF3 (Figure S6A), as this region is important for BRPF2 (a BRPF3 paralog) to interact with KAT7 [27]. Thus, we designed a pair of primers, dN65-F and dN65-R, as shown in Figure S6B. Then, P3a mutagenesis with SuperFi II DNA polymerase was carried out. Plasmids from three bacterial colonies were sequenced and all of them were correct (Figure S6C), yielding the efficiency of 100%. Similarly, we engineered another deletion mutant with the N-terminal 126 residues of BRPF3 removed. A different pair of primers, dN126-F and dN126-R, was designed similarly as shown in Figure S6B. P3a mutagenesis was then carried out. Plasmids from three of the resulting bacterial colonies were sequenced. Two of them were correct and the third was wild-type, resulting in an efficiency of 66.7%. Together, these results demonstrate that P3a cassette mutagenesis is also efficient for engineering deletion mutants of BRPF3.

### 3.2. Use of Uracil-Containing Templates in P3a Mutagenesis

For site-specific mutagenesis with plasmids, insertion of an F1 replication origin to isolate uracil-containing single-stranded phagemid DNA as the mutagenesis template was a popular method in the late 1980s and early 1990s [28,29,30,31]. This strategy has also been used for double-stranded plasmids [32]. Thus, an interesting question is whether uracil-containing templates can improve the efficiency of P3a mutagenesis. To test this strategy, we transformed the BRPF3 expression plasmid into the bacterial strain CJ236 to isolate uracil-containing DNA templates. This strain carries *ung*^-^ and *dut*^-^ mutations, rendering it deficient in uracil-DNA glycosylase and dUTP pyrophosphatase, two enzymes responsible for removing uracil from DNA during DNA replication and degrading dUTP, respectively [28]. As a result, DNA templates isolated from this strain contain a high amount of uracil [28,29,31]. We initially tested the following four thermostable DNA polymerases: PfuUltra, Pfu-fly, SuperFi II and Q5. However, mutagenesis reactions with these enzymes yielded few or no colonies, suggesting that they are either inactive or insufficiently active. To circumvent this problem, we then tested Q5U, an engineered version of Q5 polymerase known to work efficiently with uracil-containing templates. We tested the primers for the four BRPF3 mutants, R15W, R51H, I52A and K1075E, which have been described above for P3a mutagenesis with Q5 polymerase (Figures S4C,D and S5). Q5U polymerase yielded sufficient colonies, even though the colony counts were still 2–3 times lower than those achieved with the regular BRPF3 plasmid and Q5 polymerase. We sequenced plasmids from three colonies per mutation (four for R51H) and among the 13 plasmids sequenced for the four mutations, three plasmids were wild-type and three contained unexpected mutations, leading to an efficiency of 53.8% (Figures S4B–D and S5). Thus, Q5U works with the uracil-containing template but is much less efficient and reliable than Q5 polymerase with the regular BRPF3 expression plasmid.

To establish the general applicability of Q5U-based P3a mutagenesis, we tested two expression plasmids for the spike proteins of the D614G and Omicron variants of SARS-CoV-2 (Figure S7). For the D614G spike protein, we designed four pairs of primers to engineer the R346T, Q493E, L981F and V1104L mutations, which are present in Omicron variant or the JN.1 descendants and currently driving active infections around the world [33,34]. The expression plasmid for the spike proteins of the D614G variants was transformed into and isolated from CJ236 for P3a mutagenesis mediated by Q5U DNA polymerase. Per mutation, three plasmids were sequenced. Among the 12 plasmids sequenced for the four mutations, one was wild-type and the remaining 11 carried the correct mutations, yielding an efficiency of 91.7% (Figure S7A). We also tested the expression plasmid for the spike protein of the Omicron variant. The plasmid was also transformed into and isolated from CJ236 for P3a mutagenesis mediated by Q5U DNA polymerase. We utilized four pairs of primers for the following five mutants: R346T, Q493E, F456L, L455S/F456L and V1104L. As the F456L and L455S/F456L mutation sites overlap each other, we designed a single pair of primers for engineering both mutants in the same mutagenesis reaction. The F456L and L455S_F456L substitutions are present in JN.1 descendants [33,34]. Per mutagenesis reaction, three plasmids were sequenced. Among the 12 plasmids sequenced for the four mutagenesis reactions, only five carried the correct mutations, so the efficiency was 41.7% (Figure S7B). Together, these results indicate that P3a mutagenesis with Q5U is generally efficient but shows variability from plasmid to plasmid. Therefore, Q5U polymerase is less reliable than its wild-type counterpart, Q5 polymerase, with regular plasmid templates.

### 3.3. Developing P3b Mutagenesis for Efficient Generation of KAT2B Mutants

In the previous P3 mutagenesis reactions with PfuUltra or Pfu-fly polymerase [8], we were unable to obtain colonies when the expression plasmid for human KAT2B was used. Thus, as shown above for the BRPF3 expression plasmid (Figure 1), we investigated whether P3a mutagenesis [10] works with the KAT2B expression plasmid. Surprisingly, different from what was observed with the BRPF3 expression plasmid (Figure 1), the mutagenesis reactions with the KAT2B expression plasmid yielded complex results. When Q5 DNA polymerase was used, no colonies were obtained. When SuperFi II DNA polymerase was employed, there were some colonies, but the plasmids from the colonies could not be sequenced using KAT2B-specific primers, suggesting potential deletion. Indeed, double digestion with EcoRI and PstI revealed that the plasmids carried deletions encompassing the primer sites (Figure S8A–C), supporting that P3a mutagenesis encountered serious problems with this expression vector. One possible explanation is that this plasmid possesses special sequences impeding PCR amplification and thereby rendering it incompatible with P3a mutagenesis.

**Figure 2:**
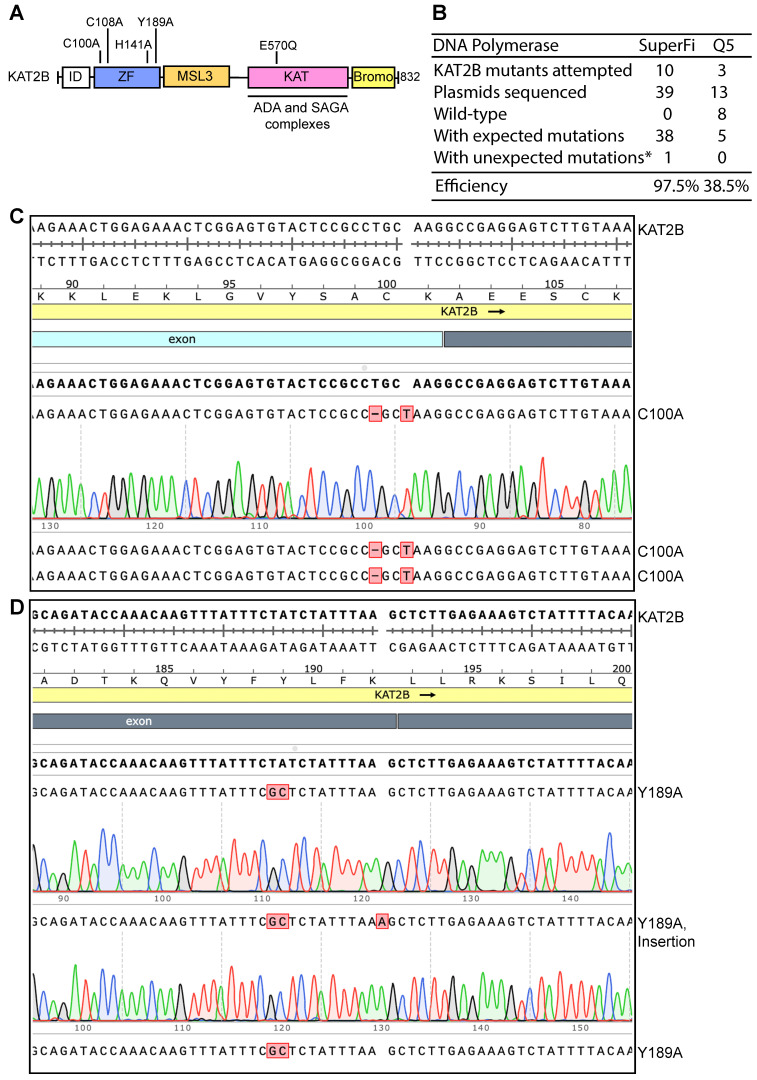
High efficiency of P3b site-specific mutagenesis to engineer KAT2B mutants. (**A**) Domain organization of KAT2B. Remaining poorly characterized, the N-terminal half of KAT2B is composed of one zinc finger and an MSL3-like domain. The coding sequence for the peptide from the N-terminus up to the zinc finger is extremely GC-rich, reaching 95–100% in certain areas (Figures S1C and S2A). This region encodes an Ala-, Gly- and Pro-rich intrinsic disorder (ID) domain, which has no known functions but may be involved in phase separation as reported for many other such domains [35]. The C-terminal half of KAT2B comprises two well-characterized domains: the acetyltransferase domain required for formation of several multiprotein complexes (including ADA and SAGA complexes) and the bromodomain important for recognition of acetylated chromatin [36]. Five point mutants are illustrated, with four located within the zinc finger region (C100A, C108A, H141A and Y189A) and one in the lysine acetyltransferase (KAT) domain (E570Q). The E570Q mutant is expected to inactivate the enzyme as E570 is a catalytic residue [37]. (**B**) Efficiency of P3b mutagenesis. Prior to PCR, the KAT2B expression plasmid was heat-denatured at 105 °C for 5 min and rapidly cooled on ice before PCR amplification with SuperFi II or Q5 DNA polymerase. We analyzed 3–4 plasmids per mutation. For the SuperFi II polymerase condition, we analyzed 39 plasmids from engineering 10 point mutants and all but one carried the correct mutations, resulting in the efficiency of 97.5%. For the Q5 polymerase condition, we analyzed 13 plasmids for engineering three point mutants and only five were the correct mutants. The remaining eight plasmids were wild-type, leading to an efficiency of 38.5%. * unwanted mutations from erroneous primer synthesis and/or nucleotide misincorporation during PCR. (**C**) Sequence chromatograms of three plasmids sequenced for engineering the C100A mutant of KAT2B with SuperFi II polymerase. These plasmids were from the SuperFi II polymerase condition. All three were correct, leading to an efficiency of 100%. (**D**) Sequence chromatograms of three plasmids sequenced from engineering the Y189A variant of KAT2B with SuperFi II polymerase. Two carried the correct mutation and the third one harbored an A insertion at the primer site, likely due to primer impurities, at an efficiency of 66.7%.

From the earlier work on the identification and cloning of the cDNA for KAT2B [24], we noticed that the coding sequence for the N-terminal 90 amino acid residues is extremely GC-rich, reaching 95–100% in some areas (Figure 2A, Figures S1C and S2A). This region was extremely difficult to sequence manually with T7 DNA polymerase [24], suggesting that this region acts as a barrier hindering DNA synthesis in vitro. Thus, we hypothesized that this extremely GC-rich region causes insufficient denaturation and interferes with PCR amplification. As a result, we decided to modify PCR parameters to improve plasmid denaturation before and during PCR amplification. Prior to PCR, the KAT2B expression plasmid was denatured at 105 °C for 5 min and transferred immediately onto ice. Supercoiled plasmid DNA is hard to denature by heating at 96 °C for 3 min, so we decided to raise the temperature and prolong the incubation time. The denatured plasmid was subsequently used for PCR with SuperFi II or Q5 DNA polymerase. During PCR, the denaturation temperature was raised to 98 °C. By comparison, the initial denaturation temperature was at 96 °C and the subsequent denaturation temperature during the PCR cycles was reduced to 93 °C for P3a mutagenesis [10]. With these two modifications, we carried out mutagenesis with SuperFi II or Q5 DNA polymerase. Strikingly, we obtained a good number of colonies, although the colony count from SuperFi II DNA polymerase was higher than that from Q5 DNA polymerase.

We next investigated whether the colonies harbored plasmids with expected mutations. For each mutagenesis reaction, plasmids from three to four colonies were isolated for Sanger sequencing. For the SuperFi II polymerase condition, we analyzed 39 plasmids for engineering 10 point mutants and all but one carried the correct mutations, yielding the almost ideal efficiency of 97.5% (Figure 2B–D). For the Q5 polymerase condition, we analyzed 13 plasmids for engineering four point mutants and only five plasmids harbored the correct mutations. The remaining eight plasmids were wild-type, resulting in an average efficiency of 38.5% (Figure 2B). These results indicate that P3a mutagenesis mediated by SuperFi II but not Q5 DNA polymerase is highly efficient towards the heat-denatured KAT2B plasmid. Thus, we concluded that SuperFi II DNA polymerase is required for efficient mutagenesis of the plasmid encoding KAT2B. Compared to P3a mutagenesis, this improved method, referred to as P3b mutagenesis [10], has two major differences: (1) an extra denaturation step prior to PCR and (2) higher denaturation temperature during PCR. In addition, SuperFi II but not Q5 DNA polymerase could be used. In comparison, both enzymes are effective for P3a mutagenesis [8].

We also examined the possibility of substituting the pre-PCR heat denaturation step with alkali denaturation, which is known to denature supercoiled plasmid DNA efficiently. For this, the alkali-denatured plasmid was used for engineering the G48C mutant via P3b mutagenesis with SuperFi II polymerase. In the same experiment, we also tested P3 mutagenesis with PfuUltra DNA polymerase as previously described [8]. Compared to P3 mutagenesis with PfuUltra DNA polymerase, P3b mutagenesis with SuperFi II polymerase yielded ~10 times more bacterial colonies (200–300 from transformation of 3 µL of the DpnI-digested PCR mixture). For each of these two conditions, plasmids from five colonies were sequenced. As previously reported [8], the plasmids from P3b mutagenesis all harbored the correct mutation, whereas those from P3 mutagenesis were all wild-type, supporting that the alkali-denatured plasmid is also a suitable DNA template for P3b mutagenesis. The results support the superiority of P3b mutagenesis over the original P3 method, which relies on PfuUltra DNA polymerase [8].

We next tested whether P3b mutagenesis is effective for introducing deletions. For this, we used the method to delete the extremely GC-rich region, as the resulting plasmid would help investigate whether this region (Figure S2A) is really the culprit underlying its incompatibility with P3 and P3a mutagenesis. We designed a pair of primers as depicted in Figure S9A,B. We carried out P3b mutagenesis with SuperFi II DNA polymerase and analyzed plasmids from 12 colonies by double digestion with EcoRI and PstI. Six plasmids yielded the expected digestion pattern for the deletion compared to the full-length plasmid. The remaining six produced the same digestion pattern as the plasmid for the full-length KAT2B. The six deletion mutant candidates were sequenced, and the results indicated that they were all correct (Figure S9C), leading to an efficiency of 6/12 (50%). These results indicate that P3b mutagenesis is also efficient for engineering deletion mutants of KAT2B.

### 3.4. P3b Mutagenesis for Engineering Different HAT Mutants

The dramatic impact of the extra heat-denaturation step on mutagenesis of the KAT2B expression plasmid raises the question whether this step is also beneficial for P3a mutagenesis with plasmids with regular GC-contents. To investigate this, we engineered four KAT6A mutants (Figure S10A). Prior to PCR, the KAT6A expression plasmid was heat-denatured at 105 °C. SuperFi II polymerase was used for the mutagenesis reactions. We analyzed three plasmids from each mutagenesis reaction. Among the 12 plasmids sequenced, none were wild-type, one K604R candidate had an additional substitution (G497V) and two S670P candidates contained an approximately 1.7 kb deletion (Figure S10C), resulting in the efficiency of 9/12 (75%, Figure S10A). Therefore, compared to the mutagenesis conditions without the heat-denaturation step [10], the efficiency with this step was actually lower for the KAT6A expression plasmid. For the mutant S670P, this step was somewhat detrimental and introduced an unwanted deletion not observed previously with P3a mutagenesis itself [10]. We then tested P3a mutagenesis for engineering eight p300 and CBP point mutants with only four pairs of primers (Figure S10B). Prior to PCR, the expression plasmids were heat-denatured at 105 °C. SuperFi II polymerase was used for PCR. We analyzed three plasmids per mutagenesis reaction. Among the 12 plasmids sequenced, three were wild-type and two failed to be sequenced, leading to the efficiency of 7/12 (58.3%, Figure S10B), although for the D1399Y and D1399H mutants, the efficiency was 100% (Figure S10D). Overall, compared to P3a mutagenesis [10], the efficiency was slightly lower. Therefore, for engineering KAT6A, p300 and CBP point mutants, P3b mutagenesis did not appear to be superior to P3a mutagenesis.

### 3.5. P3a and P3b Mutagenesis Methods to Generate SARS-CoV-2 Spike Mutants at Multiple Sites

In addition to single-site mutations, we have tested P3a site-directed mutagenesis for double-site mutagenesis, but the efficiency was reduced substantially [10]. This raises the question of how to adapt the method for multisite mutagenesis. Conceptually, different pairs of primers to engineer mutations at multiple sites could be mixed in the same PCR reaction to introduce the desired mutations. However, the preferential amplification of PCR products generated between two different primer pairs tends to dominate over the products from the entire plasmid, thereby resulting in reduced mutagenesis efficiency or even complete failure. One potential solution is to carry out mutagenesis reactions sequentially, but it is time-consuming. For example, if it takes one week to engineer one mutation (Figure S11A), it will take 5 weeks to produce a mutant with five different mutations. To circumvent this problem, we considered an alternative strategy in which mutagenesis cycles are repeated sequentially without colony isolation and plasmid sequencing until the last cycle (Figure S11B). This way, it takes about a week to engineer one mutant with five mutations.

Mathematically, this strategy relies on the assumption that the mutagenesis efficiency for each cycle is at or higher than 90%. In this case, the minimal theoretical efficiency to achieve the mutant with all five mutations after five different cycles of mutagenesis reactions is 59% (by multiplying 0.9 × 0.9 × 0.9 × 0.9 × 0.9). Moreover, if the mutagenesis efficiency for each cycle is 80% or 75%, the theoretical efficiency to achieve the mutant with all five mutations quickly drops to 32.8% or 23.7%, respectively. If the mutagenesis efficiency per cycle is 50% (such as P3 mutagenesis [8]), the theoretical efficiency to achieve the mutant with all five mutations is barely 3%, thereby making it an almost impossible task. Thus, in theory, this strategy (Figure S11B) works effectively with P3a or P3b but not P3 mutagenesis.

To assess the strategy (Figure S11B) experimentally, we sought to engineer SARS-CoV-2 spike mutants containing R346T, F456L (or L455S_F456L), Q493E and V1104L (Figure S11C), which are present in derivatives of JN.1 subvariant [33,34]. For the F456L and L455S_F456L substitutions, a single pair of primers was used as described above for Figure S7B. Two sets of mutagenesis reactions were carried out in parallel, with the expression plasmid for the spike protein of the D416G or Omicron variant as the template. For the expression plasmid for the spike protein of the D416G variant, P3b mutagenesis was carried out without the extra denaturation step prior to PCR (P3b*, Figure S11D), whereas for the expression plasmid for the spike protein of the Omicron variant, P3b mutagenesis with the extra denaturation step before PCR was performed. As the coding sequences for the two variants are almost identical (except for some mutations), results from this experiment could also shed light on the importance of the extra denaturation step prior to PCR. For the expression plasmid for the spike protein of the D416G variant, a fifth mutation (L981F) was engineered; this mutation is already present in the expression plasmid for the spike protein of the Omicron variant [33,34]. We repeated the mutagenesis cycles four (Omicron) or five (D614G) times with 4 or 5 different pairs of primers. In both cases, SuperFi II DNA polymerase was used.

For the first three (Omicron) or four (D614G) mutagenesis cycles, bacteria transformed from a mutagenesis reaction were grown in one tube for miniprep of the plasmid mixture, which was then used as a template for the subsequent mutagenesis reaction (Figure S11B). This cycle was repeated three (Omicron) or four (D614G) times. For the last cycle, bacteria were plated out on an agar plate for colony isolation, plasmid preparation and Sanger sequencing. To save time and reduce sequencing costs, only one sequencing primer (V320-F, Figure S11C) was used to identify the first three mutations R346T, F456L (or L455S_F456L) and Q493E. Similarly, the sequencing primer Q965-F was used to identify the mutations L981F and V1104L (Figure S11C). As shown in Figure S11D (middle), P3b mutagenesis without the extra denaturation step before PCR yielded six correct mutant plasmids out of 10 sequenced, resulting in an efficiency of 60%. These six correct plasmids carry all five mutations desired to engineer: R346T, F456L (or L455S_F456L), Q493E, L981F and V1104L. As shown in Figure S11D (right), P3b mutagenesis with the extra denaturation step yielded two correct mutants out of four plasmids sequenced. The two correct plasmids carry all four or five mutations designed to engineer: R346T, F456L (or L455S_F456L), Q493E and V1104L. Shown in Figure S11E–H are representative sequencing chromatograms. Thus, the strategy depicted in Figure S11B is effective for be adopted for P3b mutagenesis, with or without the extra denaturation step prior to PCR, to engineer SARS-CoV-2 spike mutations at multiple sites.

### 3.6. Two GC-Rich Fragments Render the KAT2B Plasmid Incompatible with P3a Mutagenesis

**Figure 3:**
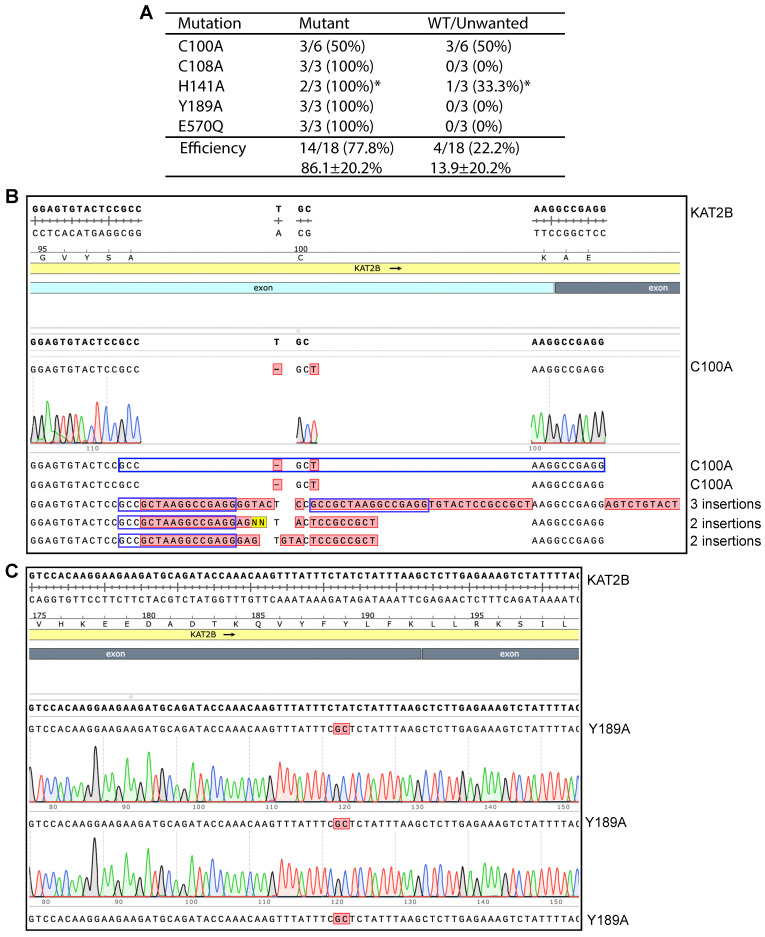
Impact of pre-PCR denaturation on engineering KAT2B mutants. (**A**) Efficiency of P3b mutagenesis with SuperFi II DNA polymerase. Different from what is depicted in Figure 2, the KAT2B expression plasmid was not heat-denatured at 105 °C prior to PCR. Per mutation, three or six plasmids were analyzed. Among 18 plasmids analyzed for the five mutations, all but four were the correct mutants. Three C100A candidates possessed two insertions (see panel C). The asterisk denotes that one plasmid showed an abnormal digestion pattern (Figure S8F, lane 10), even though the H141A mutation was present in the plasmid. (**B**) Sequence chromatograms of six plasmids analyzed for engineering the C100A mutant. Three were correct and the rest contained 2–3 insertions at the primer site, leading to an efficiency of 50%. The third insertion in candidate #4 is 52 nucleotides long, with the following sequence: 5′-AGTCTGTACT CCGCCGCTAA GGCCGAGGAG TACTCCGCCG CTAAGGCCGA GG-3′. (**C**) Sequence chromatograms of three plasmids analyzed from engineering the Y189A variant. All three carried the correct mutation, resulting in an ideal efficiency of 100%.

We next investigated the mechanisms whereby P3b but not P3a mutagenesis is efficient for engineering KAT2B mutants. P3b mutagenesis is different from the P3a method in two aspects: (1) heat-denaturation of the plasmid template at 105 °C for 5 min (or alkali denaturation) before PCR amplification; and (2) higher denaturation temperature during PCR, being 98 °C instead of 96 °C for the initial denaturation step and 93 °C for each PCR cycle. To investigate whether both modifications are required, we took an elimination strategy by removing each modification from the method. For this, we first tested P3b mutagenesis without the extra denaturation step prior to PCR to engineer five KAT2B mutants (Figure 3A, P3b*). As shown in Figure 3A,C, the method reached the ideal efficiency for engineering four mutants: C108A, H141A, Y189A and E570Q, indicating that the extra denaturation step is dispensable for engineering these four mutants. For C100A, it was problematic: among six candidates analyzed, three were correct and three contained 2–3 insertions at the primer site (Figure 3B), indicating that the conditions were not optimal. Thus, the requirement for the extra denaturation step prior to PCR is dependent on the mutations to be engineered.

The next question was how important it is to use 98 °C instead of 96 °C for the initial denaturation step and 93 °C for PCR cycles. To address this question, we reduced 98 °C to 96 °C for the initial denaturation step and to 93 °C for each PCR cycle but kept the denaturation step prior to PCR. We used the modified method to engineer the C100A, C108A, H141A and Y189A mutants. The mutagenesis reactions yielded no or few colonies, so the experiment was terminated. These results indicate that it is crucial to use 98 °C for the initial denaturation step and during PCR cycles.

**Figure 4:**
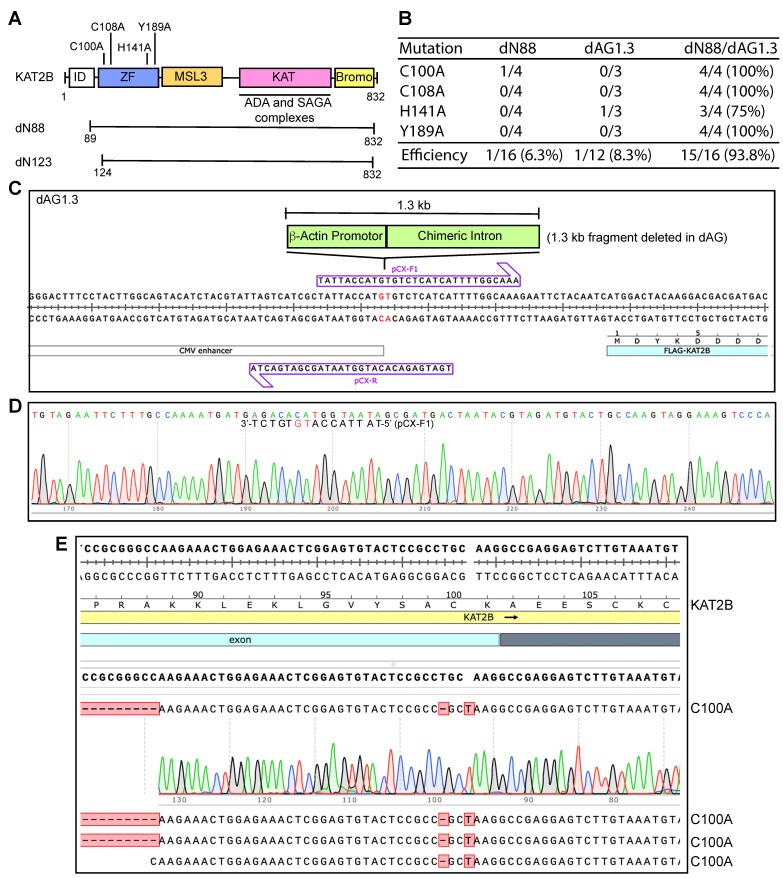
Efficiency of P3a site-specific mutagenesis to engineer KAT2B mutants. (**A**) Illustrated are four point mutations located within the zinc finger (C100A, C108A, H141A and Y189A) of KAT2B. (**B**) Efficiency of P3a mutagenesis to engineer four KAT2B mutants when using the dN88, dAG1.3 and dN88/dAG1.3 plasmids as templates. We analyzed 3–4 bacterial colonies per mutagenesis reaction. For the dN88 template, only one out of the 16 plasmids sequenced was correct, corresponding to a C100A mutant. For the dAG1.3 template, one out of 12 plasmids possessed the correct mutation (H141A). For the double deletion template dN88/dAG1.3, 15 out of 16 plasmids sequenced were correct, resulting in an efficiency of 93.8%. (**C**) Two primers designed to delete the 1.3 kb fragment encompassing β-actin promoter and a chimeric intro on the CAG promoter, with the resulting plasmid named dAG1.3 (panel B). (**D**) Sequence chromatogram of a correct plasmid from P3b mutagenesis using primers pCX-F and pCX-R to engineer dAG1.3. Notably, pCX-F contains an error, but this pair of primers still yielded one correct plasmid out of 18 analyzed. This error was corrected in primer pCX-F1 and the resulting mutagenesis efficiency was 6/6 (100% here) when the primers pCX-F1 and pCX-R were used. (**E**) Sequence chromatograms of four plasmids sequenced from engineering the C100A mutation on the dN88/dAG1.3 plasmid. All four carried the correct mutation, resulting in an ideal efficiency of 100%.

We had become aware of the extremely GC-rich 0.27 kb fragment that encodes the N-terminal part of KAT2B (Figures S1C and S2A) when the cDNA was cloned and manually sequenced many years earlier [24], so our initial hypothesis was that this fragment renders the expression plasmid incompatible with P3a mutagenesis. To test the hypothesis, we utilized the expression plasmid with this region deleted via P3b cassette mutagenesis (Figure 4A and  Figure S9). This plasmid was then subjected to P3a mutagenesis for engineering the C100A, C108A, H141A and Y189A mutants. As shown in Figure S8D,E, a majority of the candidates carried deletions, so the dN88 plasmid remained incompatible with P3a mutagenesis. By contrast, this plasmid was compatible with P3b mutagenesis (Figure S8F). As the C100A mutation was especially problematic for P3b mutagenesis without the extra denaturation step prior to PCR (Figure 3A,B), the presence of the region around the coding sequence for Cys100 may render the plasmid incompatible with P3a mutagenesis. We thus engineered a plasmid with a larger deletion (e.g., dN123) via P3b mutagenesis (Figure 4A). However, the plasmid was still incompatible with P3a mutagenesis (Figure S12A). These unexpected results suggested two possibilities: (1) this GC-rich region is not behind incompatibility of the plasmid with P3a mutagenesis; and (2) one or more GC-rich regions on the vector backbone also contribute to the incompatibility.

To delineate this, we inspected the sequence of the vector backbone for the presence of GC-rich domains and noticed that the synthetic CAG promoter upstream from the KAT2B coding sequence possesses a 1.3 kb extremely GC-rich fragment [25]. This fragment is composed of the promoter, the first exon and the first intron of the chicken β-actin gene, and the splice acceptor of the rabbit β-globin gene [25]. Sanger sequencing from the upstream CMV promoter led to premature chain termination after a stretch of 27 guanines in the 1.3 kb fragment (Figure S13A,B). This difficulty is quite reminiscent of the sequencing problems that we encountered when manually sequencing the GC-rich region that encodes for the N-terminal 90 residues of KAT2B [24]. These observations suggest that the 1.3 kb GC-rich fragment of the expression vector also poses serious challenges for DNA synthesis in vitro.

To investigate whether the 1.3 kb fragment renders the plasmid incompatible with P3a mutagenesis, we engineered the expression plasmid dAG1.3 (Figure 4C,D), in which the 1.3 kb fragment encompassing the β-actin promoter and a chimeric intron of the CAG promoter [24,25] was deleted. This plasmid was then used for P3a mutagenesis. As shown in Figure 4B and Figure S12B, the region encompassing the CAG promoter appeared to be largely intact, but two 0.2- and 0.23 kb fragments from EcoRI/PstI double digestion of the resulting plasmids were frequently missing. These two fragments encompass the 0.27 kb GC-rich region encoding the N-terminal part of KAT2B, so we reasoned that its presence is still problematic. As a result, we utilized P3b mutagenesis to engineer a double deletion mutant plasmid, dN88/dAG1.3, in which both GC-rich regions were deleted. This plasmid was then used for P3a mutagenesis and representative colonies were inoculated for plasmid miniprep and restriction digestion. As shown in Figure S12C,D, no deletions were detected among 16 plasmids analyzed by restriction digestion. Sequence analysis revealed that the plasmid dN88/dAG1.3 was mutated at an average efficiency of 93.8% (Figure 4B,E). Together with the findings on the dN88 and dAG1.3 plasmids (Figure 4B), these intriguing results indicate that the two GC-rich regions make the KAT2B plasmid problematic for P3a mutagenesis. These results also support that either of the two regions is sufficient for rendering the plasmid incompatible with P3a mutagenesis. Notably, one of the two GC-rich regions is located within the CAG promoter, which is frequently used for expressing proteins in mammalian cells. The other GC-rich region encodes an intrinsically disordered domain conserved from *Drosophila* to humans (Figure S2B). There are many proteins containing such domains, e.g., CDK13 as described below.

Notably, guanine-rich sequences tend to form G-quadruplexes [11,38] and have recently been shown to stall DNA replication [12]. Using the consensus sequence dG_3+_N_1-7_G_3+_N_1-7_G_3+_N_1-7_G_3+_, where the letter d refers to DNA (i.e., deoxy-) and N is any nucleotide base (including guanine), G-quadruplex-forming sequences were found to be widely spread in the human genome [38,39]. Such sequences are important for regulating different DNA-based processes [11]. Our results showing the DNA synthesis stalling at a stretch of guanines support that G-quadruplexes impede P3a site-directed mutagenesis in vitro (Figure S13B,C). As G-rich sequences such as telomeric repeats tend to form such quadruplexes [38,39], it is likely that the GC-rich sequences in the KAT2B expression vector stalls PCR-based DNA synthesis, an essential step in P3a site-directed mutagenesis. Thus, G-quadruplex formation is one potential mechanism underlying the challenges that PCR-based mutagenesis in vitro faces.

### 3.7. P3b Mutagenesis of sgRNA/Cas9 Expression Plasmids with the CAG Promoter

To determine the general applicability of the above findings with the KAT2B expression vector, we investigated whether similar mutagenesis problems occur with other plasmids containing GC-rich sequences. For this, we first sought to analyze other plasmids containing the CAG promoter, which is one of the strongest promoters driving gene expression in mammalian cells [25]. Among ~51,000 plasmids available from Addgene in May 2025, 310 possess explicit entries with the keywords ‘CAG promoter’. In addition, many other plasmids contain the promoter but do not have these two keywords in their records. When we were analyzing four sgRNA/Cas9 expression plasmids from Addgene, including pX330 spCas9-mSA (Cat. No. 113096), eSpCas9 (1.1) _No_FLAG_ATP1A1_G3 (Cat. No. 86611), pX459V2.0-HypaCas9 (Cat. No. 108294), and pCAG-hMb3Cas12a-NLS (nucleoplasmin)-3xHA (Cat. No. 115142), we noticed that they all contain the CAG promoter. Among ~51,000 plasmids available from Addgene, 870 possess explicit entries with the keyword ‘pX330’ and 263 contain the keyword ‘pX459’ (based on data obtained in May 2025). This is because pX330 and pX459, expressing Cas9 and sgRNA scaffolds, were initially described in the first papers on CRISPR [40,41]. Thus, together with the 310 plasmids with the keywords ‘CAG promoter’, there are at least 310 + 870 + 263 = 1443 CAG promoter-containing plasmids (corresponding to ~2%) in Addgene. An interesting question is whether P3b sited-directed mutagenesis is required for engineering mutations on such plasmids.

**Figure 5:**
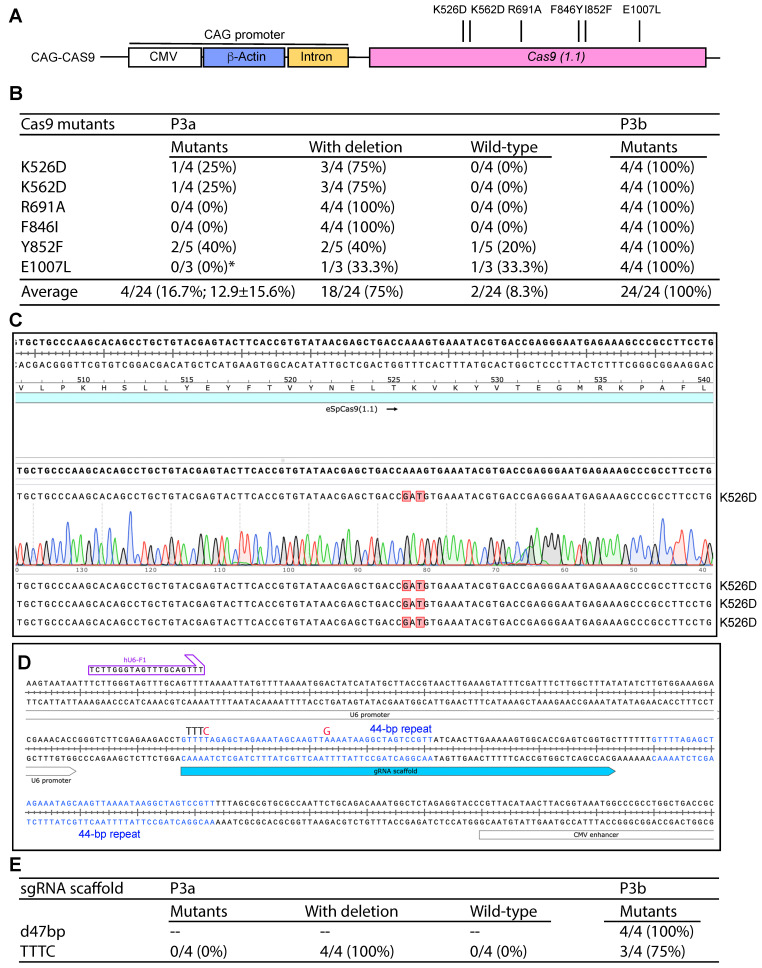
Efficiency of P3a site-specific mutagenesis to engineer Cas9 and sgRNA scaffold mutants. (**A**) Cartoon illustration of the CAG promoter upstream from the coding sequence of Cas9. Six point mutants to be engineered are also shown. (**B**) Efficiency of P3a and P3b mutagenesis to engineer six Cas9 mutants when using a CAG-Cas9 plasmid as the template. We analyzed 3–5 bacterial colonies per mutagenesis reaction. The asterisk denotes that for the E1007L mutagenesis reaction by P3a mutagenesis, only three colonies were obtained for the experiment. (**C**) A representative sequence chromatogram of one correct plasmid and the sequences of three such plasmids are shown. (**D**) The Cas9 mammalian expression vectors pX330, pX459 and some of their derivatives possess two 44 bp repeats in the sgRNA scaffold and its downstream region. The second repeat is problematic to PCR primers designed to mutate the scaffold or insert sgRNA-coding sequences just upstream from the scaffold [10]. (**E**) Efficiency of P3a and P3b mutagenesis to engineer a sgRNA scaffold point mutant (TTTC) and a 47 bp deletion just downstream from the scaffold (d47bp). The d47bp mutation removes the second 44 bp repeat (see panel D) along with 3Ts downstream from the repeat. The TTTC carries two point mutations: the first one replaces the fourth T in the TTTT sequence with C and the second point mutation switches a base-pairing A to G. As the TTTT sequence serves a premature transcription termination signal for RNA polymerase III-dependent transcription from the U6 promoter, these two point mutations are known to enhance the transcription of sgRNA scaffold [10].

As a proof-of-principle, we initially tested one of them [eSpCas9 (1.1) _No_FLAG_ATP1A1_G3] (Figure S14A) [42]. As shown in Figure S14B, the sequencing reaction from the CAG-R primer downstream from the β-actin promoter was able to read the C-stretch corresponding to the G-stretch on the reverse strand that stalled the reaction from the primer CMV-F1 (Figure S13B), supporting that G- but not C-stretches block DNA synthesis. This is consistent with the notion that G-stretches form G-quadruplexes and stall DNA replication [12]. We initially tested the plasmid to engineer six Cas9 mutations (K526D, K562D, R691A, F846Y, I852F and E1007L) with P3a mutagenesis (Figure 5A). These mutations are known to improve the fidelity and activity of Cas9 [43,44,45,46], in line with the goal to obtain optimal Cas9 variants for clinical genome editing. For each mutagenesis reaction, 3–5 colonies were analyzed and most of them possessed unexpected deletions, leading to an average mutagenesis efficiency of 16.7% (Figure 5B). Among the six mutants, no mutants were obtained for three of them (R691A, F846I and E1007L). This efficiency was very poor. Consistent with what we observed with the KAT2B expression vector, deletion was the main problem. Then we tested the Cas9 plasmid to engineer the same six mutants with P3b mutagenesis (Figure 5A). For each mutagenesis reaction, four colonies were analyzed and all plasmids from the 24 colonies analyzed possessed expected mutations, resulting in the ideal efficiency of 100% (Figure 5B,C). These results indicate that P3b but not P3a mutagenesis worked efficiently with the Cas9 plasmid. As sequence inspection revealed that the CAG promoter is the only region that possesses GC-rich sequences, the results also support that the CAG promoter renders the plasmid incompatible with P3a mutagenesis.

During the process, we noticed two 44 bp repeats shared by the sgRNA scaffold and its downstream region (Figure 5D). Since the second repeat hinders P3a mutagenesis of the scaffold and the insertion of an gRNA-coding sequence upstream from the scaffold, we utilized P3b mutagenesis to delete the second repeat along with its downstream TTT, leading to removal of a 47 bp fragment. From the mutagenesis reaction, plasmids from four colonies were analyzed and all of them possessed the expected deletion, leading to the ideal efficiency of 100% (Figure 5E). We also tested this with two other pX330 or pX459-derived vectors and similar results were obtained. Optimization of sgRNA is important for enhancing the editing efficiency. One possibility is to replace the premature termination signal TTTT within the scaffold with TTTC [47]. Accordingly, the downstream AAAA needs to be replaced with GAAA [47]. We carried out P3a and P3b mutagenesis reactions and analyzed four colonies from each method. As shown in Figure 5E, P3a mutagenesis resulted in only unwanted deletions, whereas P3b mutagenesis led to a mutagenesis efficiency of 3/4 (75%). Thus, P3b mutagenesis is highly efficient for modifying sgRNA/Cas9 expression vectors that contain the CAG promoter.

### 3.8. P3b Mutagenesis of a CDK13 Expression Plasmid with Highly GC-Rich Sequences

**Figure 6:**
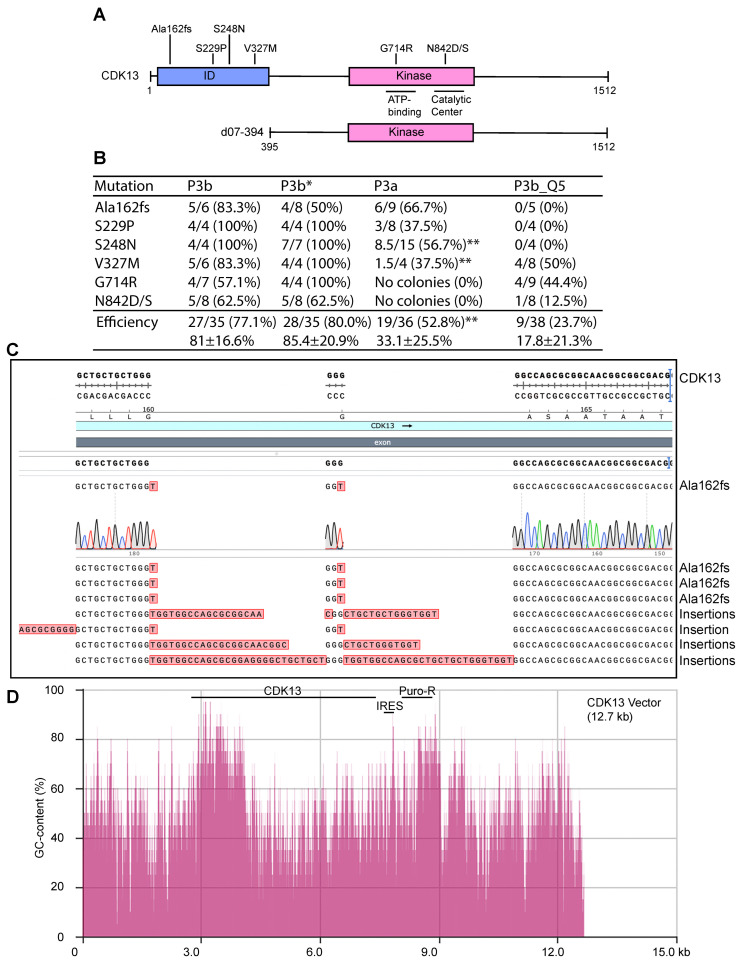
P3a and P3b mutagenesis for engineering various CDK13 mutants. (**A**) Domain organization of CDK13, shown with seven point mutants identified in patients with cancer or a developmental disorder [13,14,48]. d07-394, a deletion mutant with the ID domain removed, is also shown. ID, intrinsically disordered domain. (**B**) Efficiency of P3a and P3b mutagenesis in generating seven different CDK13 mutants. For P3b or P3b* mutagenesis, 4–8 colonies were sequenced per reaction and the number of mutant colonies are listed here, along with mutagenesis efficiency (%) in brackets. P3b* refers to P3b mutagenesis without the extra denaturation step prior to PCR amplification. P3a and P3b_Q5 mutagenesis reactions yielded no or few colonies, so mutagenesis reactions needed to be repeated to obtain sufficient colonies for analysis. For P3a mutagenesis, 4–15 colonies were sequenced per mutation if available, from four different reactions. Notably, from P3a mutagenesis, no colonies were obtained from four different mutagenesis reactions to engineer the G714R and N842D/S mutants. P3b_Q5 denotes P3b mutagenesis with Q5 DNA polymerase used for PCR. For P3b_Q5 mutagenesis, 4–9 colonies were analyzed per mutation, from two different reactions. A mixed clone between the wild-type and mutant is counted as 0.5 as denoted by the double asterisk (**). (**C**) Representative sequence results of eight plasmids from P3b mutagenesis without the extra denaturation step prior to PCR amplification. (**D**) Distribution of the GC-content along the entire CDK13 expression vector. The sequence from the Addgene website for this plasmid was copied and pasted into the calculation box provided by the NovoPro website. The GC-content was calculated with a window size of 20. The vector is 12.7 kb and the CDK coding sequence corresponds to nucleotides from 2910 to 7448. A GC-content display tool within the SnapGene package was also used to assess the GC-rich regions. IRES, internal ribosome entry site; Puro-R, puromycin-resistant marker.

**Figure 7:**
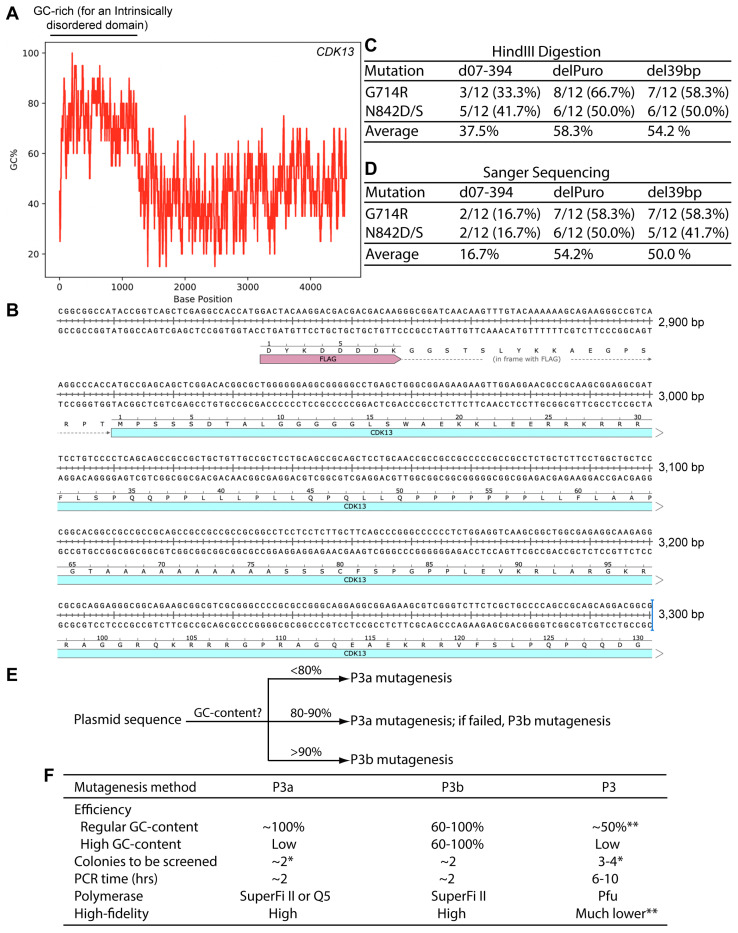
The coding sequence for human CDK13 harbors an extremely GC-rich region. (**A**) Distribution of the GC-content along the coding sequence of human CDK13 expression. The human CDK13 coding sequence was copied and pasted into the calculation box provided by the VectorBuilder website. The GC-content was calculated with a window size of 20. (**B**) Sequence of the extremely GC-rich region of the human CDK13 coding sequence. Only a portion of the region is shown here. (**C**) For each mutagenesis reaction, plasmids from 12 bacterial colonies were digested with HindIII digestion, with counts of plasmids with the expected digestion patterns shown here. The remaining plasmids showed deletions. (**D**) Plasmids with the expected digestion patterns, as shown in panel D, were sequenced and counts of those with the expected mutations are shown here. The remaining ones were wild-type. The total counts of plasmids refer to those initially analyzed by restriction digestion. (**E**) An operational model on selecting P3a or P3b mutagenesis for a given plasmid. The percentage values refer to local regions rather than the entire plasmids, are based on our limited experience with ~20 expression plasmids and should only be used as an approximate guide. For P3a mutagenesis, either Q5 or SuperFi II polymerase can used [10], but for P3b mutagenesis, SuperFi II polymerase is preferred (Figure 2B and Figure 6B). In terms of costs, Q5 polymerase is less expensive than SuperFi II polymerase. However, the costs for generating mutants are mainly from primer synthesis and plasmid sequencing, instead of the polymerases used for mutagenesis reactions. For P3b mutagenesis, the requirement for the extra denaturation step is dependent on mutations to be engineered. For some of those near or within a GC-rich region, such as C100A of KAT2B (Figure 3A) and Ala162fs of CDK13 (Figure 6B), this step is required for high efficiency. (**F**) Summary of different parameters for comparison of the P3, P3a and P3b mutagenesis methods. The P3a and P3b methods are much more efficient and faster than P3 mutagenesis [8,10]. Moreover, the risk of introducing unwanted mutation is also much lower with the P3a and P3b methods. The single asterisk (*) denotes the colony number for analysis when plasmids with regular GC-rich content are used for mutagenesis. The double asterisks (**) refers to the notion that P3a but not P3 mutagenesis worked efficiently for engineering the R15W mutant of BRPF3 (Figure 1) [8]. Moreover, P3 mutagenesis frequently introduced unwanted insertions and deletions at the primer sites (Figure S3). As for plasmids with extremely GC-rich sequences, it is almost impossible for P3/P3a mutagenesis or many colonies needs to be analyzed, as shown herein for KAT2B.

To assess the general applicability of the conclusion drawn above from analysis of the KAT2B expression plasmid, we also tested expression plasmids for other proteins possessing intrinsically disordered domains encoded by GC-rich sequences. For this, we chose CDK13 as it carries an intrinsically disordered domain encoded by a highly GC-rich fragment (Figure 6A and Figure 7A,B). Moreover, this 12.7 kb plasmid is much larger than that for KAT2B (~7.0 kb). Germline mutations of the CDK13 gene cause a neurodevelopmental disorder with developmental delay and intellectual disability [14,48], whereas somatic mutations are linked to carcinogenesis [13]. A rapid method to engineer the corresponding mutants helps delineate the pathogenicity of the mutations rapidly and economically. As a proof-of-principle experiment, we utilized P3b mutagenesis to engineer seven point mutants: Ala162fs, S229P, S248N, V327M, G714R, N842D and N842S, all of which were recovered from patients with cancer or the neurodevelopmental disorder [13,14,48]. As the latter two mutations alter the same residue (N842), a single primer pair was used. As a result, six pairs of primers were designed to engineer the seven mutations. We utilized P3b mutagenesis with or without the extra denaturation step prior to PCR amplification. Both conditions yielded decent amounts of colonies and some of them were inoculated for plasmid miniprep and Sanger sequencing. As shown in Figure 6B, the average efficiency was 77.1% for P3b mutagenesis and 80.0% for the same condition but without the extra denaturation step before PCR (P3b*). Notably, the latter was problematic for Ala162fs, because 4 out of 8 colonies analyzed possessed insertions at the primer site (Figure 6B,C). Thus, P3b mutagenesis is slightly superior to the same method without the extra denaturation step prior to PCR.

For comparison, we also carried out similar P3a mutagenesis reactions with SuperFi II DNA polymerase. The reactions resulted in no or few colonies. For example, no colonies could be obtained for four different sets of G714R and N842D/S mutagenesis reactions, so P3a mutagenesis failed to engineer these three mutants. For the remaining four mutants, inconsistent numbers of colonies were obtained from four different sets of mutagenesis reactions. HindIII restriction digestion detected deletions in 40–50% of the resulting plasmids. The ones with expected digestion patterns were sequenced to detect the mutations. For the four mutations, we obtained 19 mutants out of 36 analyzed by digestion and Sanger sequencing, with the average mutagenesis efficiency of 52.8% (Figure 6B). Along with the failures from the G714R and N842D/S, this result indicates that P3a mutagenesis is much less efficient and reliable than the P3b method with the CDK13 expression plasmid.

In addition to SuperFi II DNA polymerase, we utilized Q5 DNA polymerase for P3b mutagenesis of the CDK13 expression plasmid. The six pairs of primers described above were used to engineer the seven mutations, with the PCR conditions identical to those used with SuperFi II DNA polymerase. But the mutagenesis reactions yielded no or few colonies. HindIII restriction digestion of plasmids from some representative colonies revealed frequent deletions. The ones with expected digestion patterns were sequenced. As shown in Figure 6B (right), only nine plasmids out of 38 carried expected mutations, with an average efficiency of 23.7%. Notably, no mutant plasmids were obtained for the Ala162fs, S229P and S248N mutations. These findings indicate that for P3b mutagenesis with the CDK13 expression plasmid, Q5 DNA polymerase performed poorly. Thus, with this plasmid, SuperFi II DNA polymerase is superior to Q5 polymerase, which aligns well with the findings described above for the plasmid expressing KAT2B (Figure 2B), further attesting to the importance of DNA polymerases used for PCR in P3b mutagenesis. These results also suggest that although both are considered to be of high-fidelity, these two thermostable DNA polymerases exhibit different fidelity during in vitro synthesis when GC-rich plasmids are used as templates. Notably, both polymerases are effective with the BRPF3 expression plasmid (Figure 1 and Figure 3B) and many other plasmids that possess regular GC-contents [8]. Thus, performance of these two polymerases is dependent on the templates used for mutagenesis.

### 3.9. GC-Rich Sequences Make the CDK13 Plasmid Incompatible with P3a Mutagenesis

To delineate whether the ID-coding GC-rich region is indeed responsible for making the plasmid incompatible with P3a mutagenesis, we utilized P3b cassette mutagenesis to delete this region and engineer the expression plasmid for the deletion mutant, d07-394 (Figure 6A). Plasmids from a dozen colonies were analyzed by HindIII digestion and eight showed the correct digestion patterns, at an efficiency of 8/12 (66.7%). Two of them were sequenced and found to carry the correct deletion. One of them was used as the template for engineering the G714R and N842D/S mutations via P3a mutagenesis. As a single pair of primers was used for the N842D/S mutations, two mutagenesis reactions were carried out to engineer the three mutations. Bacterial colonies were easily obtained. This is in stark contrast to the wild-type CDK13 expression plasmid, for which no colonies could be obtained when the P3a protocol was used, suggesting that the deletion is beneficial.

For each reaction, plasmids from a dozen bacterial colonies were initially analyzed by HindIII restriction digestion. For the G714R and N842D/S mutagenesis reactions, three and five plasmids showed the expected digestion patterns, respectively. The eight plasmids were sequenced, with two of them carrying the G714R mutation, two harboring the N842D mutation and the rest being wild-type. Thus, the efficiency was 16.7% (2/12) for the G714R or N842D/S mutagenesis reactions. This efficiency is still higher than what was observed with the wild-type CDK13 expression plasmid when P3a mutagenesis was carried out, but much lower than the same plasmid with P3b mutagenesis (Figure 6B). These results suggest that while the ID-coding GC-rich region affects the compatibility of the wild-type CDK13 expression plasmid with P3a mutagenesis, there are other DNA sequences contributing to the problem.

Two potential candidates are a 39 bp high GC-rich element just upstream from the internal ribosome entry site (IRES) and the 0.6 kb coding region of the puromycin-resistant marker (Figure 6D and  Figure S15A). To investigate whether these two DNA fragments have any roles in making the CDK13 expression plasmid incompatible with P3a mutagenesis, we deleted these two fragments by P3b mutagenesis one by one. For each mutagenesis reaction, we analyzed plasmids from six bacterial colonies by HindIII digestion. To detect the 39 bp deletion, we utilized a 1.2% agarose gel to separate the digested products and found that out of six plasmids analyzed, five showed the expected digestion pattern of the deletion mutant, with the remaining one possessing a large deletion. The five mutant candidates were further analyzed by Sanger sequencing and the results confirmed that all of them possessed the deletion of the 39 bp region (del39bp, Figure S15B). For the deletion of the 0.6 kb fragment, five out of six plasmids analyzed exhibited the expected digestion pattern of the deletion mutant, with two of the remaining three being the wild-type and one possessing a large deletion. The three mutant candidates were subject to Sanger sequencing and the results indicated that all of them carried the deletion of the 0.6 kb region (delPuro, Figure S15C). Together with the findings on the deletion mutant d07-394, these results indicate that P3b mutagenesis is efficient in introducing small or large deletions to the CDK13 expression plasmids.

The modified expression plasmids del39bp and delPuro (Figure S15) were then used to assess the impact of the 39 bp and 0.6 kb fragments, respectively, on compatibility with P3a mutagenesis. For this, the plasmids were used as templates for engineering the G714R and N842D/S mutations via P3a mutagenesis, as described above for the d07-394 expression plasmid. Just as observed with this deletion plasmid, we obtained expected amounts of bacterial colonies from all four mutagenesis reactions. For each reaction, plasmids from 12 colonies were isolated and digested with HindIII. The results revealed that 54–58% plasmids showed the expected digestion patterns, with the rest possessing deletions (Figure 7C). The plasmids with the expected digestion patterns were subject to Sanger sequencing and an absolute majority of them was found to possess the correct mutations, with the remaining ones being the wild-type. Among all colonies analyzed, 50–54% contained plasmids with the correct mutations (Figure 7D). This is much higher than the efficiency obtained with the d07-394 expression plasmid (Figure 7D). These results support that both the 39 bp GC-rich fragment at the 5′ area of the IRES sequence and the GC-rich coding sequence for the puromycin-resistant marker (Figure 6D and Figure S15) contribute to the incompatibility of the original CDK13 expression plasmid with P3a mutagenesis. This is in addition to those with GC-rich sequence encoding the ID-coding region of CDK13 (Figure 7A). Therefore, the expression plasmid for this kinase possesses three GC-rich regions affecting the incompatibility with P3a mutagenesis.

## 4. Discussion

Site-directed mutagenesis is a basic molecular biology tool, but how to reach the ideal efficiency of 100% from plasmid to plasmid is critical for using this tool effectively and economically. An innovative site-directed mutagenesis strategy involves utilizing primer pairs with 3′-overhangs [5,6]. Based on this strategy, we have recently developed P3 and P3a mutagenesis methods [8,10]. While P3 mutagenesis relies on Pfu polymerase and its derivatives, P3a mutagenesis utilizes SuperFi II and Q5 DNA polymerases, which are superior to Pfu in terms of fidelity, processivity and synthesis speed. Among all plasmids that we have tested, the BRPF3 and KAT2B expression plasmids were problematic for P3 mutagenesis [8], raising the question whether they remain difficult for P3a mutagenesis [10]. In the current study, we demonstrate that this method worked efficiently with the BRPF3 plasmid (Figure 1) but not the KAT2B plasmid. Notably, an extremely GC-rich region is present on the KAT2B expression vector (Figures S1C and S2A), with the GC-content reaching 95–100% in certain areas (Figures S1C and S2A). An extra denaturation step prior to PCR and a higher denaturation temperature during PCR were thus introduced to develop P3b mutagenesis. The expression plasmid was suitable for P3b mutagenesis mediated by SuperFi II and Q5 polymerases, but the efficiency was much higher for the former (Figure 2B). Deletion analysis revealed that this GC-rich region is sufficient for rendering the KAT2B plasmid incompatible with P3a mutagenesis (Figure 4B).

Intriguingly, the GC-rich region encodes an intrinsically disordered domain of KAT2B rich in alanine, glycine and proline residues (Figure S2B). According to the genetic codon table, the eight G/C-G/C-G/C codons encode these three residues and arginine. The intrinsically disordered domain is conserved in mouse KAT2B as well as in human and mouse KAT2A. The corresponding DNA sequences for the domains of mouse KAT2B, human and mouse KAT2A proteins are also GC-rich although the contents are not as high as in human *KAT2B.* Based on this observation, caution is needed if problems arise when P3a mutagenesis is used for engineering mutants of mouse KAT2B, human and mouse KAT2A. One common issue is the frequent occurrence of unexpected deletions, as judged by digestion analysis of the resulting plasmids when the human KAT2B vector is used as the template for P3a mutagenesis (Figure S8).

Mammalian genomes encode many proteins with intrinsically disordered domains rich in alanine, glycine, proline and arginine residues encoded by the eight G/C-G/C-G/C codons. Such domains have the propensity to be encoded by GC-rich DNA sequences. Since it is easy to verify the GC-content of a desired sequence, it is wise to analyze this before embarking on mutagenesis experiments. Related to this, we noticed that several widely known proteins, including human Tau, ERK1, AARD (alanine- and arginine-rich domain-containing protein) and CDK13 possess intrinsically disordered domains. GC-content analysis of the coding sequences of these four proteins showed that they all possess GC-rich regions. For example, the coding sequence for CDK13 has three regions with the GC-content higher than 90%, with one reaching to 100% (Figure 6D and Figure 7A,B). In such cases, it is wise to take precautions and use P3b mutagenesis if problems arise with P3a mutagenesis, such as frequent deletions as judged from plasmid digestion of the resulting KAT2B plasmids (Figures S8 and S12A,B). It is noteworthy, however, that the presence of a GC-rich region does not always imply incompatibility with P3a mutagenesis. For example, the coding sequence for human KAT8 carries an approximately 50 bp region extremely GC-rich, but P3a mutagenesis of a related expression plasmid was successful [10].

GC-rich DNA sequences not only affect transcription, replication, recombination and repair [49,50,51], but also affect mRNA export and metabolism [52]. Moreover, such sequences have also been linked to genetic diseases [53,54] and occur widely for genome regulation [51,55]. The GC-rich region of human *KAT2B* is encoded by one exon and forms a potential CpG island. Alternatively, this region may affect KAT2B mRNA export and metabolism [52]. It is unclear whether these two mechanisms are relevant to regulation of KAT2B expression. The coding regions for many other intrinsically disordered proteins are also GC-rich, but it is unknown whether this is relevant for regulation of the expression of these proteins. A related issue is that some 5′-UTR sequences are highly GC-rich (e.g., those of human BRPF1 and its paralogs, BRPF2 and BRPF3), so if such sequences are also present in the vectors to be mutated, it is important to choose P3b mutagenesis.

Serendipitously, we investigated an extremely GC-rich fragment harbored within the synthetic CAG promoter [25]. Deletion analysis revealed that this fragment was sufficient to render the KAT2B plasmid incompatible with P3a mutagenesis (Figure 4B). As this synthetic promoter is strong and frequently used for mammalian expression of different proteins, it is important to consider this factor when mutants of such proteins are to be engineered, even if their own coding sequences are not GC-rich. Furthermore, deletion analysis revealed three highly GC-rich regions (including the coding region puromycin-resistant marker) that make the CDK13 expression plasmid incompatible with P3a site-directed mutagenesis (Figure 6, Figure 7 and Figure S15). The puromycin-resistant marker and the IRES (Figure 6D) are present in many plasmids, so the P3b method should perhaps be considered for engineering mutations with such elements.

Uracil-containing DNA templates isolated from CJ236 (a *ung*^-^ and *dut*^-^ strain) significantly enhance mutagenesis efficiency with classical mutagenesis methods [28]. The underlying mechanism is that these templates can replicate efficiently in CJ236, but not in other strains (such as DH5α) lack of the *ung*^-^ and *dut*^-^ mutations [28]. We thus investigated whether uracil-containing plasmids can improve P3a mutagenesis further. The results showed that such templates did not improve the efficiency (Figure S3B), suggesting that the presence of wild-type plasmids in DpnI-digested mixtures from P3a mutagenesis is not a bottleneck that determines the efficiency of P3a mutagenesis. Together, these results indicate that the current P3a and P3b mutagenesis protocols are already close to the optimal state in terms of eliminating wild-type plasmid molecules in DpnI-digested mixtures used for transformation.

In the previous study [8] and during the course of this work (Figure 3B and Figure S3), we observed unwanted deletions or insertions at the primer sites. One possible cause for unwanted insertions at the primer sites is unfaithful chain termination when the newly synthesized strand reaches the 5′-end of an annealed primer (Figure S16A). Related to this, we noticed that the GCGAGGGCTGG sequence found in primers was recurrent in several insertions identified in multiple plasmids isolated from P3 mutagenesis with Pfu-fly (Figure S3B) [8]. By contrast, we have not found such deletions or insertions in plasmids from P3a mutagenesis reactions with SuperFi II and Q5 DNA polymerases [10]. As for unwanted deletions at the primer sites observed during engineering the R15W mutant of BRPF3, false priming at a secondary site with some sequence similarity to the primer site is a likely cause (Figure S16B). Related to this, we found recurrent 3.3 kb deletions in seven plasmids from P3 mutagenesis with Pfu-Fly and one plasmid from P3a mutagenesis with Q5U (Figure S3C). Notably, the primer site is highly GC-rich (around 85%, Figure 1B), which may contribute to non-specific priming and unwanted deletions. During engineering the S670P mutant of KAT6A, we obtained two candidates sharing the same 1725 bp deletion (Figure S10C). The two primers used were 45 nucleotides long (Figure S10C), designed at the early stage of the project when we relied heavily on instructions from a previous study [6]. These long primers perhaps allowed false priming at the 3′-end of this deletion, illustrating that longer primers are not only more expensive but also introduce complications, such as false priming and unwanted mutations. For typical P3a or P3b mutagenesis, we recommend using primers ~30 nucleotides long, which cuts costs and also raises the efficiency.

For the KAT2B, Cas9 and CDK13 expression plasmids that contain highly GC-rich regions, we observed unwanted large deletions away from the primer sites, even when P3a mutagenesis was used with SuperFi II DNA polymerase. This became worse when P3a mutagenesis was performed with Q5 DNA polymerase. Such deletions are likely due to strand jumping resulting from DNA looping induced by GC-rich sequences, in a manner similar to false priming (Figure S16B). Thus, for P3a and P3b mutagenesis, both the DNA template and the PCR conditions affect the fidelity of the polymerase being used. This also highlights the importance to use high-quality DNA polymerases for the two methods.

An important question is what factors determine the efficiency of P3a and P3b mutagenesis. Figure S16C is a cartoon illustrating how different populations of plasmids in a mutagenesis reaction dictate the eventual efficiency. According to a previous report [32], the ‘mixed’ plasmid molecules are resistant to DpnI digestion. Figure S16D presents a mathematical formula that governs the mutagenesis efficiency of P3a and P3b site-directed mutagenesis. As few wild-type clones were observed with P3a and P3b mutagenesis, the presence of wild-type plasmid molecules is not a major determinant of the efficiency. Instead, the presence of candidates with unwanted mutations is a primary factor influencing the eventual mutagenesis efficiency. This is determined by three factors: the quality of the polymerase, the difficulty level of the plasmid template and the sequence of the primer sites. Related to this, the extra heat-denaturation step is required for the KAT2B expression plasmid, but not for the others with regular GC-contents. Thus, this also explains why extra heat-denaturation did not improve the mutagenesis efficiency for the KAT6A, p300 and CBP expression plasmids (Figure S10).

In addition to engineering single-site mutations, we have developed a simple protocol for multisite mutagenesis (Figure S11). A complete P3a mutagenesis cycle takes about 4–5 days (Figure S11A). For multisite mutagenesis, inclusion of multiple primers in the same PCR reaction may not work efficiently due to dominant amplification of products between two pairs of primers [10]. As depicted in Figure S11B, the multisite mutagenesis protocol involves sequential mutagenesis reactions without colony isolation and plasmid sequencing until the last cycle. For example, if five mutations are to be engineered into a given plasmid, the mutagenesis reactions are repeated sequentially without colony isolation and plasmid sequencing for the first four cycles. For the last cycle, bacteria are plated out and 3–4 single colonies are inoculated for plasmid miniprep and sequencing. This way, it takes about a week to engineer a mutant with the five mutations. Related to multisite mutagenesis, the primer design strategy shown Figure S16F allows introduction of two or more mutations at the primer sites. It is noteworthy that the outcome from this strategy is completely different from a pair of primers with mutations at the complementary region (Figure S16E). The limitation of the strategy shown in Figure S16E is that the dual or multisite mutations need to be within the region that the two primers can reach. By comparison, the sequential strategy depicted in Figure S11B does not have this limitation. Thus, for multi-site mutagenesis at distant sites, this strategy saves time and reduces labor and reagent costs resulting from plasmid isolation and sequencing.

The results presented herein demonstrate that due to formation of G-quadruplexes, G-stretches pose serious challenges to site-directed mutagenesis in vitro and that with new modifications, P3b site-directed mutagenesis works efficiently with the KAT2B, sgRNA/Cas9 and CDK13 expression plasmids that harbor extremely GC-rich regions (Figure 2, Figure 5, Figure 6, Figures S13 and S14). Notably, KAT2B and sgRNA/Cas9 plasmids share the CAG promoter, whereas both KAT2B and CDK13 expression plasmids possess GC-rich sequences encoding intrinsically disordered domains. Moreover, the CDK13 expression plasmid contains GC-rich regions within the IRES and the coding sequence for the puromycin-resistant marker (Figure 6D). This new method should be beneficial for engineering other plasmids with GC-rich regions. Potential plasmids are those with intrinsically disordered domains rich with alanine, glycine, proline and arginine residues, all of which are encoded by the G/C-G/C-G/C codons. Other potential plasmids are those with the CAG promoter as the KAT2B plasmid carries [24,25].

Clinically, GC-rich repeats are associated with various diseases. The hexanucleotide GGGGCC repeats are genetic causes of amyotrophic lateral sclerosis and frontotemporal dementia [56]. GCG repeat expansion underlies oculopharyngeal muscular dystrophy [57]. GGC repeat expansion is the cause of Fragile X syndrome [58]. It will be interesting to investigate whether such nucleotide sequences affect the site-directed mutagenesis efficiency with related plasmid vectors.

The P3b method complements P3 and P3a site-directed mutagenesis [5,6,7,8,9,10]. We propose an operational model on how to select P3a or P3b mutagenesis based on characteristics of a given plasmid template (Figure 7E,F). For P3a mutagenesis, either Q5 or SuperFi II polymerase can be used [10], but for P3b mutagenesis, SuperFi II polymerase is required (Figure 7F) as it is much more efficient than Q5 polymerase in such a scenario (Figure 2B and Figure 6B). Together, these two new methods provide a robust and versatile approach for single-site, multisite (Figure S11) or cassette (Figure 4A, Figure 5E and Figure 6A) mutagenesis, thereby offering two reliable tools for protein, RNA and plasmid engineering in many biomedical research laboratories. Notably, deletion and insertion are two special cases of cassette replacement [10]. In addition to traditional use of site-directed mutagenesis, the P3a and P3b methods (Figure 7F) are expected to facilitate drug development resulting from rapid advances in AI-assisted antibody and other protein design [15,16,17,18,19].

## 5. Conclusions

In this study, we have developed a new method for efficient site-directed mutagenesis of plasmids with GC-rich sequences and have also suggested that such sequences impede DNA synthesis during PCR via formation of G-quadruplexes.

## Data Availability

Data and research materials are available from the corresponding author upon reasonable request.

## Supplementary Materials

The following supporting information can be downloaded at: https://www.mdpi.com/article/10.3390/cells14242016/s1, Figure S1: GC-contents of the BRD1, BRPF3 and KAT2B coding sequences; Figure S2: Extremely GC-rich region of the human KAT2B coding sequence; Figure S3: Unwanted deletion and insertion in candidates for the R15W mutant of BRPF3; Figure S4: Use of Q5 and Q5U DNA polymerases to generate BRPF3 mutants; Figure S5: Use of Q5 and Q5U DNA polymerases to engineer two BRPF3 mutants; Figure S6: P3a mutagenesis for efficient construction of a BRPF3 deletion mutant; Figure S7: Construction of SARS-CoV-2 spike mutants using uracil-containing templates; Figure S8: P3a mutagenesis of the KAT2B and dN88 expression plasmids leads to frequent deletions; Figure S9: Construction of a deletion mutant lacking the N-terminal 88 residues of KAT2B; Figure S10: Efficiency of P3b site-directed mutagenesis to generate different HAT mutants; Figure S11: P3a and P3b multisite mutagenesis to generate SARS-CoV-2 spike mutants; Figure S12: P3a mutagenesis of the dN123, dAG1.3 and dN88/dAG1.3 expression plasmids; Figure S13: Sanger sequencing analysis of the CAG promoter; Figure S14: Sanger sequencing analysis of the CAG promoter; Figure S15: P3b cassette mutagenesis to modify the CDK13 expression plasmid; Figure S16: Models on unwanted mutations and the efficiency from P3, P3a and P3b mutagenesis; Table S1. Primers to engineer and sequence the mutants of BRPF3, KAT2B and two SARSCoV-2 spike variants; Table S2. Primers to engineer and sequence the CDK13 and Cas9 mutants.

## References

[1] Hutchison C.A., Phillips S., Edgell M.H., Gillam S., Jahnke P., Smith M. (1978). Mutagenesis at a specific position in a DNA sequence. J. Biol. Chem..

[2] Weiner M.P., Costa G.L., Schoettlin W., Cline J., Mathur E., Bauer J.C. (1994). Site-directed mutagenesis of double-stranded DNA by the polymerase chain reaction. Gene.

[3] Fisher C.L., Pei G.K. (1997). Modification of a PCR-based site-directed mutagenesis method. Biotechniques.

[4] Li S., Wilkinson M.F. (1997). Site-directed mutagenesis: A two-step method using PCR and DpnI. Biotechniques.

[5] Zheng L., Baumann U., Reymond J.L. (2004). An efficient one-step site-directed and site-saturation mutagenesis protocol. Nucleic Acids Res..

[6] Liu H., Naismith J.H. (2008). An efficient one-step site-directed deletion, insertion, single and multiple-site plasmid mutagenesis protocol. BMC Biotechnol..

[7] Qi D., Scholthof K.B. (2008). A one-step PCR-based method for rapid and efficient site-directed fragment deletion, insertion, and substitution mutagenesis. J. Virol. Methods.

[8] Mousavi N., Zhou E., Razavi A., Ebrahimi E., Varela-Castillo P., Yang X.J. (2025). P3 site-directed mutagenesis: An efficient method based on primer pairs with 3’-overhangs. J. Biol. Chem..

[9] Mousavi N., Zhou E., Razavi A., Ebrahimi E., Varela-Castillo P., Yang X.J. (2025). Efficient site-directed mutagenesis mediated by primer pairs with 3’-overhangs. Curr. Protoc. Protein Sci..

[10] Yang X.J. (2025). P3a site-specific and cassette mutagenesis for seamless protein, RNA and plasmid engineering. Genes Cancer.

[11] Varshney D., Spiegel J., Zyner K., Tannahill D., Balasubramanian S. (2020). The regulation and functions of DNA and RNA G-quadruplexes. Nat. Rev. Mol. Cell Biol..

[12] Batra S., Allwein B., Kumar C., Devbhandari S., Bruning J.G., Bahng S., Lee C.M., Marians K.J., Hite R.K., Remus D. (2025). G-quadruplex-stalled eukaryotic replisome structure reveals helical inchworm DNA translocation. Science.

[13] Insco M.L., Abraham B.J., Dubbury S.J., Kaltheuner I.H., Dust S., Wu C., Chen K.Y., Liu D., Bellaousov S., Cox A.M. (2023). Oncogenic CDK13 mutations impede nuclear RNA surveillance. Science.

[14] van den Akker W.M.R., Brummelman I., Martis L.M., Timmermans R.N., Pfundt R., Kleefstra T., Willemsen M.H., Gerkes E.H., Herkert J.C., van Essen A.J. (2018). De novo variants in *CDK13* associated with syndromic ID/DD: Molecular and clinical delineation of 15 individuals and a further review. Clin. Genet..

[15] Thean D.G.L., Chu H.Y., Fong J.H.C., Chan B.K.C., Zhou P., Kwok C.C.S., Chan Y.M., Mak S.Y.L., Choi G.C.G., Ho J.W.K. (2022). Machine learning-coupled combinatorial mutagenesis enables resource-efficient engineering of CRISPR-Cas9 genome editor activities. Nat. Commun..

[16] Starr T.N., Czudnochowski N., Liu Z., Zatta F., Park Y.J., Addetia A., Pinto D., Beltramello M., Hernandez P., Greaney A.J. (2021). SARS-CoV-2 RBD antibodies that maximize breadth and resistance to escape. Nature.

[17] Lisanza S.L., Gershon J.M., Tipps S.W.K., Sims J.N., Arnoldt L., Hendel S.J., Simma M.K., Liu G., Yase M., Wu H. (2025). Multistate and functional protein design using RoseTTAFold sequence space diffusion. Nat. Biotechnol..

[18] Kortemme T. (2024). De novo protein design—From new structures to programmable functions. Cell.

[19] Wasdin P.T., Johnson N.V., Janke A.K., Held S., Marinov T.M., Jordaan G., Gillespie R.A., Vandenabeele L., Pantouli F., Powers O.C. (2025). Generation of antigen-specific paired-chain antibodies using large language models. Cell.

[20] Ullah M., Pelletier N., Xiao L., Zhao S.P., Wang K., Degerny C., Tahmasebi S., Cayrou C., Doyon Y., Goh S.L. (2008). Molecular architecture of quartet MOZ/MORF histone acetyltransferase complexes. Mol. Cell. Biol..

[21] Yan K., You L., Degerny C., Ghorbani M., Liu X., Chen L., Li L., Miao D., Yang X.J. (2016). The chromatin regulator BRPF3 preferentially activates the HBO1 acetyltransferase but is dispensable for mouse development and survival. J. Biol. Chem..

[22] Yan K., Rousseau J., Machol K., Cross L.A., Agre K.E., Gibson C.F., Goverde A., Engleman K.L., Verdin H., Baere E. (2020). Deficient histone H3 propionylation by BRPF1-KAT6 complexes in neurodevelopmental disorders and cancer. Sci. Adv..

[23] Kitabayashi I., Aikawa Y., Nguyen L.A., Yokoyama A., Ohki M. (2001). Activation of AML1-mediated transcription by MOZ and inhibition by the MOZ-CBP fusion protein. EMBO J..

[24] Yang X.J., Ogryzko V.V., Nishikawa J., Howard B.H., Nakatani Y. (1996). A p300/CBP-associated factor that competes with the adenoviral oncoprotein E1A. Nature.

[25] Miyazaki J., Takaki S., Araki K., Tashiro F., Tominaga A., Takatsu K., Yamamura K. (1989). Expression vector system based on the chicken beta-actin promoter directs efficient production of interleukin-5. Gene.

[26] Martsen E.O., Zeng M., Lapeyre J.N. (1997). Fast plasmid DNA sequencing using a thermal cycler and high temperature alkali denaturation. Biotechniques.

[27] Tao Y., Zhong C., Zhu J., Xu S., Ding J. (2017). Structural and mechanistic insights into regulation of HBO1 histone acetyltransferase activity by BRPF2. Nucleic Acids Res..

[28] Kunkel T.A. (1985). Rapid and efficient site-specific mutagenesis without phenotypic selection. Proc. Natl. Acad. Sci. USA.

[29] McClary J.A., Witney F., Geisselsoder J. (1989). Efficient site-directed in vitro mutagenesis using phagemid vectors. Biotechniques.

[30] Yang X.J., Kaufman S. (1994). High-level expression and deletion mutagenesis of human tryptophan hydroxylase. Proc. Natl. Acad. Sci. USA.

[31] Trower M.K. (1994). Site-directed mutagenesis using a uracil-containing phagemid template. Methods Mol. Biol..

[32] Li F., Liu S.L., Mullins J.I. (1999). Site-directed mutagenesis using uracil-containing double-stranded DNA templates and DpnI digestion. Biotechniques.

[33] Yang S., Yu Y., Xu Y., Jian F., Song W., Yisimayi A., Wang P., Wang J., Liu J., Yu L. (2024). Fast evolution of SARS-CoV-2 BA.2.86 to JN.1 under heavy immune pressure. Lancet Infect. Dis..

[34] Yang H., Guo H., Wang A., Cao L., Fan Q., Jiang J., Wang M., Lin L., Ge X., Wang H. (2024). Structural basis for the evolution and antibody evasion of SARS-CoV-2 BA.2.86 and JN.1 subvariants. Nat. Commun..

[35] Hu G., Katuwawala A., Wang K., Wu Z., Ghadermarzi S., Gao J., Kurgan L. (2021). flDPnn: Accurate intrinsic disorder prediction with putative propensities of disorder functions. Nat. Commun..

[36] Fischer V., Plassard D., Ye T., Reina-San-Martin B., Stierle M., Tora L., Devys D. (2021). The related coactivator complexes SAGA and ATAC control embryonic stem cell self-renewal through acetyltransferase-independent mechanisms. Cell Rep..

[37] Albaugh B.N., Denu J.M. (2021). Catalysis by protein acetyltransferase Gcn5. Biochim. Biophys. Acta Gene Regul Mech..

[38] Paeschke K., Simonsson T., Postberg J., Rhodes D., Lipps H.J. (2005). Telomere end-binding proteins control the formation of G-quadruplex DNA structures in vivo. Nat. Struct. Mol. Biol..

[39] Huppert J.L., Balasubramanian S. (2007). G-quadruplexes in promoters throughout the human genome. Nucleic Acids Res..

[40] Cong L., Ran F.A., Cox D., Lin S., Barretto R., Habib N., Hsu P.D., Wu X., Jiang W., Marraffini L.A. (2013). Multiplex genome engineering using CRISPR/Cas systems. Science.

[41] Ran F.A., Hsu P.D., Wright J., Agarwala V., Scott D.A., Zhang F. (2013). Genome engineering using the CRISPR-Cas9 system. Nat. Protoc..

[42] Agudelo D., Duringer A., Bozoyan L., Huard C.C., Carter S., Loehr J., Synodinou D., Drouin M., Salsman J., Dellaire G. (2017). Marker-free coselection for CRISPR-driven genome editing in human cells. Nat. Methods.

[43] Pedrazzoli E., Bianchi A., Umbach A., Amistadi S., Brusson M., Frati G., Ciciani M., Badowska K.A., Arosio D., Miccio A. (2023). An optimized SpCas9 high-fidelity variant for direct protein delivery. Mol. Ther..

[44] Vos P.D., Gandadireja A.P., Rossetti G., Siira S.J., Mantegna J.L., Filipovska A., Rackham O. (2024). Mutational rescue of the activity of high-fidelity Cas9 enzymes. Cell Rep. Methods.

[45] Vos P.D., Rossetti G., Mantegna J.L., Siira S.J., Gandadireja A.P., Bruce M., Raven S.A., Khersonsky O., Fleishman S.J., Filipovska A. (2022). Computationally designed hyperactive Cas9 enzymes. Nat. Commun..

[46] Kim Y.H., Kim N., Okafor I., Choi S., Min S., Lee J., Bae S.M., Choi K., Choi J., Harihar V. (2023). Sniper2L is a high-fidelity Cas9 variant with high activity. Nat. Chem. Biol..

[47] Chey Y.C.J., Gierus L., Lushington C., Arudkumar J.C., Geiger A.B., Staker L.G., Robertson L.J., Pfitzner C., Kennedy J.G., Lee R.H.B. (2025). Optimal SpCas9- and SaCas9-mediated gene editing by enhancing gRNA transcript levels through scaffold poly-T tract reduction. BMC Genom..

[48] Sifrim A., Hitz M.P., Wilsdon A., Breckpot J., Turki S.H., Thienpont B., McRae J., Fitzgerald T.W., Singh T., Swaminathan G.J. (2016). Distinct genetic architectures for syndromic and nonsyndromic congenital heart defects identified by exome sequencing. Nat. Genet..

[49] Marsolier-Kergoat M.C., Yeramian E. (2009). GC content and recombination: Reassessing the causal effects for the Saccharomyces cerevisiae genome. Genetics.

[50] Kiktev D.A., Dominska M., Zhang T., Dahl J., Stepchenkova E.I., Mieczkowski P., Burgers P.M., Lujan S., Burkholder A., Kunkel T.A. (2021). The fidelity of DNA replication, particularly on GC-rich templates, is reduced by defects of the Fe-S cluster in DNA polymerase delta. Nucleic Acids Res..

[51] Robinson J., Raguseo F., Nuccio S.P., Liano D., Di Antonio M. (2021). DNA G-quadruplex structures: More than simple roadblocks to transcription?. Nucleic Acids Res..

[52] Qiu Y., Kang Y.M., Korfmann C., Pouyet F., Eckford A., Palazzo A.F. (2024). The GC-content at the 5’ ends of human protein-coding genes is undergoing mutational decay. Genome Biol..

[53] Hanecak R., Pattengale P.K., Fan H. (1991). Deletion of a GC-rich region flanking the enhancer element within the long terminal repeat sequences alters the disease specificity of Moloney murine leukemia virus. J. Virol..

[54] Schroder C., Horsthemke B., Depienne C. (2021). GC-rich repeat expansions: Associated disorders and mechanisms. Med. Genet..

[55] Hansel-Hertsch R., Beraldi D., Lensing S.V., Marsico G., Zyner K., Parry A., Di Antonio M., Pike J., Kimura H., Narita M. (2016). G-quadruplex structures mark human regulatory chromatin. Nat. Genet..

[56] Breevoort S., Gibson S., Figueroa K., Bromberg M., Pulst S. (2022). Expanding Clinical Spectrum of C9ORF72-Related Disorders and Promising Therapeutic Strategies: A Review. Neurol. Genet..

[57] Trollet C., Anvar S.Y., Venema A., Hargreaves I.P., Foster K., Vignaud A., Ferry A., Negroni E., Hourde C., Baraibar M.A. (2010). Molecular and phenotypic characterization of a mouse model of oculopharyngeal muscular dystrophy reveals severe muscular atrophy restricted to fast glycolytic fibres. Hum. Mol. Genet..

[58] Verkerk A.J., Pieretti M., Sutcliffe J.S., Fu Y.H., Kuhl D.P., Pizzuti A., Reiner O., Richards S., Victoria M.F., Zhang F.P. (1991). Identification of a gene (FMR-1) containing a CGG repeat coincident with a breakpoint cluster region exhibiting length variation in fragile X syndrome. Cell.

