# Supplementary material for: Highly Efficient Site-Specific and Cassette Mutagenesis of Plasmids Harboring GC-Rich Sequences
Source: Cells. 2025 Dec 18;14(24):2016. doi: 10.3390/cells14242016 (PMC12731741; doi:10.3390/cells14242016)
Supplement: Supplementary file 1 [file cells-14-02016-s001.zip › cells-3964925-supplementary.pdf]

Highly efficient site-specific and cassette mutagenesis of plasmids harboring GC-rich sequences

Paulina Varela-Castillo[1,2], Ethan Zhou[1,3,†], Arezousadat Razavi[1,2,†], Elham Ebrahimi[1,3] and Xiang-Jiao Yang[1,2,3,4,*]

[1]Rosalind and Morris Goodman Cancer Institute, McGill University, Montreal, Quebec H3A 1A3, Canada

[2]Department of Medicine, McGill University, Montreal, Quebec H3A 1A3, Canada

[3]Department of Biochemistry, McGill University, Montreal, Quebec H3A 1A3, Canada

[4]McGill University Health Center, Montreal, Quebec H3A 1A3, Canada

[†]The first two authors made equal contributions to this work.

*Correspondence: xiang-jiao.yang@mcgill.ca

Supplementary Figures S1-S16 and Tables S1-S2

Supplementary Figure legends

Figure S1 GC-contents of the BRD1, BRPF3 and KAT2B coding sequences

A. Distribution of the GC-content along the coding sequence of human *BRD1* (a.k.a. BRPF2). The coding sequence was copied and pasted into the calculation box provided by the VectorBuilder website. The GC-content was calculated with a window size of 20.

B. Same as panel A, but the BRPF3 coding sequence was analyzed. The primer site for the R15W mutant is indicated. Notably, P3 mutagenesis with PfuUltra or Pfu-fly failed to engineer this mutant [7]. While it was challenging to engineer BRPF3 mutants with P3 mutagenesis based on PfuUltra or Pfu-fly, the method was reliable for generating BRD1 mutants [7], suggesting that short GC-rich sequences may or may not act as barriers to P3 mutagenesis.

C. Same as panel A, but the KAT2B coding sequence was analyzed. The extremely GC-rich region in the 5′-region is marked. A portion of this GC-rich sequence is shown in Fig. S2A.

Figure S2 Extremely GC-rich region of the human KAT2B coding sequence

A. Analysis of three plasmids sequenced for engineering the KAT2B mutant C100A. Only a portion of the extremely GC-rich sequence (encoding amino acid residues 3-34) is shown (top). Notably, despite the presence of this GC-rich region, the quality of Sanger sequencing was excellent (bottom).

B. The sequences of human (h) KAT2A, its paralog, their mouse (m) counterparts and the ortholog in *Drosophila* (Gcn5) were aligned with Clustal Omega within the SnapGene software package (version 8.0). The intrinsically disordered domain is indicated by a solid line. This domain is evolutionarily more divergent than other regions of the proteins.

Figure S3 Unwanted deletion and insertion in candidates for the R15W mutant of BRPF3

A. Analysis of seven plasmids sequenced for engineering the R15W mutant of BRPF3. The first six clones were from P3 mutagenesis reactions with Pfu-fly polymerase and the 7th was from a P3a mutagenesis reaction using Q5U polymerase and the uracil-containing BRPF3 plasmid. A potential self-priming site and its identical sequences in the insertions are boxed in blue.

B. Same as (A), except a more downstream region is shown.

C. Same as (B), but the 3′-ends of the deleted regions are shown. Notably, despite Q5U polymerase being used for C7, the deletion is identical among clones C1, C3 and C4. Clone C2a is absent in panels A and B.

Figure S4 Use of Q5 and Q5U DNA polymerases to generate BRPF3 mutants

A. Domain organization of BRPF3. It is composed of an N-terminal domain (N) for interacting with KAT7 [27], two Enhancer of Polycomb (EPC)-like motifs (I and II), a PhD-zinc knuckle-PHD (PZP) module, a bromodomain and a Pro-Trp-Trp-Pro (PWWP) domain [27]. Four missense variants are depicted, with three located within the N domain (R15W, R51H and I52A) and one in the PWWP domain (E1075K). Due to an unknown reason, the E1075K substitution was unexpectedly found to be present on the expression vector that we used, so K1075E was to repair this substitution.

B. Efficiency comparison of P3a mutagenesis mediated by Q5 and Q5U DNA polymerases. For the latter, the uracil-containing BRPF3 expression plasmid isolated from CJ236 was used. Regular plasmid isolated from DH5α was employed for Q5 polymerase. In both cases, we analyzed three plasmids per mutation. P3a mutagenesis with Q5 polymerase yielded 10 correct mutants out of 12 plasmids sequenced. Among the remaining two, one was wild-type and the

other contained an unexpected C deletion (shown in the third R15W candidate in panel C), potentially due to primer impurities. Among the 13 plasmids sequenced for the Q5U condition, three was wild-type (with the unrepaired E1075K mutant considered as the "wild-type") and three contained unexpected deletions or insertions. Thus, Q5U with the uracil-containing template was much less reliable than Q5 DNA polymerase with regular plasmids.

C. Analysis of six plasmids sequenced for engineering the R15W variant. For the Q5 or Q5U condition, three plasmids were sequenced per mutation. One candidate from the Q5 condition exhibited an unexpected a cytosine deletion. This deletion is located at the primer site, so it could be due to primer impurities. The remaining two carried the correct mutation, leading to an efficiency of 66.7%. In contrast, one candidate from the Q5U condition was wild-type and another carried 3,344-bp deletion, resulting in an efficiency of 33.3%. Notably, this large deletion is recurrent under the conditions using Pfu-fly (Fig. S4) [7].

D. Analysis of five plasmids sequenced for engineering the K1057E substitution. One candidate from the Q5 condition failed to be sequenced, and one candidate from the Q5U condition was the unrepaired clone, thus yielding an efficiency of 66.7% for both conditions.

Figure S5 Use of Q5 and Q5U DNA polymerases to engineer two BRPF3 mutants

Analysis of 13 plasmids sequenced for engineering the R51H and I52A mutants of BRPF3. For the Q5 and Q5U conditions, 6 and 7 plasmids were sequenced, respectively. All 6 plasmids from the Q5 condition were mutants, so the efficiency was 100%. The results from the Q5U condition were mixed: While the 4 plasmids sequenced for the R51H mutant were all correct, none of the three sequenced for the I52A mutant were correct. Among them, one was wild-type and the other two possessed either a deletion or an insertion at the primer site.

Figure S6 P3a mutagenesis for efficient construction of a BRPF3 deletion mutant

A. Coding sequence of the N-terminal part of BRPF3. The 192-bp DNA fragment to be deleted is highlighted in blue.

B. Design of two primers to engineer the dN65 mutant of BRPF3. The coding sequence for the N-terminal 65 residues of wild-type BRPF3 is deleted via the primers dN65-F and dN65-R.

C. Analysis of three plasmids sequenced for engineering the dN65 mutant. All three were correct, so the efficiency was 100%.

Figure S7 Construction of SARS-CoV-2 spike mutants using uracil-containing templates

A. Efficiency for generating different spike mutants. The mutants were produced with an expression plasmid for the D614G spike protein of SARS-CoV-2. The plasmid was isolated from CJ236 and used for P3a mutagenesis mediated by Q5U DNA polymerase. For each mutation, three plasmids were sequenced. Among the 12 plasmids sequenced, one was wild-type and the remaining 11 were the correct mutants, leading to an efficiency of 91.7%.

B. Efficiency for generating different spike mutants. The mutants were generated with an expression plasmid for the Omicron spike protein of SARS-CoV-2. The plasmid was isolated from CJ236 for P3a mutagenesis as in (A). Per mutation reaction, three plasmids were sequenced. Among the 12 plasmids sequenced, only 5 were the correct mutants, yielding an efficiency of 41.7%.

C. Analysis of 3 plasmids sequenced for engineering the SARS-CoV-2 spike mutation Q493E. One was wild-type and two were the mutant, resulting in the efficiency of 66.7%.

D. Analysis of 3 plasmids sequenced for engineering the single mutant F456L and the double mutant L455S_F456L of Omicron spike protein. A single pair of primers was used in the same mutagenesis reaction for engineering both mutants. Among the three plasmids sequenced, one was the double mutant L455S_F456L, another was a F456L clone with a small percentage of the double mutant L455S_F456L, and the third clone failed to be sequenced. Thus, the mutagenesis efficiency was 66.7%.

Figure S8 P3a mutagenesis of the KAT2B and dN88 expression plasmids leads to frequent deletions

A. Plasmids from 12 colonies from engineering the C100A, Y189A and G447W mutants via P3a mutagenesis were double-digested with EcoRI and PstI. The positive control (the plasmid for wild-type KAT2B) was loaded on lane 2. Only two out of 16 plasmids (lanes 4 & 6) exhibited the expected digestion pattern, with the rest possessing deletions.

B. Same as Panel A, but plasmids from 4 colonies for engineering the E570Q mutant were analyzed. Only one of the plasmids showed the expected digestion pattern (lane 5).

C. Same as panels A-B, but an independent experiment for engineering the C100A and E570Q mutants via P3a mutagenesis. The positive control (the plasmid for wild-type KAT2B) was loaded on lane 1. Only one out of 8 plasmids (lane 7) exhibited the correct digestion pattern, with the rest containing deletions.

D. Same as panels A-B, but the dN88 expression plasmid (Fig. S11) was used as the template for P3a mutagenesis for engineering C100A. Out of 4 plasmids analyzed, only the candidate corresponding to lane 3 showed the expected digestion pattern. Sequencing analysis revealed that

it possesses the C100A mutation, so the mutagenesis efficiency was 25% (1/4). Noe that the five lanes were from the same gel as those in (C), with lane 5 identical to lane 1 in (C).

E. Same as panels A-B, but the dN88 expression plasmid (Fig. S11) was used as the template for P3a mutagenesis for engineering C108A, H141A and Y189A. Out of 12 plasmids analyzed, the candidates corresponding to lanes 3 and 5-8 showed the expected digestion patterns. Sequencing analysis revealed that the one shown in lane 3 possesses an insertion and those in lanes 5-8 were all wild-type.

F. Same as Panel D, but P3b mutagenesis without the extra denaturation step prior to PCR was employed. Out of 12 plasmids analyzed, only the candidates corresponding to lanes 4 and 10 did not show the expected digestion pattern.

Figure S9 Construction of a deletion mutant lacking the N-terminal 88 residues of KAT2B

A. Coding sequence of the N-terminal part of KAT2B. The 0.27 kb DNA fragment to be deleted via P3b mutagenesis is highlighted in blue.

B. Design of two primers to engineer the dN88 mutant of KAT2B. The coding sequence for the N-terminal 88 residues of wild-type KAT2B is deleted via P3b mutagenesis with primers dN88-F and dN88-R.

C. Sequence chromatograms of five plasmids sequenced from engineering the dN88 deletion mutant. Initially 12 plasmids were subject to double digestion with EcoRI and PstI to select the ones with the expected deletion, which identified 6 plasmids. All six of them were sequenced (with 5 shown here) and found to carry the correct deletion, resulting in the efficiency of 50%.

Figure S10 Efficiency of P3b site-directed mutagenesis to generate different HAT mutants

A. Efficiency of P3b mutagenesis to engineer four KAT6A mutants. Prior to PCR, the KAT6A expression plasmid was heat-denatured at 105°C before PCR with SuperFi II polymerase. We analyzed three plasmids per mutation. Among the 12 plasmids sequenced, none were wild-type, one K604R candidate possessed an extra mutation (G497V) and two S670P candidates shared a large deletion (see panel C), resulting in an efficiency of 75% (9/12).

B. Efficiency of P3b mutagenesis to engineer four p300 and CBP mutants using SuperFi II polymerase. Prior to PCR, the expression plasmids were heat-denatured at 105°C before PCR with SuperFi II polymerase. We analyzed three plasmids per mutation reaction. Among the 12 plasmids sequenced, three were wild-type and two failed to be sequenced, leading to an efficiency of 7/12 (58.3%).

C. Analysis of three plasmids sequenced from engineering the S670P mutant of KAT6A. One was correct, indicating the efficiency of 33.3%. The remaining two contained a 1,725-bp deletion at a primer site.

D. Sequences of of three plasmids from generating the D1399Y and D1399H mutants of p300. A single pair of primers was used for generating both mutants. Among the three plasmids sequenced, two encoded D1399Y and one was a mixed clone for D1399Y and D1399H, yielding an efficiency of 100%.

Figure S11 P3a and P3b multisite mutagenesis to generate SARS-CoV-2 spike mutants

A. Schematic illustration of a typical P3a or P3b mutagenesis cycle. It takes 4-5 days per cycle.

B. A strategy to engineer mutations at multiple sites. On day 1, mutagenesis is carried out and the reaction mixture is transformed into DH5α. The mixed bacteria from transformation are directly inoculated LB media to grow 5-6 ml bacterial culture. On day 2, plasmids are isolated

from the culture and used directly for the second round of mutagenesis. This cycle is repeated every day until the last cycle, which will be carried out as depicted in (A). For simplicity, only P3a mutagenesis is shown in (A-B), but the strategy also applies to P3b mutagenesis.

C. Domain organization of the SARS-CoV-2 spike protein. Six substitutions to be engineered are depicted. Primer V320-F was used to sequence the first four mutations and primer Q965-F was for sequencing the last two. NTD, N-terminal domain; RBD, receptor-binding domain; CTD, C-terminal domain; S1/S2, S1/S2 cleavage site; FP, fusion peptide; FPPR, fusion peptide proximal region; HR1, heptad repeat 1; HR2, heptad repeat 2; TM, transmembrane anchor; CT, cytoplasmic tail.

D. Efficiency of P3b multisite mutagenesis to engineer five SARS-CoV-2 spike mutants. A single pair of primers was used to engineer the L455S_F456L and F456L mutants. Sequential mutagenesis cycles were carried out with different pairs of primers. For each of the first three (Omicron variant) or four (D614G variant) mutagenesis cycles, bacteria from transformation were not plated out; instead, they were directly inoculated into LB media containing ampicillin as depicted in (B). Plasmids were isolated and used for the second cycle of mutagenesis. This cycle was repeated until the third (for the Omicron variant) or fourth (for the D614G variant) cycles, where the bacteria from each reaction were plated out on an LB-agar plate containing ampicillin to produce single colonies. Plasmids from 4-10 colonies were prepared for Sanger sequencing. The presence of the six mutations on the plasmids is tabulated here, with sequencing chromatograms shown in panels E-H.

E-H. Sequencing chromatograms illustrating mutagenesis outcomes at site 1 (R346T, E), site 2 (L455S/F456L and F456L, F), site 3 (Q493E, G), site 4 (L981F, not shown) and site 5 (V1104L, H). For P3b mutagenesis with the plasmid expressing the spike protein of the SARS-CoV-2

D614G variant, 4 colonies were sequenced and two failed to be sequenced when the primer V320-F was used, so they were counted as two unsucessful candidates.

Figure S12 P3a mutagenesis of the dN123, dAG1.3 and dN88/dAG1.3 expression plasmids

A. Plasmids from 12 colonies resulting from engineering the C100A, C108A, H141A and Y189A mutants via P3a mutagenesis of the dN123 expression plasmid were digested with EcoRI and PstI. The positive control (the plasmid for wild-type KAT2B) was loaded on lane 2. None of plasmids exhibited the expected digestion pattern.

B. Same as panel A, but 12 colonies from engineering the C100A, C108A, H141A and Y189A mutants from the vector dAG1.3 were analyzed.

C-D. Same as panel B, but the dN88/dAG1.3 expression plasmid was used as the template for P3a mutagenesis. All showed the expected digestion patterns.

Figure S13 Sanger sequencing analysis of the CAG promoter

A. Cartoon showing some key features of a CAG-Cas9 expression plasmid, along with a sequencing primer (CMV-F1) located at the CMV promoter.

B. The pCX-KAT2B expression plasmid was subject to Sanger sequencing with the primer CMV-F1 located at the CMV promoter, upstream from the β-action promoter. The sequencing reaction terminated prematurely after a stretch of 27 Gs, indicative of DNA synthesis difficulty *in vitro*. For the sequencing reaction, the denaturation temperature was 96°C and a hot-start DNA polymerase was from a typical sequencing kit compatible with the Applied Biosystems Sequencer 3730XL. Notably, with the same sequencing method, the GC-rich region encoding the

N-terminal part of KAT2B was sequenced more easily (Fig. S2A). Note several mis-incorporated nucleotides, highlighted in red in the expected sequence shown at the bottom of the panel.

C. Sequence for a part of the CAG promoter analyzed as in (B). The regions forming G-quadruplxes are boxed or underlined in red, with the latter corresponding to that led to the premature termination during sequencing with primer CMV-F1 (see panel A). The boxed region corresponds to the one that led to the premature termination during sequencing with primer CAG-R (see Fig. S14).

Figure S14 Sanger sequencing analysis of the CAG promoter

A. Cartoon showing some key features of a CAG-Cas9 expression plasmid, including a sequencing primer (CAG-R) located at the β-action promoter and the hybrid intron.

B. The plasmid was sequenced with the CAG-R primer. The sequencing reaction terminated prematurely after a stretch of Gs, indicating that DNA synthesis *in vitro* was stalled. For the sequencing reaction, the denaturation temperature was 96°C and a hot-start DNA polymerase was from a typical sequencing kit compatible with the Applied Biosystems Sequencer 3730XL.

Figure S15 P3b cassette mutagenesis to modify the CDK13 expression plasmid

A. Sequence showing a 39-bp GC-rich region upstream from the IRES. Also see Fig. 6D.

B. Sequencing results of 5 plasmids from deletion mutagenesis of the 39-bp fragment. All five (C1-C5) contained the expected deletion.

C. Sequencing results of 3 plasmids from deletion mutagenesis of a 0.6 kb GC-rich fragment encoding the puromycin-resistant marker. All three (C1-C3) possessed the expected deletion. See Fig. 6D for the GC-content of this fragment.

Figure S16 Models on unwanted mutations and the efficiency from P3, P3a and P3b mutagenesis

A. Schematic illustrating that faithful synthesis termination at the 5′-end of an annealed primer is critical for maintaining the correct sequences at the primer site. If the new strand fails to terminate when reaching the 5′-end of an annealed primer, insertion may occur, as observed with the R15W mutant of BRPF3 (Fig. S3A-C).

B. Cartoon showing how false priming leads to unwanted deletion. The blue loop represents a secondary structure that enables the primer to anneal and initiate new strand synthesis at a distant site different from the intended primer site. This may occur due to partial sequence similarity between the primary and secondary primer sites, as we have observed while engineering the R15W mutant of BRPF3 (Fig. S3). Intriguingly, superior polymerases such as SuperFi II and Q5 polymerases can prevent this, whereas Pfu-fly polymerase is prone to such errors.

C. Cartoon illustrating how different populations of plasmids in a mutagenesis reaction dictate the eventual efficiency. The population with unwanted mutations is not shown here. The mixed plasmid molecules are resistant to DpnI digestion [23].

D. Mathematical formula that governs the mutagenesis efficiency of P3a or P3b mutagenesis. As few wild-type clones were observed with P3a or P3b mutagenesis (this and a previous study [9]), the presence of wild-type plasmid molecules is not a key determinant of efficiency. Instead, candidates with unwanted mutations (highlighted in red) are a main determinant, underscoring the critical role of the polymerases used. This also explains why extra heat-denaturation did not improve the mutagenesis efficiency for the KAT6A, p300 and CBP expression plasmids (Fig. 4).

E. Schematic representation of a pair of primers containing two distinct mutations (as shown by red and green asterisks). After mutagenesis, the two primers lead to generation of two different mutants.

F. Schematic representation of a pair of primers containing two distinct mutations at the 3′-overhangs (as shown by red and green asterisks). After mutagenesis, the primers lead to generation of a mutant with two distinct mutations. Notably, the outcome of engineering two mutations at distant sites is somewhat different from what is depicted with the primer design shown in (E).

Figure S1

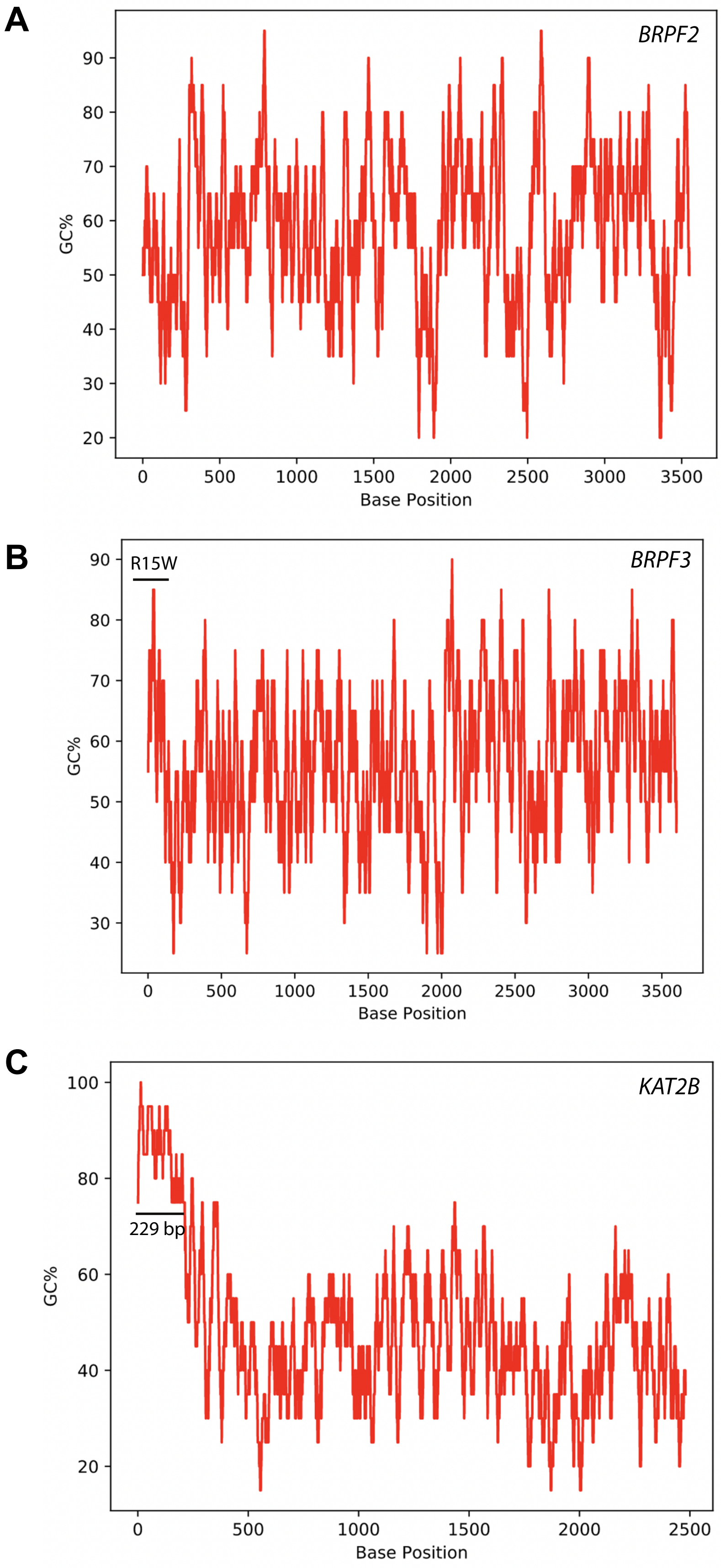
A
BRPF2
GC%
Base Position
B
R15W
BRPF3
GC%
Base Position
C
KAT2B
229 bp
GC%
Base Position

Figure S2A

A

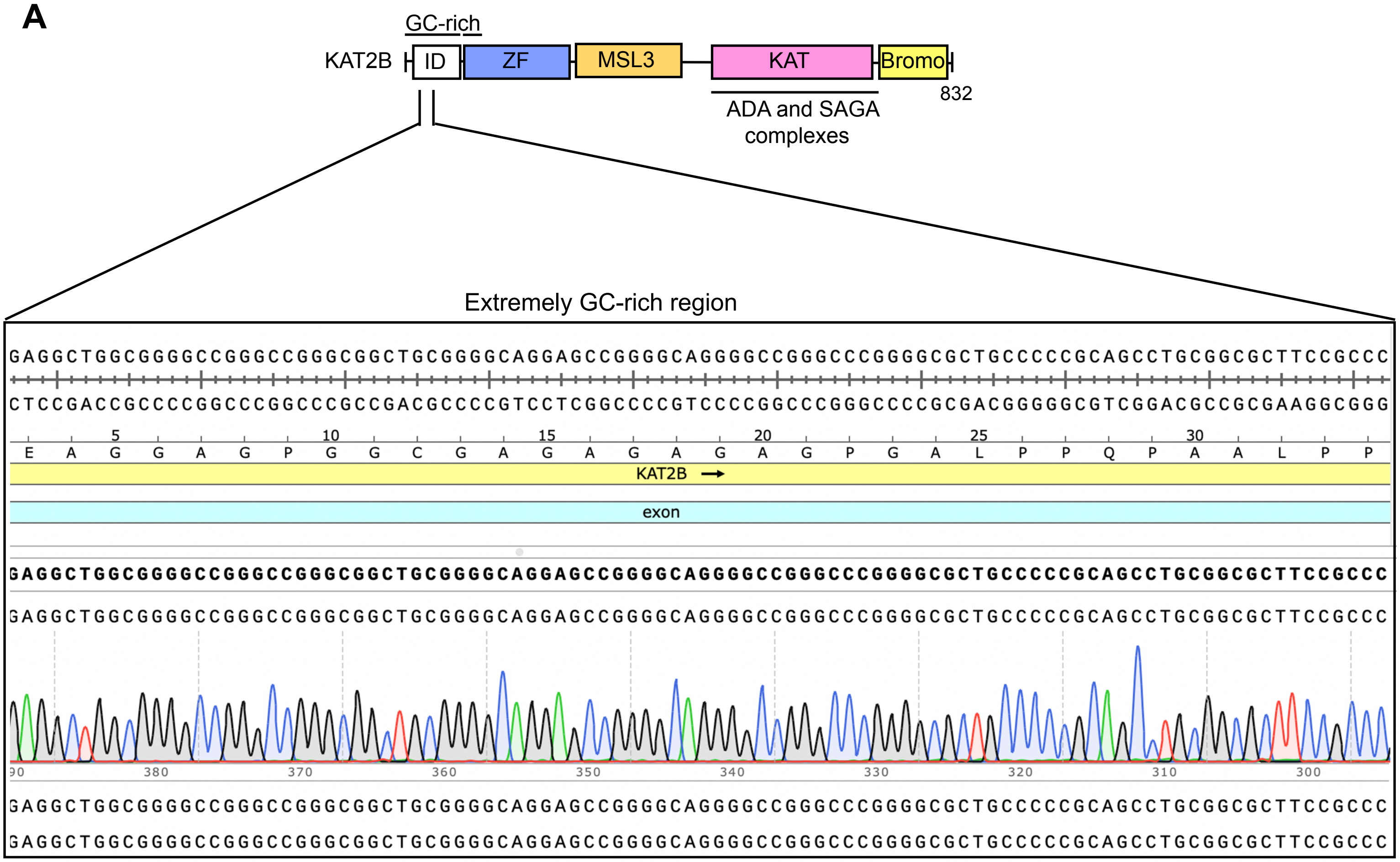
GC-rich
KAT2B
ID
ZF
MSL3
KAT
Bromo
832
ADA and SAGA complexes
Extremely GC-rich region
GAGGCTGGCGGGGCCGGGCCGGGCGGCTGCGGGGCAGGAGCCGGGGCAGGGGCCGGGCCCGGGGCGCTGCCCCCGCAGCCTGCGGCGCTTCCGCCC
CTCCGACCGCCCCGGCCCGGCCCGCCGACGCCCCGTCCTCGGCCCCGTCCCCGGCCCGGGCCCCGCGACGGGGGCGTCGGACGCCGCGAAGGCGGG
5 10 15 20 25 30
E A G G A G P G G C G A G A G A G A G P G A L P P Q P A A L P P
KAT2B
exon
GAGGCTGGCGGGGCCGGGCCGGGCGGCTGCGGGGCAGGAGCCGGGGCAGGGGCCGGGCCCGGGGCGCTGCCCCCGCAGCCTGCGGCGCTTCCGCCC
GAGGCTGGCGGGGCCGGGCCGGGCGGCTGCGGGGCAGGAGCCGGGGCAGGGGCCGGGCCCGGGGCGCTGCCCCCGCAGCCTGCGGCGCTTCCGCCC
390 380 370 360 350 340 330 320 310 300
GAGGCTGGCGGGGCCGGGCCGGGCGGCTGCGGGGCAGGAGCCGGGGCAGGGGCCGGGCCCGGGGCGCTGCCCCCGCAGCCTGCGGCGCTTCCGCCC
GAGGCTGGCGGGGCCGGGCCGGGCGGCTGCGGGGCAGGAGCCGGGGCAGGGGCCGGGCCCGGGGCGCTGCCCCCGCAGCCTGCGGCGCTTCCGCCC

B

Figure S2B

Intrinsically disordered (ID) domain

```
KAT2A (h)  MAEPSQAPTPAPAAQPRPLQSPAPAPTPTPAPSPASAPIPTPTPAPAPAPAAPAGSTGTGGPG----VGSGGAGSGGD-PARPGLSQQQRASQRKAQVR  95
KAT2B (h)  MSEAGGA-------GPGGCGAGAGAGAGPGALPPQPAALPPAPPQGSPC--AAAAGGSGACGPA----TAVAAA---GT-AEGPGGGGSARIAVKKAQLR  83
KAT2A (m)  MAEPSQAPNPVPAAQPRPLHSPAPAPTSTPAPSPASTPAPTPAPAPAPAAPAGSTGSGGAG----VG-----SGGD-PARPGLSQQQRASQRKAQVR  90
KAT2B (m)  MAEAGGA--------------------G-------SPALPPAPPHGSPRTLATAAGSSASCGPA----TAVAAA---GT-AEGPGGGGSARIAVKKAQLR  65
Gcn5 (f)   ---MSGGPSITIKSQPID-----GNNTGNAA----------AQQQQQAANGAATAGASGAAGSAQNPGHGGAASGAGSVPAEGTRQNSLQRIQQRKQKVF  82

KAT2A (h)  GLPRAKKLEKLGVFSACKANETCKCNGWKNPKP-PTAPRMDLQQPAANLSELCRSCEHPLADHVSHLENVSEDEINRLLGMVVDVENLFMSVHKEEDTDT  194
KAT2B (h)  SAPRAKKLEKLGVYSACKAEESCKCNGWKNPNPSPTPPRADLQQIIVSLTESCRSCSHALAAHVSHLENVSEEEMNRLLGIVLDVEYLFTCVHKEEDADT  183
KAT2A (m)  GLPRAKKLEKLGVFSACKANETCKCNGWKNPKP-PTAPRMDLQQPAANLSELCRSCEHPLADHVSHLENVSEDEINRLLGMVVDVENLFMSVHKEEDTDT  189
KAT2B (m)  SAPRAKKLEKLGVYSACKAEESCKCNGWKNPNPSPTPPRGDLQQIIVSLTESCRSCSHALAAHVSHLENVSEEEMDRLLGIVLDVEYLFTCVHKEEDADT  165
GCN5 (f)   NLPVPQKLAKLSMYSACQS-EGCRCTGWKTPQENRHRDVESSYCPEFNEECRNTSCRHSLRSHIAHLDNISSSSMNELLGAIIDMENLFMSMQRVEDEDT  181

KAT2A (h)  KQVYFYLFKLLRKCILQMTRPVVEGSLG-SPPFEKPNIEQGVLNFVQYKFSHLAPRERQTMFELSKMFLLCLNYWKLETPAQFRQRSQAEDVATYKVNYT  293
KAT2B (h)  KQVYFYLFKLLRKSILQRGKPVVEGSLEKKPPFEKPSIEQGVNNFVQYKFSHLPAKERQTIVELAKMFLNRINYWHLEAPSQRRLRSPNDDISGYKENYT  283
KAT2A (m)  KQVYFYLFKLLRKCILQMTRPVVEGSLG-SPPFEKPNIEQGVLNFVQYKFSHLAPRERQTMFELSKMFLLCLNYWKLETPAQFRQRSQSEDVATYKVNYT  288
KAT2B (m)  KQVYFYLFKLLRKSILQRGKPVVEGSLEKKPPFEKPSIEQGVNNFVQYKFSHLPSKERQTTIELAKMFLNRINYWHLEAPSQRRLRSPNDDISGYKENYT  265
GCN5 (f)   KKVYQYLFRLLRQCVLTRQQAVIRGPLG-DPPFETPCITKAVLSLVFYKYNHLSTPELQTMTEVAKTFLNFLNHYNFESPSTRRGDLTHEDASNYKINYT  280

KAT2A (h)  RWLCYCHVPQSCDSLPRYETTHVFGRSLLRSIFTVTRRQLLEKFRVEKDKLVPEKRTLILTHFPKFLSMLEEEIYGANSPIWESGFTMPPSEGTQLVP--  391
KAT2B (h)  RWLCYCNVPQFCDSLPRYETTQVFGRTLLRSVFTVMRRQLLEQARQEKDKLPLEKRTLILTHFPKFLSMLEEEVYSQNSPIWDQDFLSASSRTSQLGIQT  383
KAT2A (m)  RWLCYCHVPQSCDSLPRYETTHVFGRSLLRSIFTVTRRQLLEKFRVEKDKLVPEKRTLILTHFPKFLSMLEEEIYGANSPIWESGFTMPPSEGTQLVP--  386
KAT2B (m)  RWLCYCNVPQFCDSLPRYETTKVFGRTLLRSVFTIMRRQLLEQARQEKDKLPLEKRTLILTHFPKFLSMLEEEVYSQNSPIWDQDFLSASSRTSPLGIQT  365
GCN5 (f)   RWLVFCHVPAFCNSLRQCETSLVFGRTLLRTVFQCVSQQLKKKCISERDRFPEDKRS-IITLMPKFLETLRAELLKDDSPIWDTSYRPSNSFVIQQRK--  377

KAT2A (h)  --RPASVSA-AVVPS-TPIFSPSMGGGSNSSLSLDSAGAEPMP-GEKRTLPENLTLEDAKRLRVMGDIPMELVNEVMLTITDPAAMLGPETSLLSANAAR  486
KAT2B (h)  VINPPPVAG-TISYNSTSSSLEQPNAGSSSPACKASSGLE-ANPGEKRKMTDSHVLEEAKKPRVMGDIPMELINEVMSTITDPAAMLGPETNFLSAHSAR  481
KAT2A (m)  --RPATVSA-TVVP----SFSPSMGGGSNSSLSLDSAGTEPMPAGEKRKLPENLTLEDAKRLRVMGDIPMELVNEVMLTITDPAAMLGPETSLLSANAAR  479
KAT2B (m)  VISP-PVTG-TALFSSNSTSHEQINGGRTSPGCRGSSGLE-ANPGEKRKMNNSHAPEEAKRSRVMGDIPVELINEVMSTITDPAGMLGPETNFLSAHSAR  462
GCN5 (f)   --RNQEVASVPIGPSA-----ASIGGNKRT------SVGEPLHKRIKKEP------TDRPSSENLDDLPADVVMRAMKSVSESKTT-NKAEILFPVNVSR  457

KAT2A (h)  DETARLEERRGIIEFHVIGNSLTPKANRRVLLWLVGLQNVFSHQLPRMPKEYIARLVFDPKHKTLALIKDGRVIGGICFRMFPTQGFTEIVFCAVTSNEQ  586
KAT2B (h)  DEAARLEERRGVIEFHVVGNSLNQKPNKKILMWLVGLQNVFSHQLPRMPKEYITRLVFDPKHKTLALIKDGRVIGGICFRMFPSQGFTEIVFCAVTSNEQ  581
KAT2A (m)  DETARLEERRGIIEFHVIGNSLTPKANRRVLLWLVGLQNVFSHQLPRMPKEYIARLVFDPKHKTLALIKDGRVIGGICFRMFPTQGFTEIVFCAVTSNEQ  579
KAT2B (m)  DEAARLEERRGVIEFHVVGNSLNQKPNKKILMWLVGLQNVFSHQLPRMPKEYITRLVFDPKHKTLALIKDGRVIGGICFRMFPSQGFTEIVFCAVTSNEQ  562
GCN5 (f)   DENVKAEEQKRAIEFHVVGNSLTKPVDKQTVLWLLGLQLVFAYQLPEMPREYISQLVFDTKHKTLALIKENQPIGGICFRPFPSQGFTEIVFCAVTMSEQ  557
```

Figure S3

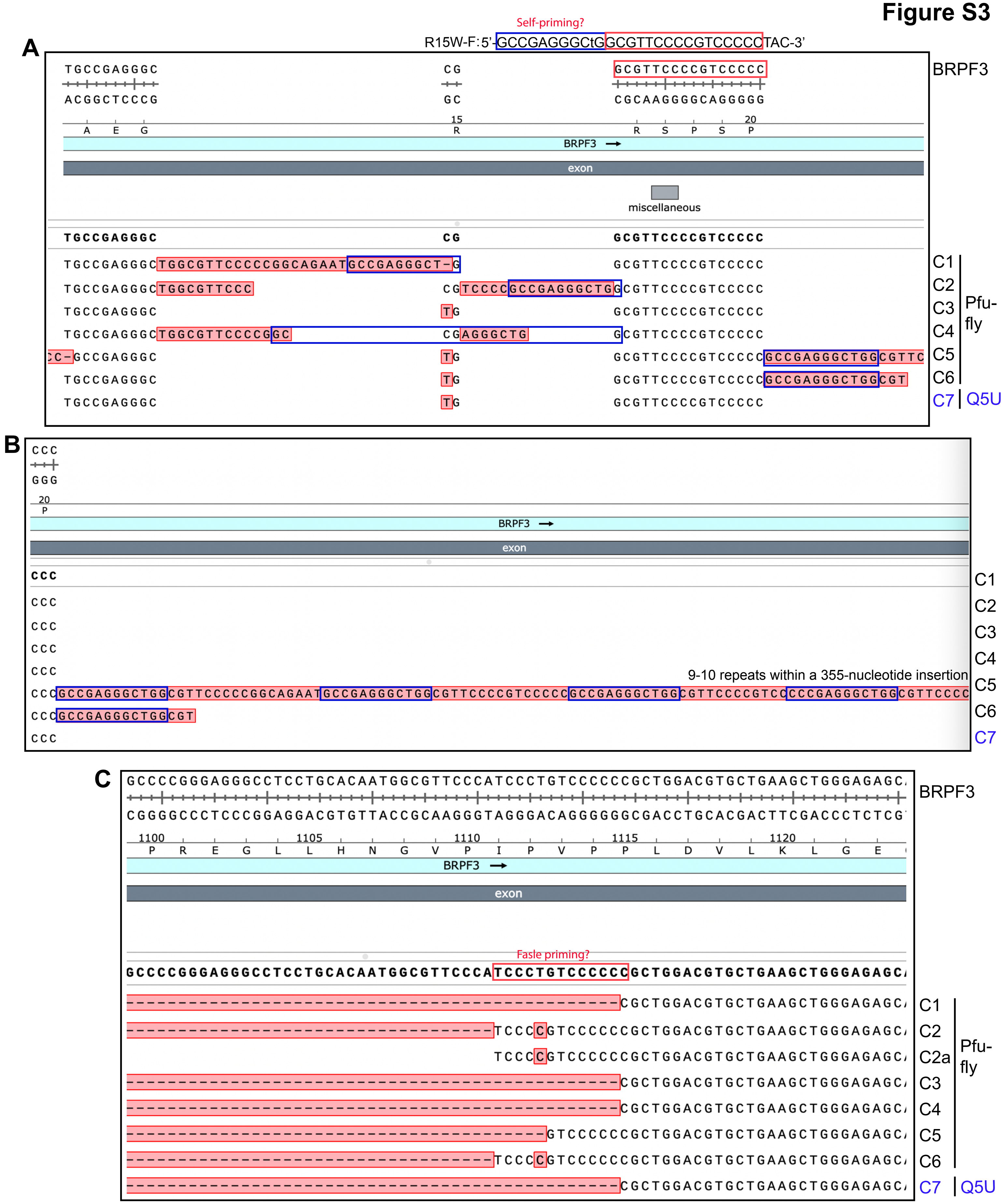

Self-priming?
R15W-F:5'-GCCGAGGGCtGGCGTTCCCCGTCCCCCTAC-3'
A
BRPF3
TGCCGAGGGC
CG
GCGTTCCCCGTCCCCC
ACGGCTCCCG
GC
CGCAAGGGGCAGGGGG
A E G
15
R
R S P S P
20
BRPF3
exon
miscellaneous
C1
C2
C3
C4
C5
C6
Pfu-fly
C7
Q5U
B
CCC
GGG
20
P
BRPF3
exon
9-10 repeats within a 355-nucleotide insertion
C
GCCCCGGGAGGGCCTCCTGCACAATGGCGTTCCCATCCCTGTCCCCCCGCTGGACGTGCTGAAGCTGGGAGAGC
CGGGGCCCTCCCGGAGGACGTGTTACCGCAAGGGTAGGGACAGGGGGGCGACCTGCACGACTTCGACCCTCTCG
1100
1105
1110
1115
1120
P R E G L L H N G V P I P V P P L D V L K L G E
BRPF3
exon
Fasle priming?
C2a

Figure S4

A

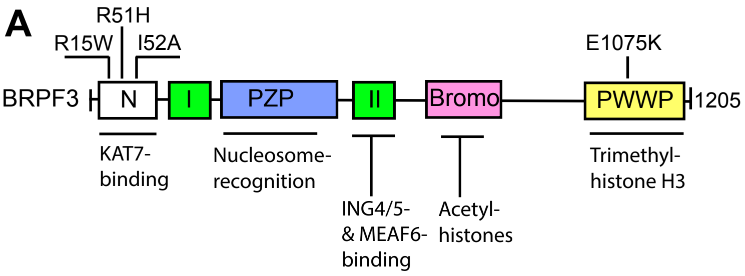

B

DNA Polymerase	Q5	Q5U
BRPF3 mutants attempted	4	4
Plasmids sequenced	12	12
WT	1	3
With expected mutation	10	7
With unexpected mutation*	1	3
Efficiency	10/12 (83.3%)	7/13 (53.8%)

C

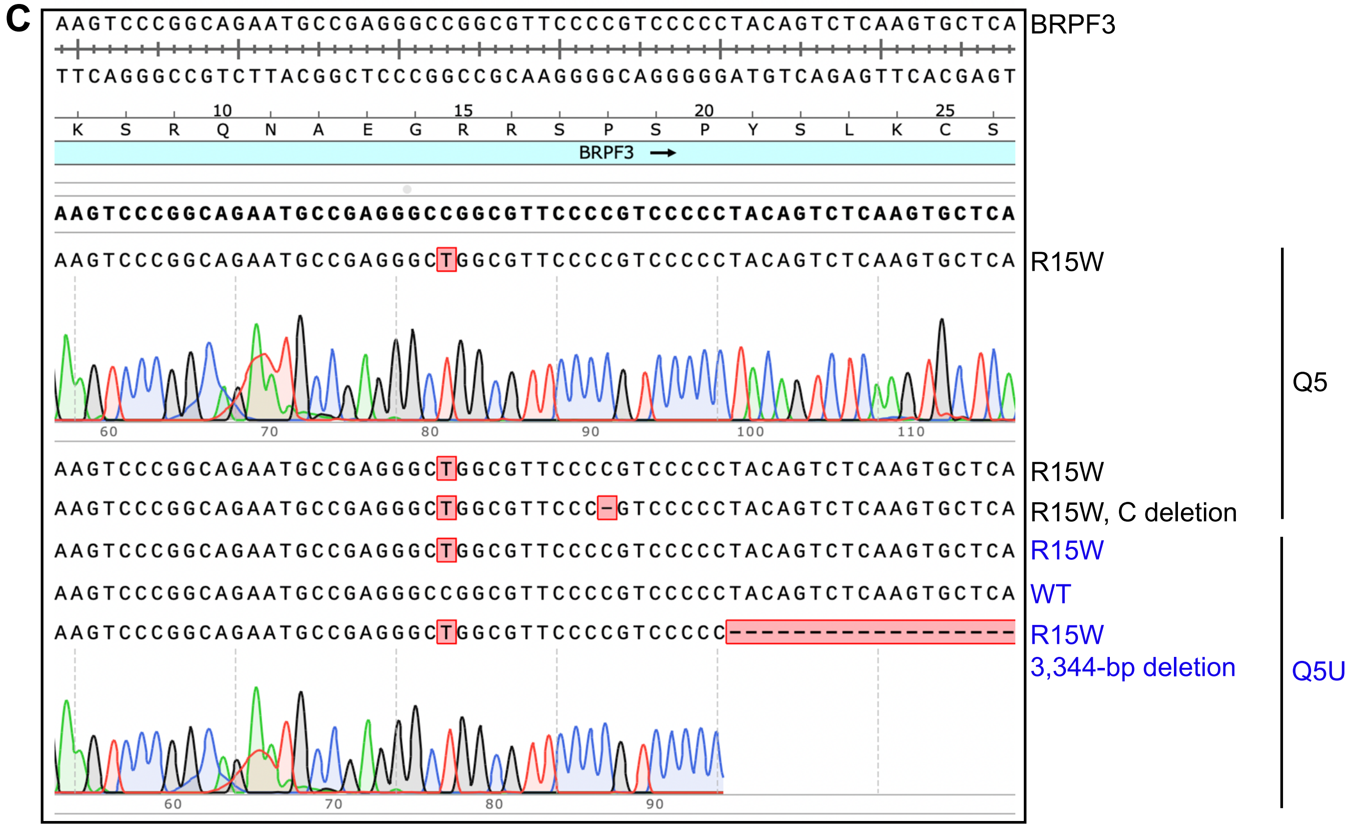

D

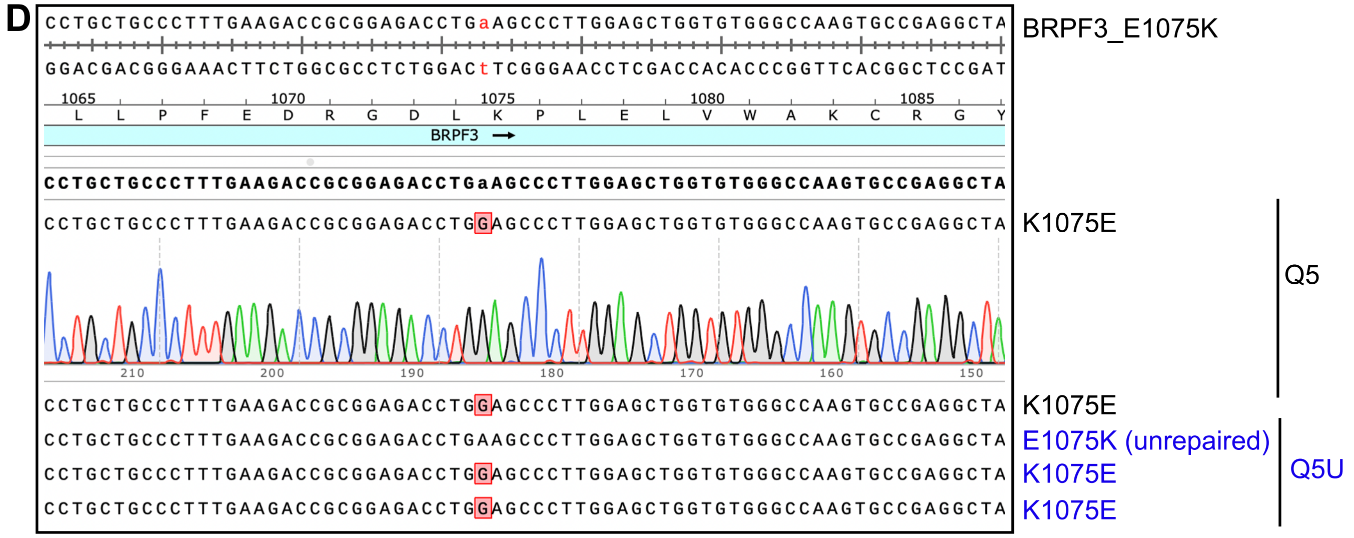

Figure S5

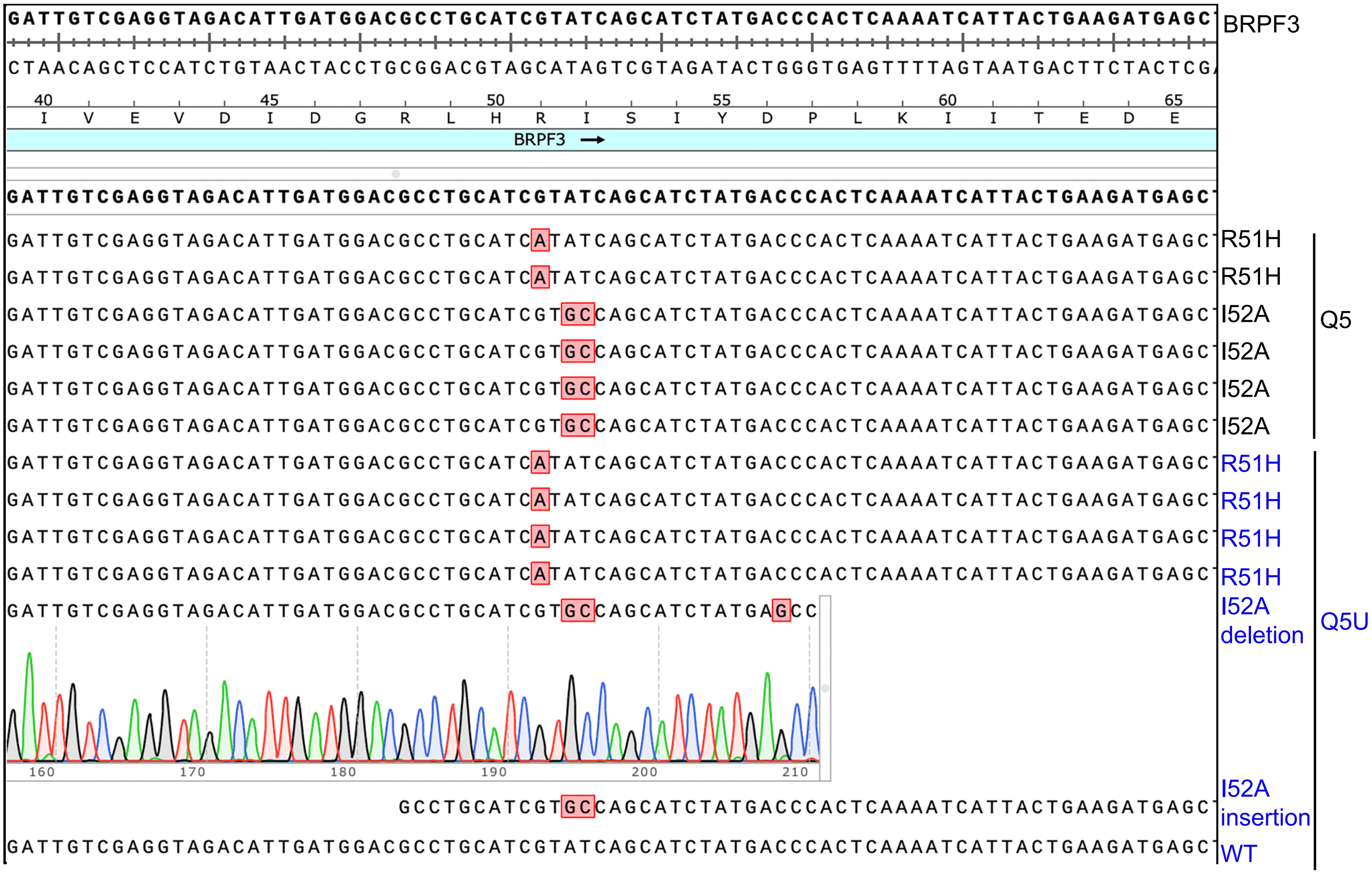
GATTGTCGAGGTAGACATTGATGGACGCCTGCATCGTATCAGCATCTATGACCCACTCAAAATCATTACTGAAGATGAGC
BRPF3
CTAACAGCTCCATCTGTAACTACCTGCGGACGTAGCATAGTCGTAGATACTGGGTGAGTTTTAGTAATGACTTCTACTCG
40 45 50 55 60 65
I V E V D I D G R L H R I S I Y D P L K I I T E D E
BRPF3
R51H
R51H
I52A
I52A
I52A
I52A
Q5
R51H
R51H
R51H
R51H
I52A deletion
Q5U
160 170 180 190 200 210
I52A insertion
WT

Figure S6

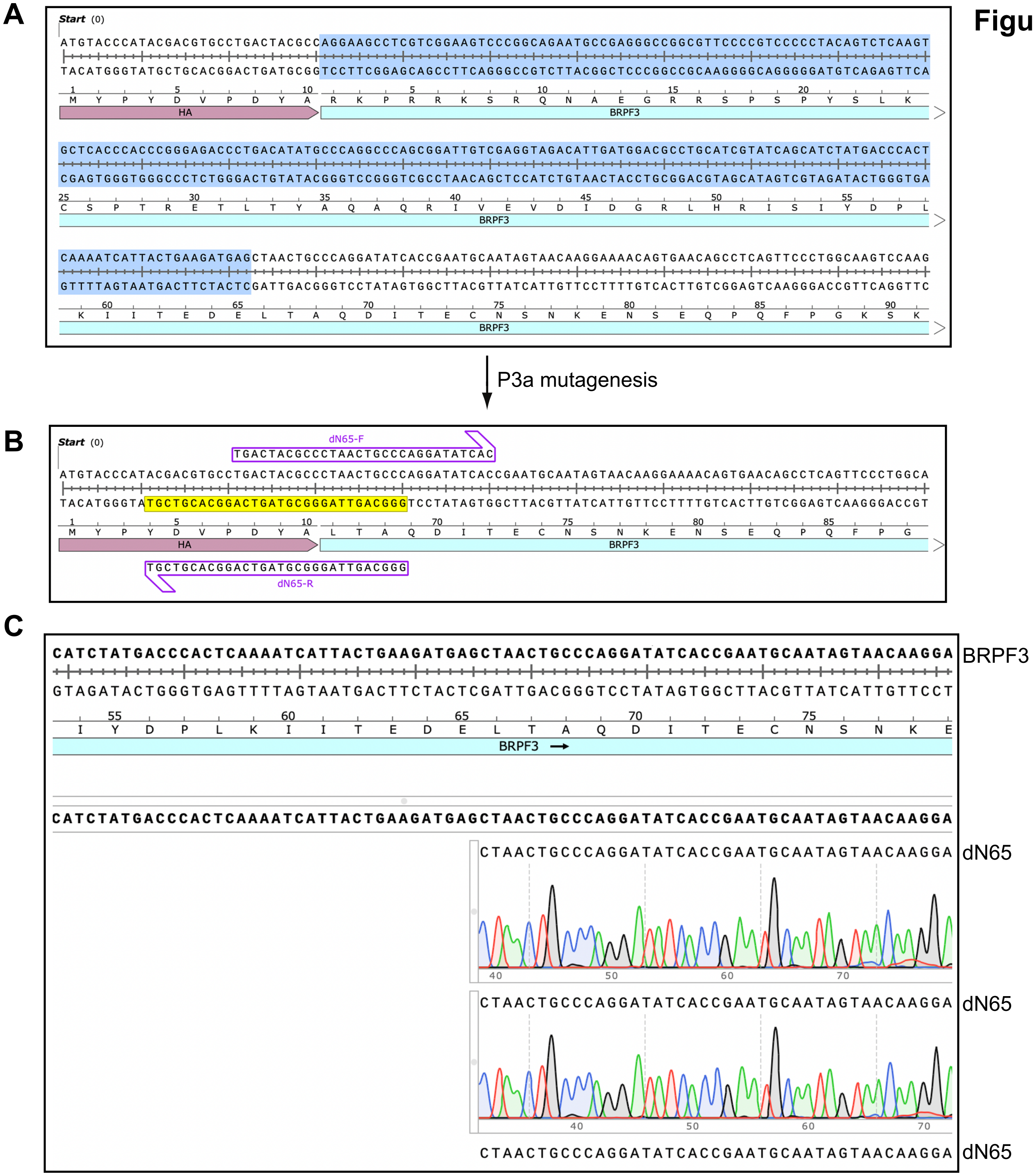
A
Start (0)
HA
BRPF3
P3a mutagenesis
B
Start (0)
dN65-F
TGACTACGCCCTAACTGCCCAGGATATCAC
TGCTGCACGGACTGATGCGGGATTGACGGG
dN65-R
C
CATCTATGACCCACTCAAAATCATTACTGAAGATGAGCTAACTGCCCAGGATATCACCGAATGCAATAGTAACAAGGA
BRPF3
GTAGATACTGGGTGAGTTTTAGTAATGACTTCTACTCGATTGACGGGTCCTATAGTGGCTTACGTTATCATTGTTCCT
I Y D P L K I I T E D E L T A Q D I T E C N S N K E
CTAACTGCCCAGGATATCACCGAATGCAATAGTAACAAGGA
dN65
dN65
dN65

Figure S7

A

Mutation (D614G)	Mutants/colonies sequenced (%)
R346T	3/3 (100%)
Q493E	2/3 (66.7%)
L981F	3/3 (100%)
V1104L	3/3 (100%)
Total	11/12 (91.7%)

B

Mutation (Omicron)	Mutants/colonies sequenced (%)
R346T	1/3 (33.3%)
Q493E	0/3 (0%)
L455S;L455S_F456L	0;2/3 (66.7%)
V1104L	2/3 (66.7%)
Total	5/12 (41.7%)

C

D

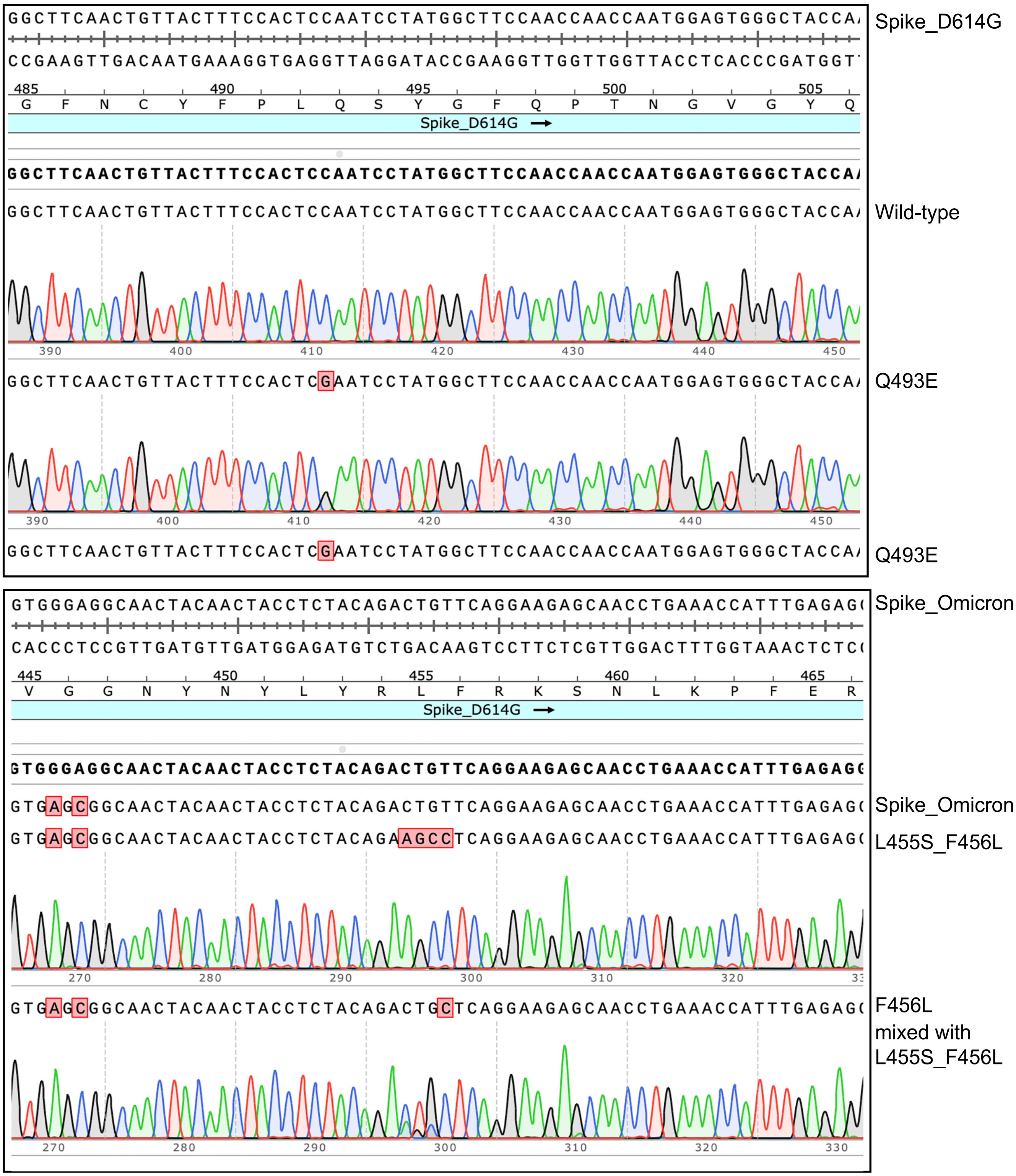

Figure S8

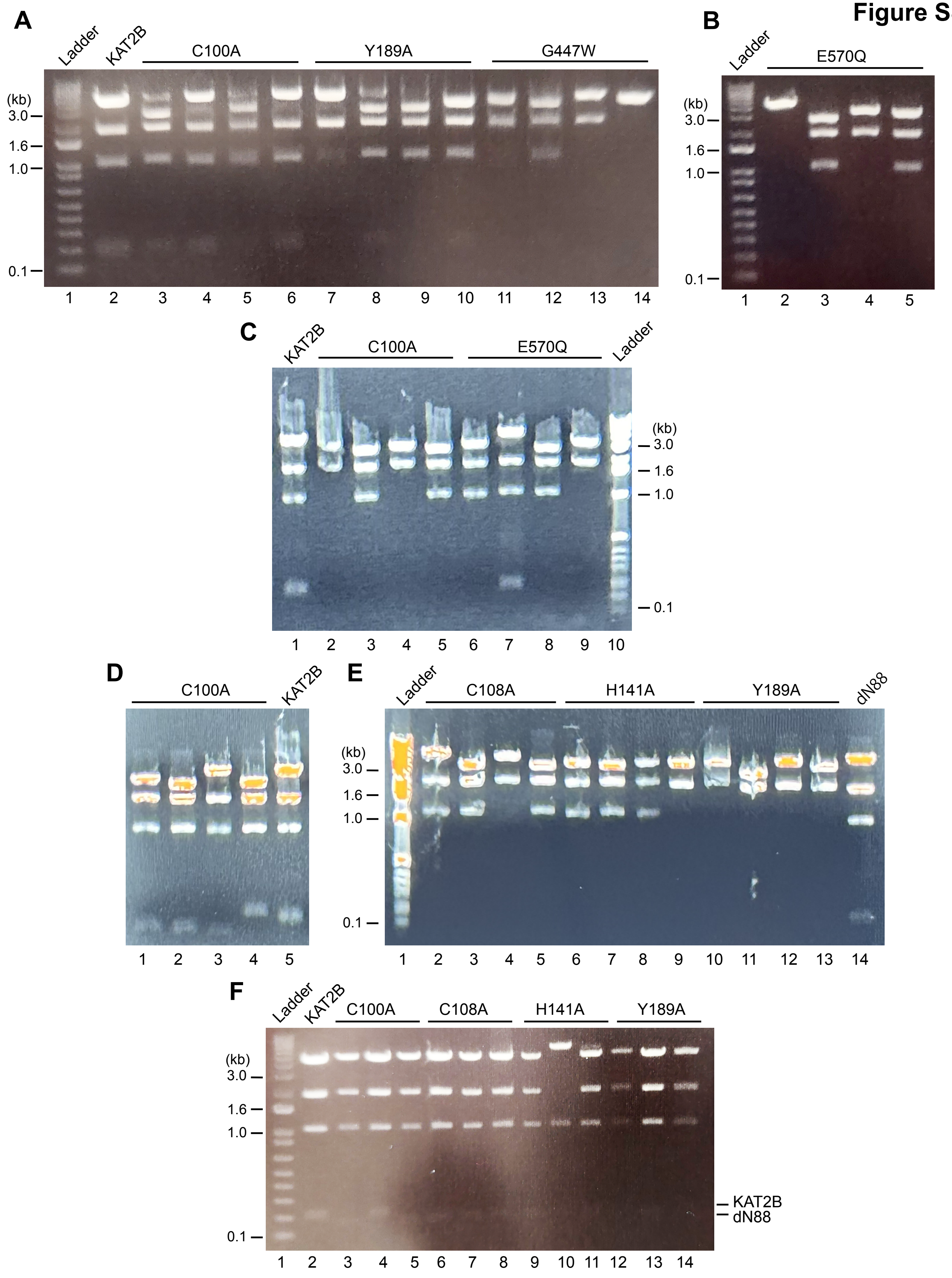
A
Ladder
KAT2B
C100A
Y189A
G447W
(kb)
3.0
1.6
1.0
0.1
1 2 3 4 5 6 7 8 9 10 11 12 13 14
B
Ladder
E570Q
(kb)
3.0
1.6
1.0
0.1
1 2 3 4 5
C
KAT2B
C100A
E570Q
Ladder
(kb)
3.0
1.6
1.0
0.1
1 2 3 4 5 6 7 8 9 10
D
C100A
KAT2B
1 2 3 4 5
E
Ladder
C108A
H141A
Y189A
dN88
(kb)
3.0
1.6
1.0
0.1
1 2 3 4 5 6 7 8 9 10 11 12 13 14
F
Ladder
KAT2B
C100A
C108A
H141A
Y189A
(kb)
3.0
1.6
1.0
0.1
KAT2B
dN88
1 2 3 4 5 6 7 8 9 10 11 12 13 14

Figure S9

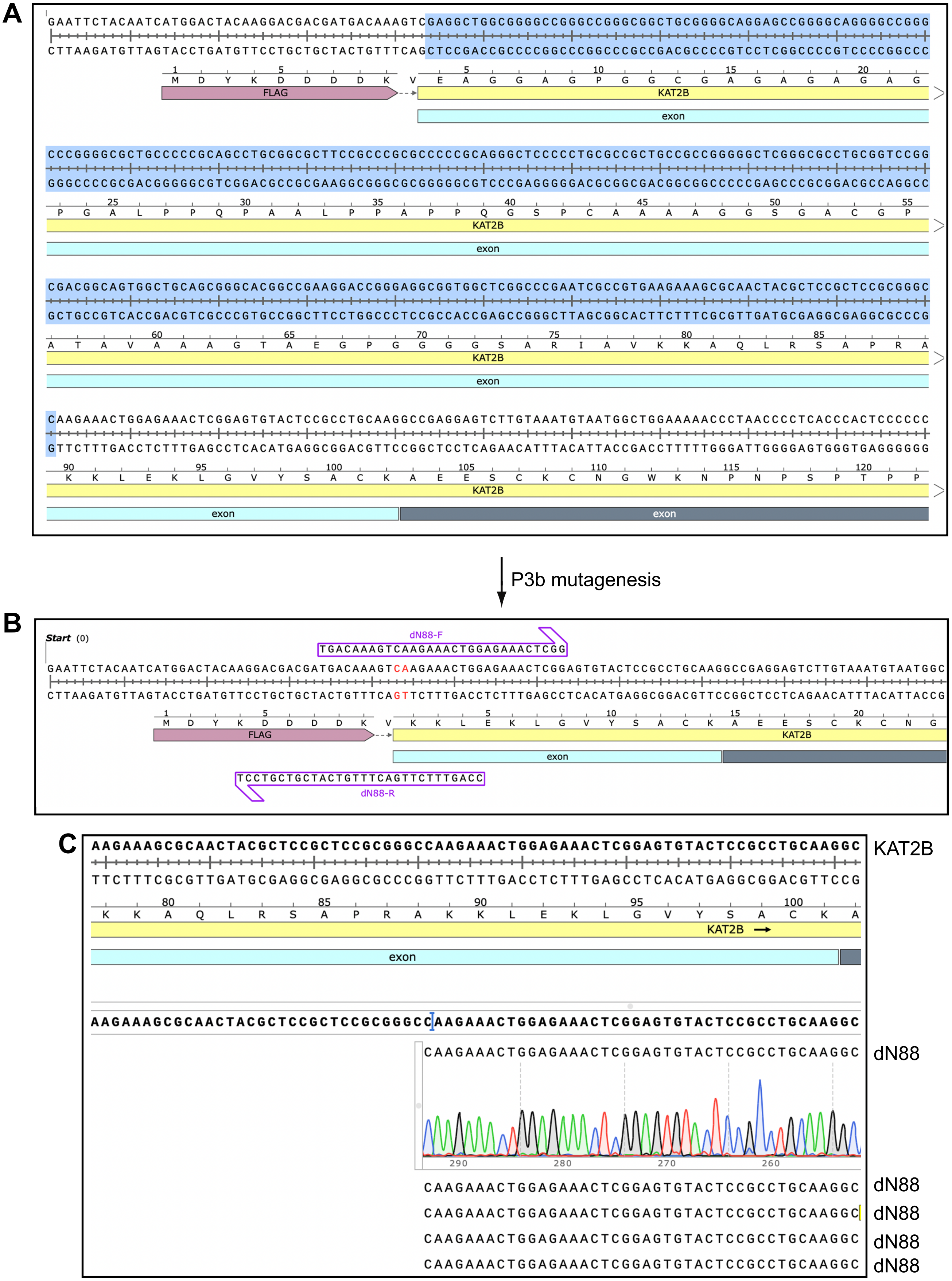
A
FLAG
KAT2B
exon
P3b mutagenesis
B
Start (0)
dN88-F
TGACAAAGTCAAGAAACTGGAGAAACTCGG
dN88-R
TCCTGCTGCTACTGTTTCAGTTCTTTGACC
C
KAT2B
dN88
dN88
dN88
dN88
dN88

Figure S10

A

Mutation (KAT6A)	Mutants/colonies sequenced (%)
K604R	2/3 (66.7%)
N621S	3/3 (100%)
S670P	1/3 (33.3%)
L1060*	3/3 (100%)
Total	9/12 (75%)

B

Mutation (CBP/p300)	Mutants/colonies sequenced (%)
W1466C;Y1467N (CBP)	0/3 (0%)
Y1503D;Y1503H (CBP)	2/3 (66.7%)
C1204S; C1204R (p300)	2/3 (66.7%)
D1399Y;D1399H (p300)	3/3 (100%)
Total	7/12 (58.3%)

C

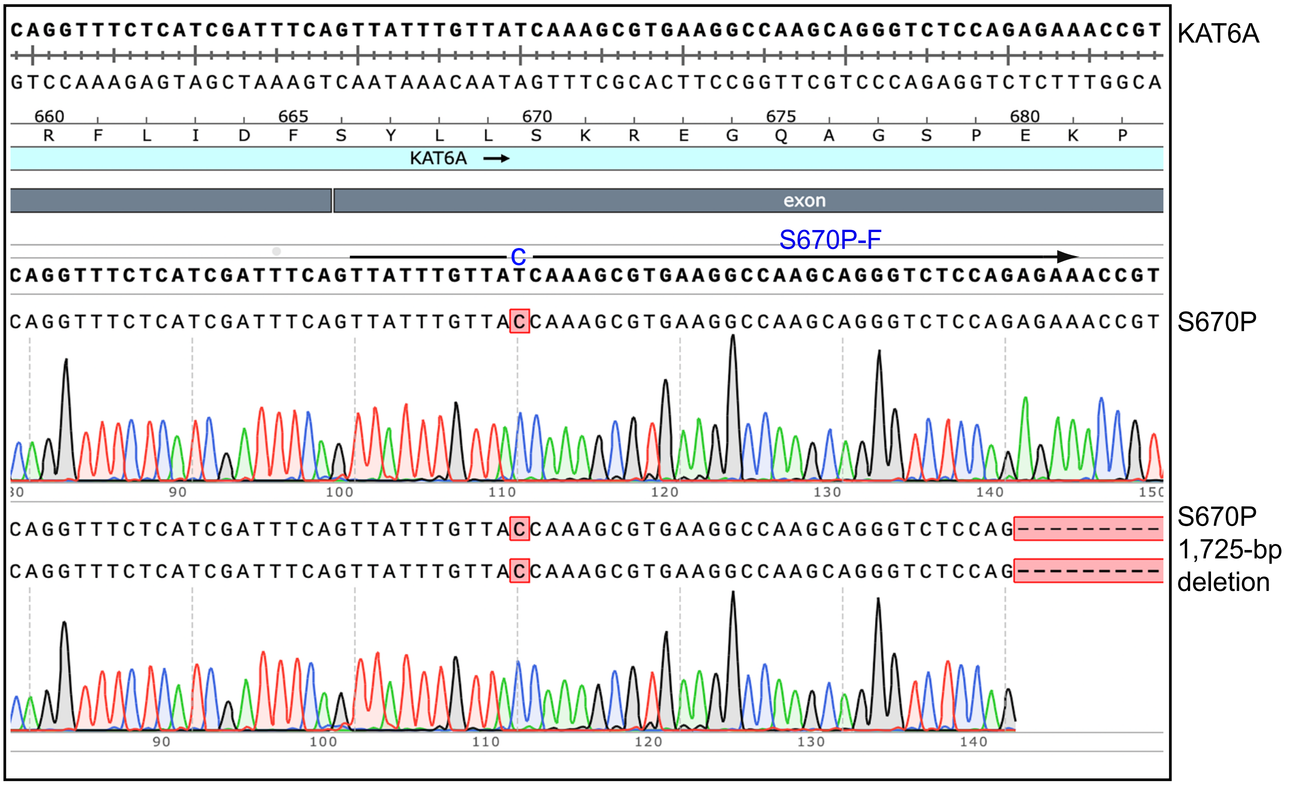

D

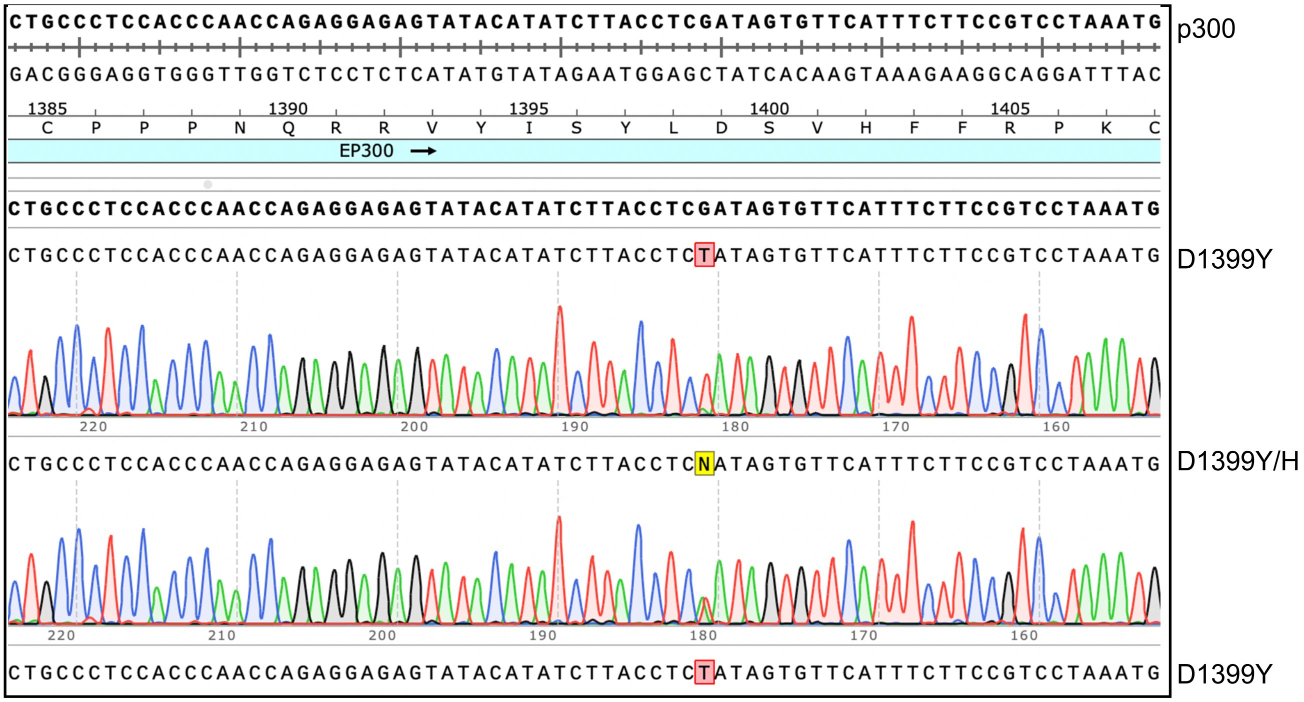

Figure S11

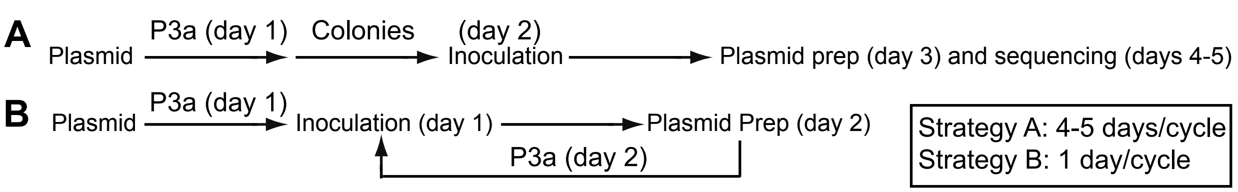

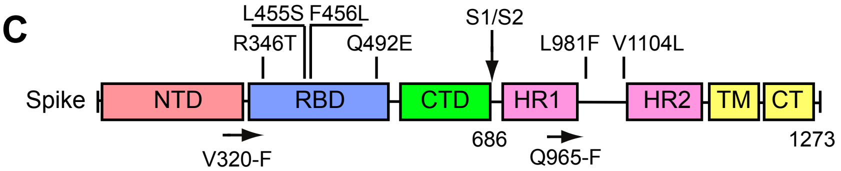

D

Mutation (Spike)	D614G	Omicron
R346T	7/10 (70%)	4/4 (100%)
L455S/L455S_F456L	4.5;3.5/10 (80%)	2;1/4 (75%)
Q493E	8/10 (80%)	4/4 (100%)
L981F	8/10 (80%)	n/a
V1104L	8/10 (80%)	2/4 (50%)
Total*	39/50 sites (78%)	13/16 sites (81.3%)
Multi-mutant*	6/10 (60%)	2/4 (50%)

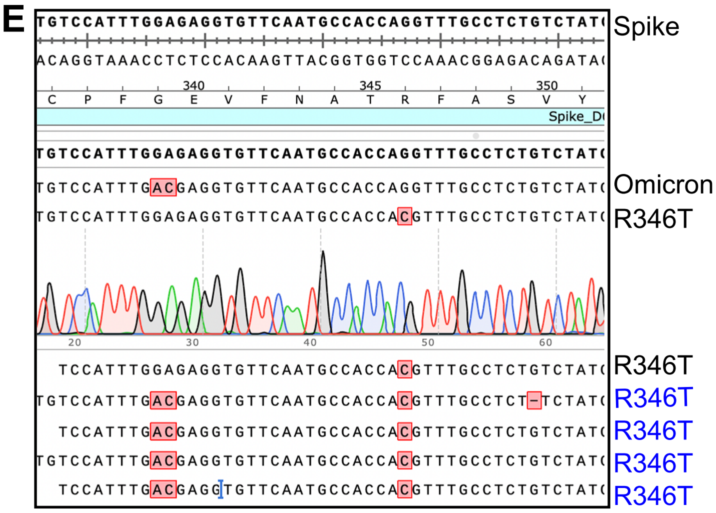

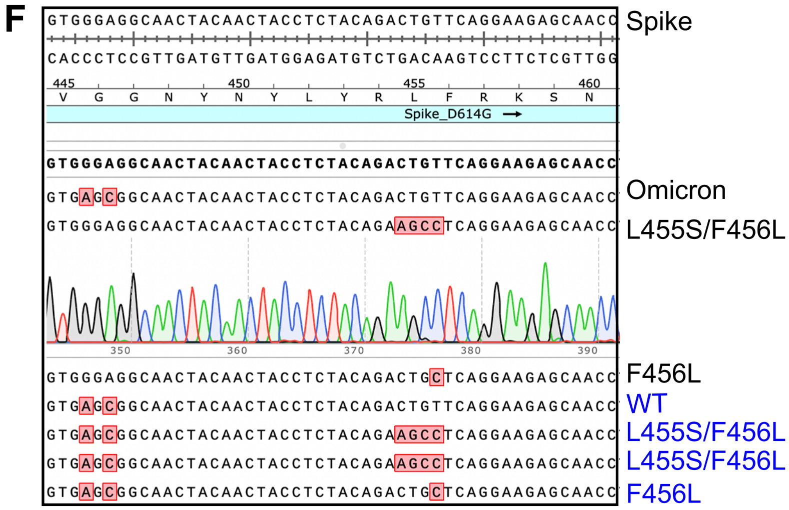

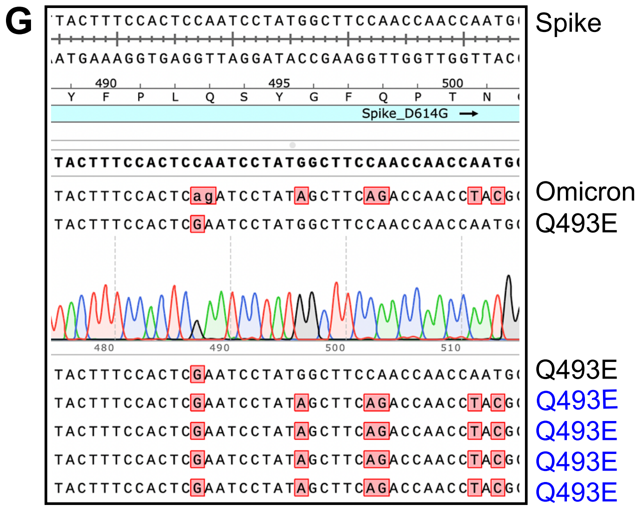

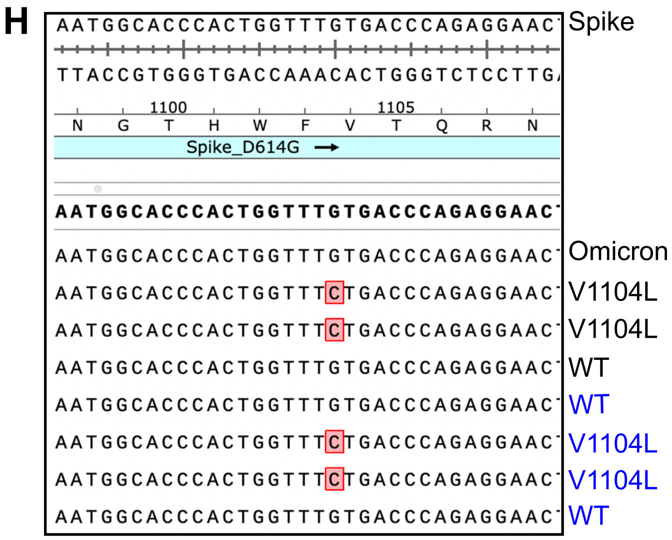

Figure S12

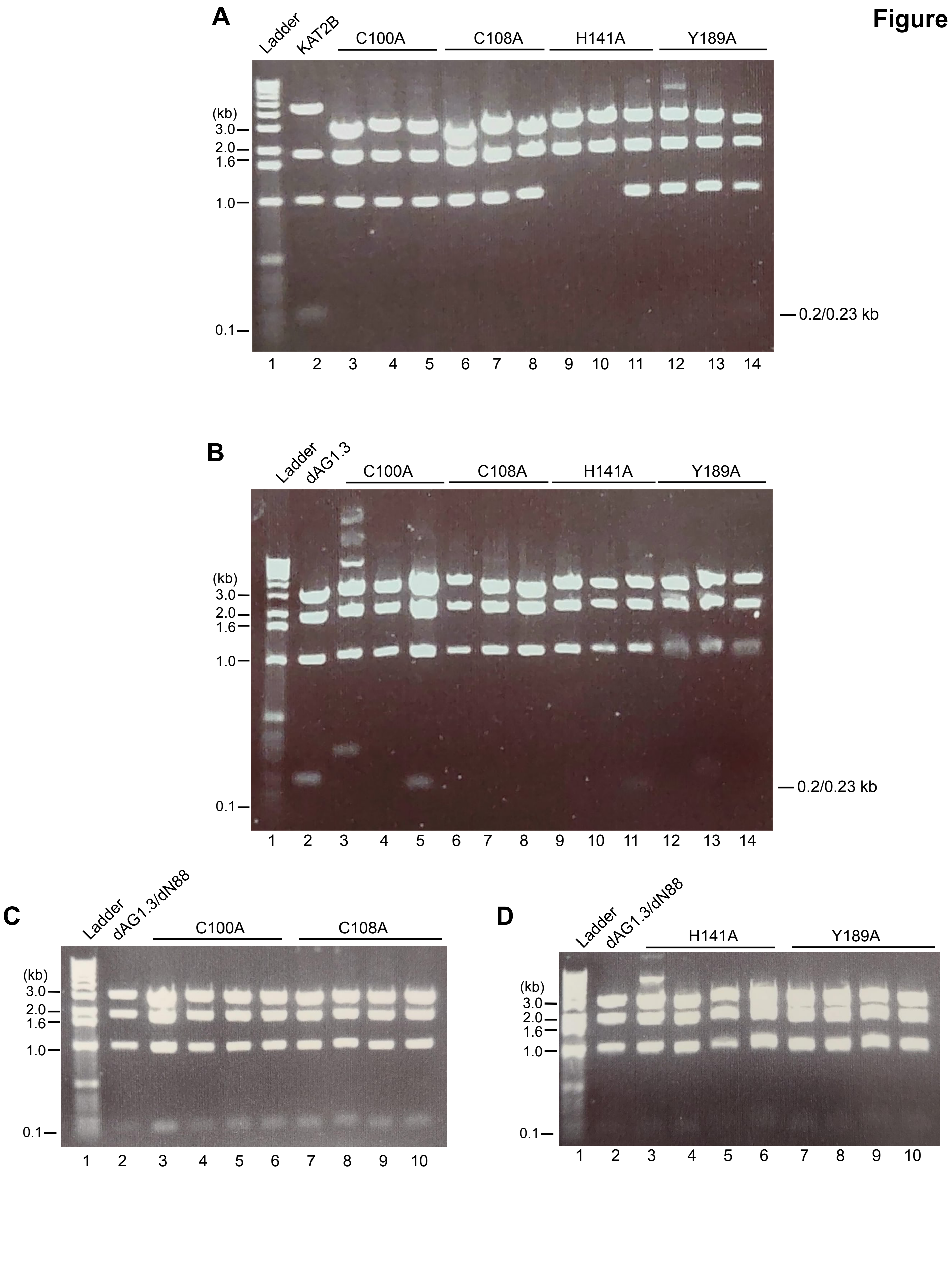
A
Ladder
KAT2B
C100A
C108A
H141A
Y189A
(kb)
3.0
2.0
1.6
1.0
0.1
0.2/0.23 kb
1 2 3 4 5 6 7 8 9 10 11 12 13 14
B
Ladder
dAG1.3
C100A
C108A
H141A
Y189A
(kb)
3.0
2.0
1.6
1.0
0.1
0.2/0.23 kb
1 2 3 4 5 6 7 8 9 10 11 12 13 14
C
Ladder
dAG1.3/dN88
C100A
C108A
(kb)
3.0
2.0
1.6
1.0
0.1
1 2 3 4 5 6 7 8 9 10
D
Ladder
dAG1.3/dN88
H141A
Y189A
(kb)
3.0
2.0
1.6
1.0
0.1
1 2 3 4 5 6 7 8 9 10

Figure S13

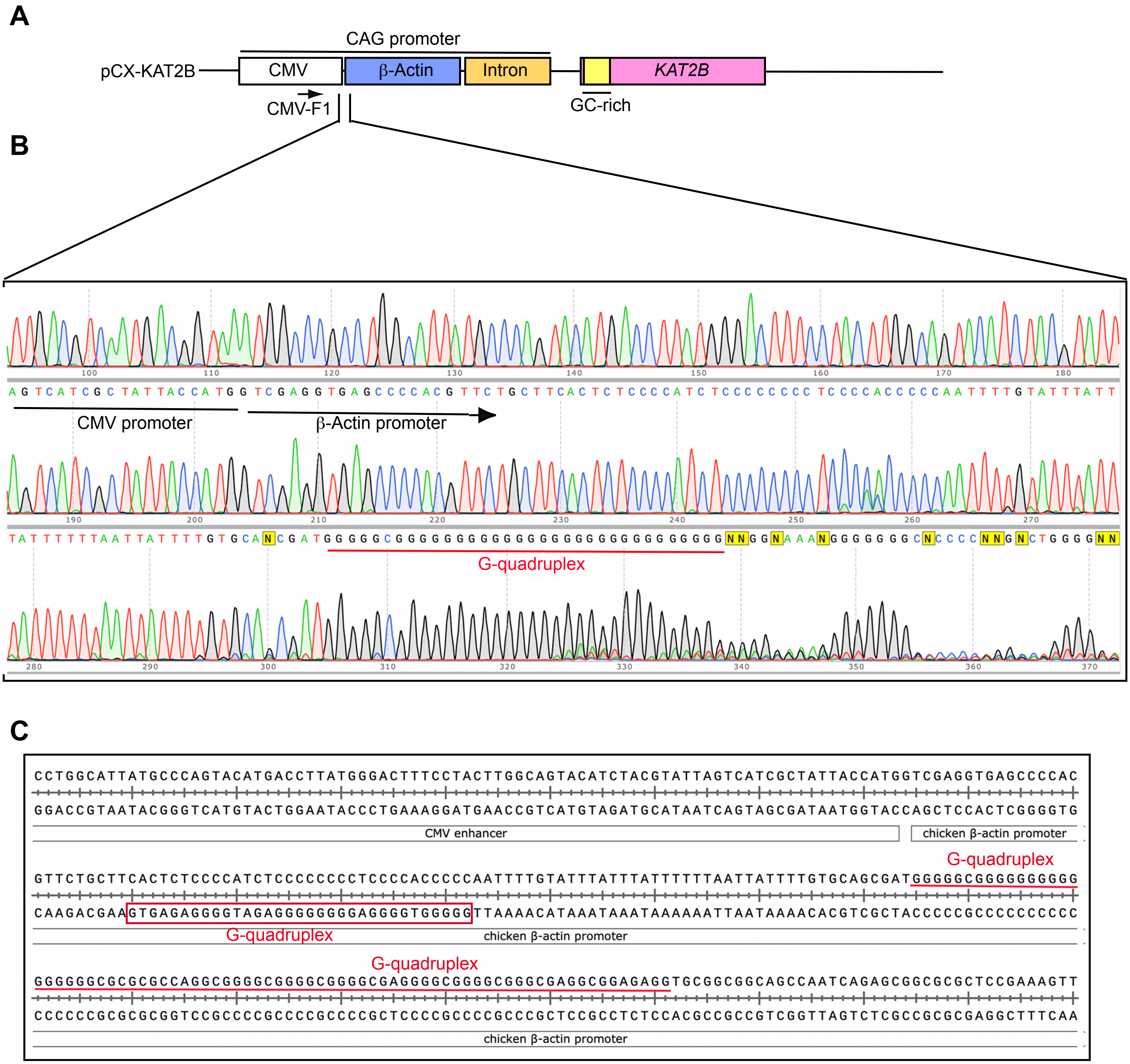
A
CAG promoter
pCX-KAT2B
CMV
β-Actin
Intron
KAT2B
CMV-F1
GC-rich
B
CMV promoter
β-Actin promoter
G-quadruplex
C
CMV enhancer
chicken β-actin promoter
G-quadruplex
G-quadruplex
chicken β-actin promoter
G-quadruplex
chicken β-actin promoter

Figure S14

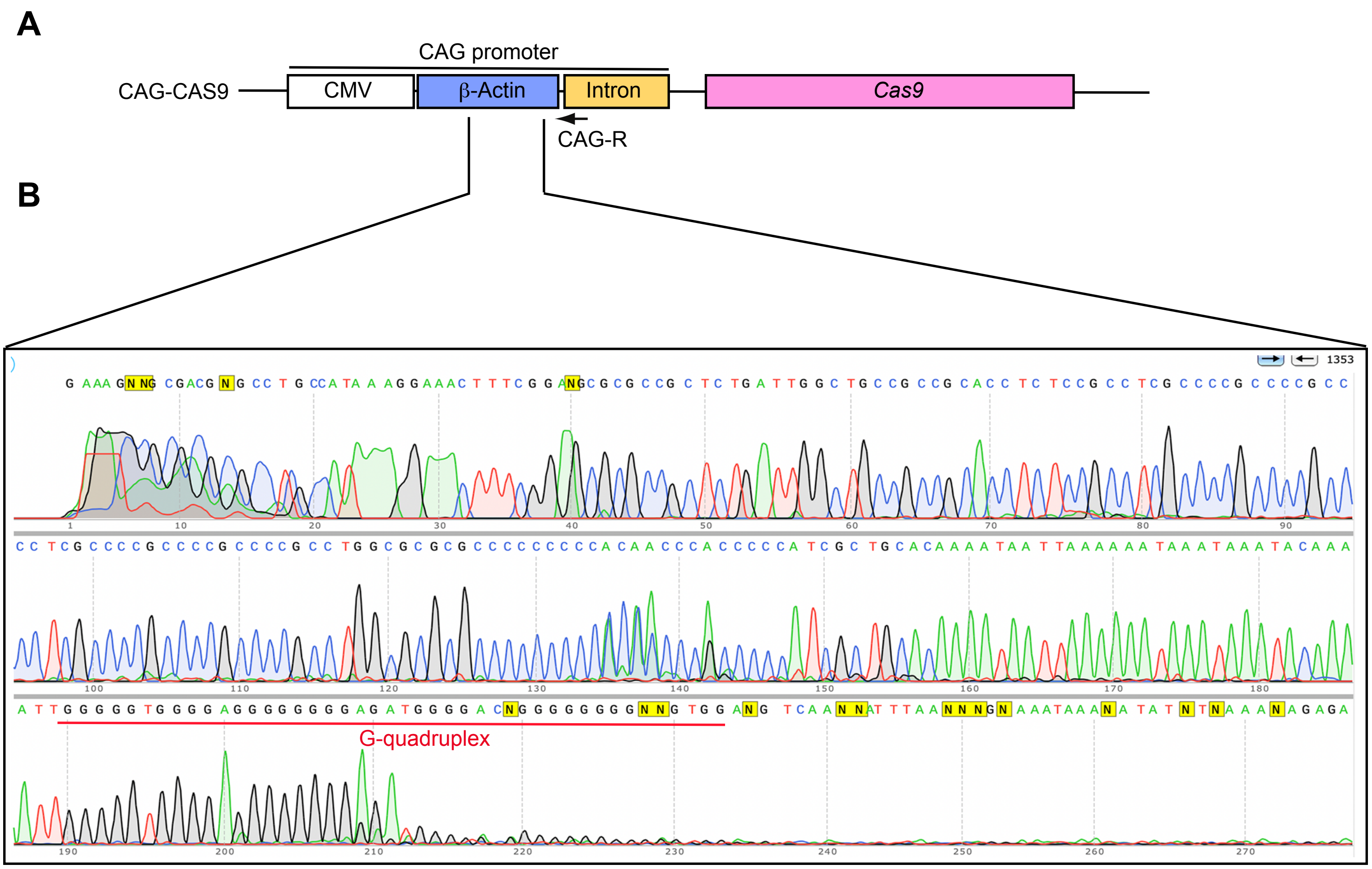
A
CAG promoter
CAG-CAS9
CMV
β-Actin
Intron
Cas9
CAG-R
B
G-quadruplex

Figure S15

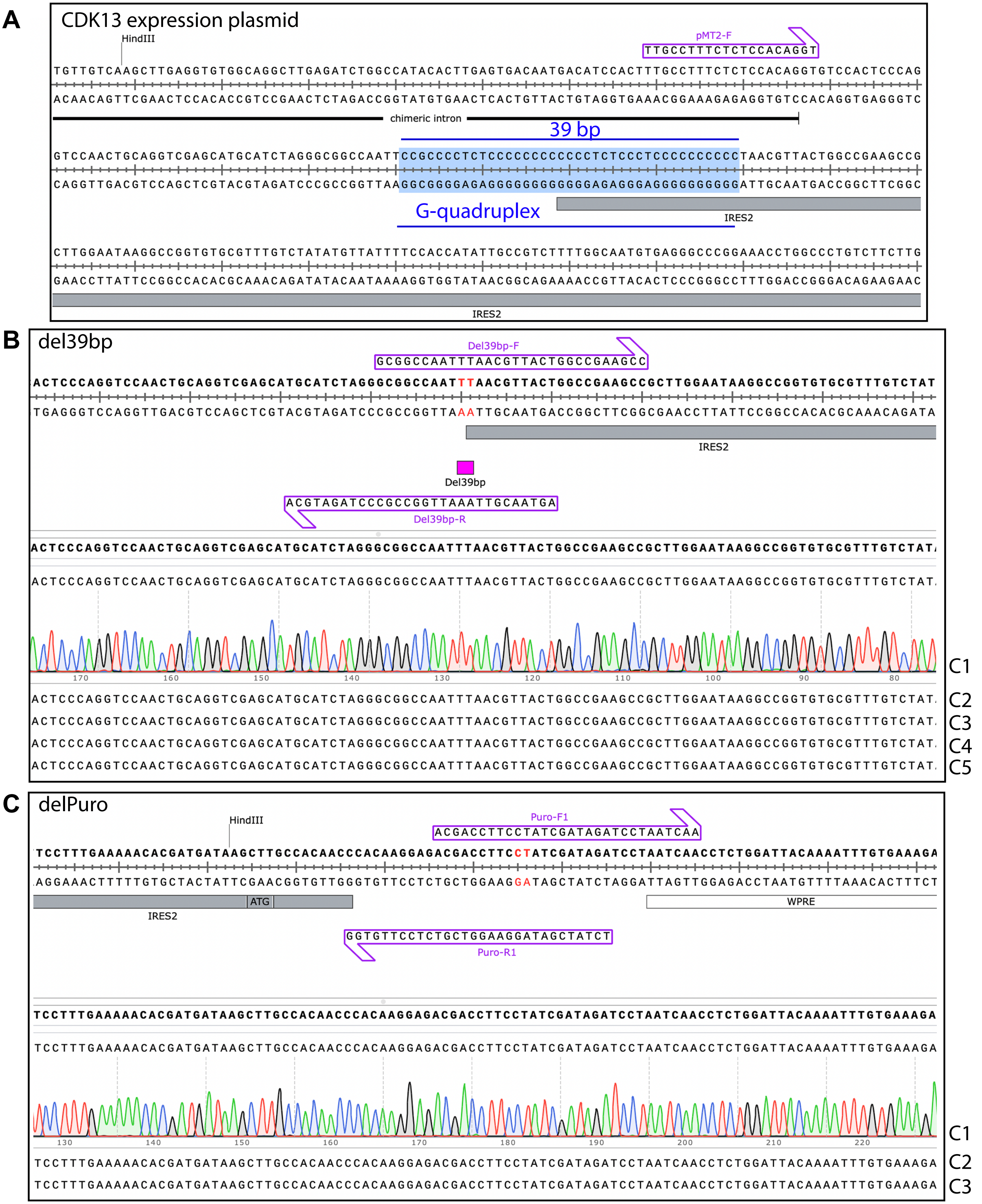

A
CDK13 expression plasmid
HindIII
pMT2-F
TTGCCTTTCTCTCCACAGGT
TGTTGTCAAGCTTGAGGTGTGGCAGGCTTGAGATCTGGCCATACACTTGAGTGACAATGACATCCACTTTGCCTTTCTCTCCACAGGTGTCCACTCCCAG
ACAACAGTTCGAACTCCACACCGTCCGAACTCTAGACCGGTATGTGAACTCACTGTTACTGTAGGTGAAACGGAAAGAGAGGTGTCCACAGGTGAGGGTC
chimeric intron
39 bp
GTCCAACTGCAGGTCGAGCATGCATCTAGGGCGGCCAATTCCGCCCCTCTCCCCCCCCCCCTCTCCCTCCCCCCCCCTAACGTTACTGGCCGAAGCCG
CAGGTTGACGTCCAGCTCGTACGTAGATCCCGCCGGTTAAGGCGGGGAGAGGGGGGGGGGGAGAGGGAGGGGGGGGGATTGCAATGACCGGCTTCGGC
G-quadruplex
IRES2
CTTGGAATAAGGCCGGTGTGCGTTTGTCTATATGTTATTTTCCACCATATTGCCGTCTTTTGGCAATGTGAGGGCCCGGAAACCTGGCCCTGTCTTCTTG
GAACCTTATTCCGGCCACACGCAAACAGATATACAATAAAAGGTGGTATAACGGCAGAAAACCGTTACACTCCCGGGCCTTTGGACCGGGACAGAAGAAC
IRES2
B
del39bp
Del39bp-F
GCGGCCAATTTAACGTTACTGGCCGAAGCC
ACTCCCAGGTCCAACTGCAGGTCGAGCATGCATCTAGGGCGGCCAATTTAACGTTACTGGCCGAAGCCGCTTGGAATAAGGCCGGTGTGCGTTTGTCTAT
TGAGGGTCCAGGTTGACGTCCAGCTCGTACGTAGATCCCGCCGGTTAAATTGCAATGACCGGCTTCGGCGAACCTTATTCCGGCCACACGCAAACAGATA
IRES2
Del39bp
ACGTAGATCCCGCCGGTTAAATTGCAATGA
Del39bp-R
ACTCCCAGGTCCAACTGCAGGTCGAGCATGCATCTAGGGCGGCCAATTTAACGTTACTGGCCGAAGCCGCTTGGAATAAGGCCGGTGTGCGTTTGTCTAT
ACTCCCAGGTCCAACTGCAGGTCGAGCATGCATCTAGGGCGGCCAATTTAACGTTACTGGCCGAAGCCGCTTGGAATAAGGCCGGTGTGCGTTTGTCTAT
170 160 150 140 130 120 110 100 90 80
C1
ACTCCCAGGTCCAACTGCAGGTCGAGCATGCATCTAGGGCGGCCAATTTAACGTTACTGGCCGAAGCCGCTTGGAATAAGGCCGGTGTGCGTTTGTCTAT C2
ACTCCCAGGTCCAACTGCAGGTCGAGCATGCATCTAGGGCGGCCAATTTAACGTTACTGGCCGAAGCCGCTTGGAATAAGGCCGGTGTGCGTTTGTCTAT C3
ACTCCCAGGTCCAACTGCAGGTCGAGCATGCATCTAGGGCGGCCAATTTAACGTTACTGGCCGAAGCCGCTTGGAATAAGGCCGGTGTGCGTTTGTCTAT C4
ACTCCCAGGTCCAACTGCAGGTCGAGCATGCATCTAGGGCGGCCAATTTAACGTTACTGGCCGAAGCCGCTTGGAATAAGGCCGGTGTGCGTTTGTCTAT C5
C
delPuro
HindIII
Puro-F1
ACGACCTTCCTATCGATAGATCCTAATCAA
TCCTTTGAAAAACACGATGATAAGCTTGCCACAACCCACAAGGAGACGACCTTCCTATCGATAGATCCTAATCAACCTCTGGATTACAAAATTTGTGAAAGA
AGGAAACTTTTTGTGCTACTATTCGAACGGTGTTGGGTGTTCCTCTGCTGGAAGGATAGCTATCTAGGATTAGTTGGAGACCTAATGTTTTAAACACTTTCT
ATG
IRES2
WPRE
GGTGTTCCTCTGCTGGAAGGATAGCTATCT
Puro-R1
TCCTTTGAAAAACACGATGATAAGCTTGCCACAACCCACAAGGAGACGACCTTCCTATCGATAGATCCTAATCAACCTCTGGATTACAAAATTTGTGAAAGA
TCCTTTGAAAAACACGATGATAAGCTTGCCACAACCCACAAGGAGACGACCTTCCTATCGATAGATCCTAATCAACCTCTGGATTACAAAATTTGTGAAAGA
130 140 150 160 170 180 190 200 210 220
C1
TCCTTTGAAAAACACGATGATAAGCTTGCCACAACCCACAAGGAGACGACCTTCCTATCGATAGATCCTAATCAACCTCTGGATTACAAAATTTGTGAAAGA C2
TCCTTTGAAAAACACGATGATAAGCTTGCCACAACCCACAAGGAGACGACCTTCCTATCGATAGATCCTAATCAACCTCTGGATTACAAAATTTGTGAAAGA C3

Figure S16

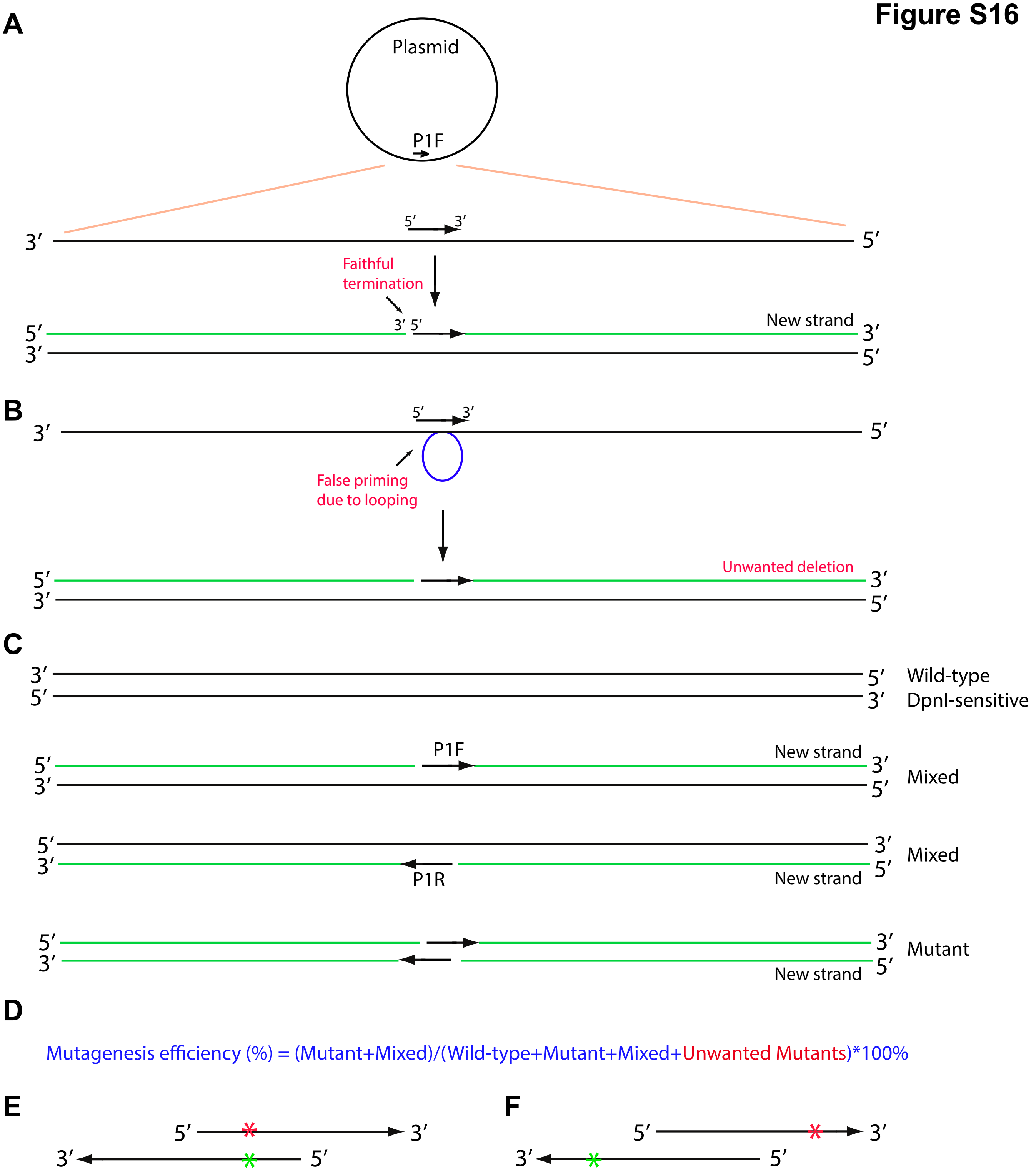
A
Plasmid
P1F
5′
3′
3′
5′
Faithful
termination
3′ 5′
New strand
5′
3′
3′
5′
B
5′
3′
3′
5′
False priming
due to looping
Unwanted deletion
5′
3′
3′
5′
C
3′
5′
5′
3′
Wild-type
DpnI-sensitive
P1F
New strand
5′
3′
3′
5′
Mixed
5′
3′
3′
5′
Mixed
P1R
New strand
5′
3′
3′
5′
Mutant
New strand
D
Mutagenesis efficiency (%) = (Mutant+Mixed)/(Wild-type+Mutant+Mixed+Unwanted Mutants)*100%
E
5′
3′
3′
5′
F
5′
3′
3′
5′

Table S1. Primers to engineer and sequence the mutants of BRPF3, KAT2B and two SARS-CoV-2 spike variants.

Mutation	Forward primer (-F)	Reverse Primer (-R)
	**BRPF3**	
dN65	TGACTACGCCCTAACTGCCCAGGATATCAC	GGGCAGTTAGGGCGTAGTCAGGCACGTCGT
dN126	TGACTACGCCATCCAGCCAGAAGCACCCCC	CTGGCTGGATGGCGTAGTCAGGCACGTCGT
	**KAT2B**	
G48C	GCCGCCGGGTGCTCGGGCGCCTGCGGTCC	GGGCAGTTAGGGCGTAGTCAGGCACGTCGT
C100A	GTACTCCGCCGCTAAGGCCGAGGAGTCTTGTA	CCTCGGCCTTAGCGGCGGAGTACACTCCGAGTT
C108A	GTCTTGTAAAGCTAATGGCTGGAAAAACCCTA	TCCAGCCATTAGCTTTACAAGACTCCTCGGCCT
H141A	GAGTTGTAGCGCTGCCCTAGCTGCTCATGTTT	AGCTAGGGCAGCGCTACAACTCCGACAGGATT
Y189A	AGTTTATTTCGCTCTATTTAAGCTCTTGAGAAA	CTTAAATAGAGCGAAATAAACTTGTTTGGTAT
E570Q	GGATTCACACAGATTGTCTTCTGTGCTGTA	AGACAATCTGTGTGAATCCTTGAGATGGGA
dN88	TGACAAAGTCAAGAAACTGGAGAAACTCGG	CCAGTTTCTTGACTTTGTCATCGTCGTCCT
dN123	TGACAAAGTCGACCTGCAGCAAATAATTGT	GCTGCAGGTCGACTTTGTCATCGTCGTCCT
d1.3-kb	TGTGCTGTCTCATCATTTTGGCAAAGAATT (pCX-F);<br>TATTACCATGTGTCTCATCATTTTGGCAAA (pCX-F1)	TGATGAGACACATGGTAATAGCGATGACTA
	**Sequencing primers**	
V320-F	ACTTCAGGGTCCAACCAACA	
G566-R	AATGTCCCTGCCAAACTGTT	
Q965-F	ACACCCTGGTGAAGCAACTT	
CMV-F	AATGACGTATGTTCCCATAGT	
KAT2B-R	CGAGCCGCAGCCATTGCCTT	

[1]Unless indicated in parentheses, all primers follow the (-F/-R) labelling system except for pCX-F1.

Table S2. Primers to engineer and sequence the CDK13 and Cas9 mutants.

Mutation	Forward primer (-F)	Reverse Primer (-R)
	**CDK13**	
Ala162fs	TGGGGGGGGgCCAGCGCGGCAACGGCGGCG (-F);<br>GCTGCTGGGtGGtGgCCAGCGCGGCAACGGCGGCG (-F1)	CCGCGCTGGcCCCCCCCCAGCAGCAGCCCC (-R);<br>CCGCGCTGGcCaCCaCCCAGCAGCAGCCCCTGCTC (-R1)
S229P	AGCGAGGCCcCCAAGTCCCGCAGCCGCCAC	GGGACTTGGgGGCCTCGCTGCCACCGCGCT
S248N	TCGCCAAGAaCGGCAGCAGCAGCAGCAGCG	CTGCTGCCGtTCTTGGCGACCTCGGCCCGT
V327M	GACAGCCCGaTGTCCCACAGGGCCTCTCAG	TGTGGGACAtCGGGCTGTCGTCCCGGCCTC
G714R	ATTGGAGAAcGTACTTACGGACAAGTTTAC	CGTAAGTACgTTCTCCAATAATTCCGATGA
N842D/S	AAATGTTCCgATATCCTTCTAAATAATAGA	AGAAGGATAcTGGAACATTTAATATCTCTA
d07-394	CAGCTCGGACTCTCGCAGTCCCTACAGCCC	GACTGCGAGAGTCCGAGCTGCTCGGCATGG
del39bp	GCGGCCAATTTAACGTTACTGGCCGAAGCC	AGTAACGTTAAATTGGCCGCCCTAGATGCA
delPuro	ACGACCTTCCTATCGATAGATCCTAATCAA (-F1)	TCTATCGATAGGAAGGTCGTCTCCTTGTGG (-R1)
	**Cas9 (1.1)**	
K526D	CGAGCTGACCgatGTGAAATACGTGACCGAGGG	CGTATTTCACatcGGTCAGCTCGTTATACACGG
K562D	AGTGACCGTGgatCAGCTGAAAGAGGACTACTT	CTTTCAGCTGatcCACGGTCACTTTCCGGTTGG
R691A	CTTCGCCAACgccAACTTCATGCAGCTGATCCA	GCATGAAGTTggcGTTGGCGAAGCCGTCGGACT
F846Y	CTCAGAGCTaTCTGGCCGACGACTCCATCG	TCGGCCAGAtAGCTCTGAGGCACGATATGGT
I852F	GACGACTCCtTCGACAACAAGGTGCTGACC	TGTTGTCGAaGGAGTCGTCGGCCAGAAAGC
E1007L	CTGGAAAGCctGTTCGTGTACGGCGACTACA	TACACGAACagGCTTTCCAGCGCAGGGTACT
	**Sequencing primers**	
IRES-F	TGCATTCCTTTGGCGAGAGG	
IRES-R	TGGCTCTCCTCAAGCGTATT	

[1]Unless indicated in parentheses, all primers follow the (-F/-R) labelling system except for those noted with -F1 or -R1.
